# Ghrelin: Central and Peripheral Implications in Anorexia Nervosa

**DOI:** 10.3389/fendo.2013.00015

**Published:** 2013-02-26

**Authors:** Mathieu Méquinion, Fanny Langlet, Sara Zgheib, Suzanne Dickson, Bénédicte Dehouck, Christophe Chauveau, Odile Viltart

**Affiliations:** ^1^UMR INSERM 837, Development and Plasticity of Postnatal BrainLille, France; ^2^Pathophysiology of inflammatory of bone diseases, Université Lille Nord de France-ULCO – Lille 2Boulogne sur Mer, France; ^3^Department of Physiology, Institute of Neuroscience and Physiology, The Sahlgrenska Academy, University of GothenburgGothenburg, Sweden; ^4^Department of Endocrinology, Institute of Neuroscience and Physiology, The Sahlgrenska Academy, University of GothenburgGothenburg, Sweden; ^5^Université Lille Nord de France – Université d’ArtoisLiévin, France; ^6^Université Lille Nord de France-USTL (Lille 1)Villeneuve d’Ascq, France

**Keywords:** ghrelin, anorexia, food intake, energy balance, central alterations, peripheral alterations, reward, animal models

## Abstract

Increasing clinical and therapeutic interest in the neurobiology of eating disorders reflects their dramatic impact on health. Chronic food restriction resulting in severe weight loss is a major symptom described in restrictive anorexia nervosa (AN) patients, and they also suffer from metabolic disturbances, infertility, osteopenia, and osteoporosis. Restrictive AN, mostly observed in young women, is the third largest cause of chronic illness in teenagers of industrialized countries. From a neurobiological perspective, AN-linked behaviors can be considered an adaptation that permits the endurance of reduced energy supply, involving central and/or peripheral reprograming. The severe weight loss observed in AN patients is accompanied by significant changes in hormones involved in energy balance, feeding behavior, and bone formation, all of which can be replicated in animals models. Increasing evidence suggests that AN could be an addictive behavior disorder, potentially linking defects in the reward mechanism with suppressed food intake, heightened physical activity, and mood disorder. Surprisingly, the plasma levels of ghrelin, an orexigenic hormone that drives food-motivated behavior, are increased. This increase in plasma ghrelin levels seems paradoxical in light of the restrained eating adopted by AN patients, and may rather result from an adaptation to the disease. The aim of this review is to describe the role played by ghrelin in AN focusing on its central vs. peripheral actions. In AN patients and in rodent AN models, chronic food restriction induces profound alterations in the « ghrelin » signaling that leads to the development of inappropriate behaviors like hyperactivity or addiction to food starvation and therefore a greater depletion in energy reserves. The question of a transient insensitivity to ghrelin and/or a potential metabolic reprograming is discussed in regard of new clinical treatments currently investigated.

## Introduction

Feeding is a behavior that ensures an adequate and varied supply of nutritional substrates essential to maintain energy levels for basal metabolism, physical activity, growth, and reproduction and hence, for survival of every living organism on Earth. In the case of mammals, that must maintain a stable body temperature, the maintenance of a high metabolic rate requires constant availability of a sufficient amount of energy stores. The tight balance between energy demand and expenditure is fine-tuned by an adapted dialog between homeostatic and hedonic brain systems that are regulated by peripheral signals involved in feeding behavior and energy homeostasis. Mechanisms for feeding control remain a current and crucial scientific subject for understanding the etiology and potential therapeutic approaches for the treatment of food intake disorders that include obesity, on one hand, and severe forms of anorexia nervosa (AN) on the other.

Voluntary anorexia is a disease not unique to man and has even been described in many vertebrate species that favor migration activity (Wang et al., 2006). In this case, surviving food deprivation involves an adaptation of metabolism, such that internal energy stores available at the onset of fasting are used to maintain basal metabolism and physical activity. The biochemical and physiological adaptations that result from a lack of food help to preserve physiological function in order to maintain behaviors like food seeking or predator avoidance and also, to resume all metabolic processes necessary when food becomes available. However, absolute or long term food deprivation observed in nature or in restrictive AN proceeds in stages in which the individual/organism tries to adapt its metabolism to energy costs but that culminates in death, due to exhaustion of energy stores. As clearly described by Wang et al. (2006), the different stages progress from fasting to starvation, but “*The demarcation between these two states is rarely appreciated, perhaps owing to lack of definition. In humans, fasting often refers to abstinence from food, whereas starvation is used for a state of extreme hunger resulting from a prolonged lack of essential nutrients. In other words, starving is a state in which an animal, having depleted energy stores, normally would feed to continue normal physiological processes*.” Briefly, three metabolic phases are described during food deprivation (Wang et al., 2006) where energy metabolic adaptations occur to allow supply of fuel in the different parts of the organism, especially the brain (see Table 1). In regard to these metabolic stages, the transition from fasting to starvation occurs by the end of phase II or the beginning of phase III. Thus, voluntary anorexia as seen in restrictive AN should correspond to phases I and II.

**Table 1:** **Different metabolic phases occurring during food restriction and permitting distinction between fasting and starvation (see Wang et al., 2006)**.

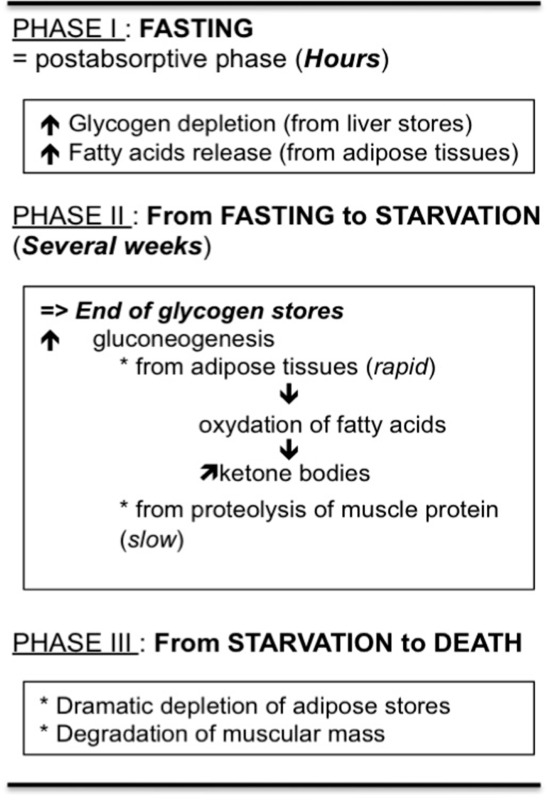

*All the metabolic changes aim to deliver sufficient amount of glucose for different organs and especially for the brain*.

Restrictive AN is a feeding behavior disorder for which severe chronic food restriction causes dramatic physiological and psychological effects that are detrimental for health. AN is most prevalent in women aged of 25 years old or younger (whose BMI reaches values largely below 18.5 kg/m^2^) and is currently the third largest cause of chronic illness in teenagers (Lucas et al., 1991). The prevalence of AN has drastically increased within recent decades. It leads to central and/or peripheral reprograming that permits the individual/organism to endure a reduced energy supply. These drastic conditions not only induce severe weight loss and metabolic disturbance, but also infertility, osteopenia, and osteoporosis. Moreover, AN is increasingly recognized as an addictive behavior disorder. *-Indeed, many of its common primary characteristics – food obsession coupled with food restriction, weight loss, heightened physical activity, and the strong association with mood disorder (such as anxiety or depression), strongly suggest a potential alteration of the central (dopaminergic) reward system.

Anorexia nervosa patients exhibit significant changes in the release of key hormones involved in energy balance and feeding control (Hasan and Hasan, 2011). For example, the plasma levels of ghrelin, an orexigenic hormone mostly released from the empty stomach, are increased in AN patients along all the day (Germain et al., 2009, 2010). This hormone acts centrally to increase food intake (Wren et al., 2001a,b) and food-motivated behavior (Skibicka et al., 2012), but has also been suggested to be required for the maintenance of blood glucose homeostasis during severe calorie restriction (Zhao et al., 2010). The increases in plasma ghrelin levels in AN seem paradoxical in light of the restrained eating adopted by these patients and suggest an adaptive response to the disease. In regard to the metabolic deficiencies occurring in restrictive AN (see *infra*), the aim of this review is to highlight the impact of ghrelin in the adaptation of the organism to chronic food restriction until it falls into exhaustion and death. A better understanding of the role of this gastric hormone in dysfunctional AN like feeding behavior is important when evaluating its therapeutic potential for the treatment of AN, envisaged to be used alongside mainstay psychiatric and nutritional therapies.

## Physiological Alterations in Anorexia Nervosa

### Types and subtypes of anorexia nervosa: New DSM-V classification

Chronic food restriction is linked to several disorders classified in DSM-V (Diagnostic and Statistical Manuel of Mental Disorders). In the provisional version of DSM-V (of spring 2012; http://www.dsm5.org/meetus/pages/eatingdisorders.aspx), the *Feeding and Eating Disorders* category includes three disorders as manifested by persistent failure to meet appropriate nutritional and/or energy needs and significant weight loss, *AN, avoidant/restrictive food intake disorder (ARFIDO), and atypical AN*.

Diagnostic criteria for AN includes the restriction of energy intake relative to requirements, a drastic significant loss of body weight, an intense fear of gaining weight, body image disturbance, and/or a persistent lack of recognition of the seriousness of the current low body weight. In a recent review (Garcia et al., 2011), the lifetime prevalence of AN was estimated to be 1.9% in female adults to 2.6% in female adolescents in industrialized countries. In the binge eating/purging subtype, the individual engages in recurrent episodes of binge eating or purging behavior while such episodes do not occur in the restricting subtype. Patients from these two subtypes also exhibit differences in eating disorder symptom indicators (Olatunji et al., 2012). However, the subtype determination at the time of the diagnosis should be considered carefully since, over a 7-year period, the majority of women with AN were found to cross over to the restricting and binge eating/purging AN subtypes (Eddy et al., 2008). In a 21-year follow-up study, Löwe et al. (2001) showed that 16% of AN patients deceased due to consequences of their illness: about 50% died because of somatic complications leading to heart attack and the remainder committed suicide. Moreover, mortality is significantly more common among inpatients with somatic comorbidity (like renal, cardiac, bone, and digestive pathologies) than among inpatients without a somatic disease (Erdur et al., 2012). Finally, among the psychiatric comorbidities, AN is often associated with depression, anxiety, obsessive compulsive or personality disorders, and drug abuse (Erdur et al., 2012). Whether AN resembles an addiction behavior disorder remains one major question for physicians and researchers alike. The criteria proposed by Goodman (1990) to identify addictive disorder (Table 2) are found in AN patients. Indeed, Speranza et al. (2012) showed that 35% of the restrictive AN subtype patients, 48% of the binge eating/purging AN subtype patients and 60% of the patients have substance-use disorders and hence, exhibit an addictive disorder according to Goodman’s criteria. From these criteria, an emerging hypothesis of AN implicates neurobiological mechanisms (and hence, investigation strategies for treatments and diagnostic markers) that are based on a reward deficit and on the recognition as an addictive behavior disorder (Alguacil et al., 2011). In fact, among the different theories linking AN and addiction, interestingly, the “auto-addiction opioid model” proposes that this chronic eating disorder could represent an addiction to the body’s endogenous opioids, especially β-endorphins (see Davis and Claridge, 1998). Starvation and excessive exercise, that concern a high percentage of AN patients (Davis et al., 1997; Kohl et al., 2004), are associated with increased levels of β-endorphin, known to further stimulate dopamine in the mesolimbic reward centers (Bergh and Södersten, 1996; Casper, 1998). This mesolimbic pathway plays a pivotal role in addictive behaviors related to drugs and dietary behaviors (Avena and Bocarsly, 2012; Perelló and Zigman, 2012). Importantly, this mesolimbic dopamine pathway is activated by ghrelin (Abizaid et al., 2006; Jerlhag et al., 2006; see *infra*) and, since AN patients have high plasma ghrelin levels (Germain et al., 2009, 2010), it follows that there may be a dysfunctional ghrelin-(dopamine) reward signal in these patients. However, as discussed by Barbarich-Marsteller et al. (2011), there are fundamental differences between AN and addiction. Indeed, the main goal of an individual suffering from a substance abuse disorder is to pursue the immediate effects of the drug on mood and/or behavior (alleviation of anxiety, for example), whereas the goals of an AN patient are both immediate and long term. In these patients, dieting and starvation produce immediate feelings of hunger that may induce a sense of control over one’s body and thereby a sense of control over one’s life while, in the long term, it produces sustained weight loss and thinness that take on an irrationally important value.

**Table 2:** **Addictive disorder criteria according to Goodman (1990)**.

A. Recurrent failure to resist impulses to engage in a specified behavior
B. Increasing sense of tension immediately prior the initiation of behavior
C. Pleasure or relief at the time of engaging in the behavior
D. A feeling of a lack of control while engaging in the behavior
E. At least five of the following:
1. Frequent preoccupation with the behavior or preparatory activities
2. Frequent engaging in the behavior to a greater extent or over a longer period than intended
3. Repeated efforts to reduce, control, or stop the behavior
4. A great deal of time spent in activities necessary for the behavior, engaging in the behavior, or recovering from its effects
5. Frequent engaging in the behavior when expected to fulfill occupational, academic, domestic, or social obligations
6. Important social, occupational, or recreational activities given up or reduced because of the behavior
7. Continuation of the behavior despite knowledge of having a persistent or recurrent social, financial, psychological, or physical problem that is caused or exacerbated by the behavior
8. Tolerance: need to increase the intensity or frequency of the behavior in order to achieve the desired effect or diminished effect with continued behavior of the same intensity
9. Restlessness or irritability if unable to engage in the behavior
F. Some symptoms of the disturbance have persisted for at least 1 month or have occurred repeatedly over a longer period of time

*To reach the categorical diagnosis of addictive disorder according to Goodman (1990), criteria A–D plus criterion E (five among nine symptoms) must be met for at least 1 month*.

In the ARFIDO, insufficient food intake is associated with significant weight loss, nutritional deficiency, dependence on enteral feeding, or nutritional supplements and/or a marked psychosocial dysfunction. In these patients, the eating disturbance does not occur exclusively during the course of AN, and is not associated with body image disturbances. ARFIDO is a new recognized eating disorder previously classified in the left over category *Eating Disorder Not Otherwise Specified* (EDNOS) corresponding to the majority of in-and-out patients treated for eating disorders. Its prevalence remains to be determined.

The last disorder, *atypical AN*, is in the EDNOS category. It includes all the criteria for AN diagnosis except that, despite significant weight loss, the individual’s weight is within or above the normal range. The lifetime prevalence of atypical AN ranges from 2.4% in female adults to 7.7% in female adolescents (Garcia et al., 2011).

Considering this recent classification, in this review we focus on restrictive AN in which the individual is subjected to chronic food restriction that may or may not be associated with intense physical exercise. In fact, the course of AN is extremely variable, with approximately 50–60% of individuals recovering, 20–30% partially recovering, and 10–20% remain chronically ill (Löwe et al., 2001; Fisher, 2003). The unknown etiology of AN renders this complex psychiatric disease difficult to treat and current pharmacological treatments have little efficacy during the acute phase of illness or in preventing relapse (Barbarich-Marsteller, 2007). However, the physiological alterations induced by severe chronic food restriction impact on peripheral compartments (fat, bone, reproductive axis, energy balance) and on central pathways (reward, food intake, mood regulation, etc.) for which the outcome is usually similar whatever the initial cause (personal history, infancy trauma, socio-cultural pressions, personality traits, neurobiological, genetic background, etc.). Some authors even support the view that the physiological mechanisms involved in the regulation of feeding behavior in AN might, in many neurobiological effects, parallel those of obesity. As recently suggested by Jacquemont et al. (2011): “*abnormal eating behaviors, such as hyperphagia and anorexia, could represent opposite pathological manifestations of a common energy balance mechanism, although the precise relationships between these mirror phenotypes remain to be determined*.” Thus, the development of effective/new pharmacological treatments for this disease area would be enhanced if the mechanisms maintaining the abnormal behaviors characteristic of AN are better understood.

### Central and peripheral alterations in anorexia nervosa

Whatever the initial and causal factors leading to AN, all patients display similar energy metabolic deficits and are unable to adapt their feeding behavior to energy demand and costs. In this state, survival requires the development of physiological changes that drive the individual/animal to adapt itself to these drastic conditions. Among all of the variations induced by chronic food restriction, the endocrine, immune, bone, and metabolic changes first allow adaptations to starvation, and are subsequently often directly involved in the complications of the disease (Estour et al., 2010). In addition, some of the feeding-regulatory factors are also involved directly (or not) in the modulation of reward-related and motivational processes, as well as in cognition and emotions associated with the disease. It should be noted that some of these endocrine changes persist after recovery and might contribute to susceptibility for AN recurrence (Lawson and Klibanski, 2008).

Among the biological factors whose levels are altered in AN patients, neurotransmitters and neuropeptides regulating appetite and feeding may contribute to some of the occurring central perturbations (Table 3). Overall, a high degree of heterogeneity has been observed between studies, for most of the assayed factors (plasma and cerebrospinal fluid samples). This heterogeneity could be explained by differences in the clinical characteristics of the samples (as severity and duration of the illness or subtype) and/or the increasing reliability of the methods used to ascertain factor concentrations over the last two decades. Consequentially, it is impossible to link any tendency in changes of levels and/or sensitivity to (an)orexigenic factors to AN, necessitating further investigation.

**Table 3:** **Compared levels of neuropeptides regulating food intake in AN patients and healthy matched population**.

Neuropeptides regulating food intake*	AN/CT	Reference
**Neuropeptide Y**		
CSF NPY	↑	Kaye et al. (1990), Kaye (1996), Baranowska et al. (1997)
Blood NPY	→↓↑	Nedvidkova et al. (2000), Baranowska et al. (2001), Sedlackova et al. (2012)
Blood agouti-related protein	↑	Moriya et al. (2006), Merle et al. (2011)
Blood 26RFa	↑	Galusca et al. (2012)
**Opioid peptides**		
CSF b-endorphins	→↓	Gerner and Sharp (1982a), Baranowska (1990), Kaye (1996)
CSF dynorphins	→	Lesem et al. (1991), Kaye (1996)
Blood b-endorphins	↓↑	Baranowska (1990), Brambilla et al. (1991), Tepper et al. (1992)
**Galanin**		
CSF or plasma galanin	→	Berrettini et al. (1988), Baranowska et al. (1997, 2001)
Blood a-MSH	→	Moriya et al. (2006)
CSF corticotropin-releasing hormone	↑	Gerner and Gwirtsman (1981), Hotta et al. (1986), Kaye et al. (1987), Baranowska (1990)
CSF thyrotropin releasing hormone	↓	Lesem et al. (1994)
CSF neurotensin	→	Nemeroff et al. (1989)
**Somatostatin (SRIF)**		
CSF SRIF	↓→	Gerner and Yamada (1982b), Kaye et al. (1988)
Blood SRIF	↑↓	Pirke et al. (1994), Baranowska et al. (2000), Valevski et al. (2000)
CSF oxytocin	↓	Demitrack et al. (1990)
Blood oxytocin	→↓↑	Chiodera et al. (1991), Lawson et al. (2011a, 2012)
**Brain-derived neurotrophic factor (BDNF)**		
Serum BDNF	↓	Nakazato et al. (2003, 2006, 2009), Monteleone et al. (2004, 2005), Ehrlich et al. (2009), Saito et al. (2009)
Blood BDNF	↑	Mercader et al. (2007)

*Adapted from Monteleone (2011)*.

*Neuropeptides inhibiting food intake are on a gray background.

Systemic hormones directly regulating food intake have been widely studied in AN patients (Table 4). However, some anorexigenic hormones such as leptin decrease while others, such as peptide YY_3–36_ (PYY_3–36_), increase. The same pattern is also observed for the orexigenic hormone, ghrelin (see *infra*). There exists sparse and contradictory data about the anorexigenic factors cholecystokinin (CKK) and glucagon-like peptide 1 (GLP1) in relation to this disease area rendering, it difficult to interpret observed variations in the context of AN. Concerning PYY_3–36_, most studies reported increased levels; although an anorexigenic peptide, this increase is difficult to explain as PYY_3–36_ is normally released in response to food intake. On the contrary, around 50 studies relate a very low leptin blood level in AN patients compared to a healthy matched control population (Table 4). This endogenous signal of energy stores is positively correlated to body mass index. Leptin is considered to be a good predictor of growth hormone (GH) burst, cortisol, estradiol, and thyroid hormone levels and its receptor is widely distributed throughout the body suggesting a pivotal role in mediating the hormonal adaptation to chronic starvation. Furthermore, AN patients display high plasma levels of adiponectin, another adipose-derived circulating cytokine. This anorexigenic hormone plays an important role in energy homeostasis and insulin sensitivity. The high levels of adiponectin in AN might contribute to the higher insulin sensitivity found in these patients. Indeed, insulin levels are usually strongly decreased that could be related to the hypoglycemia observed in AN patients.

**Table 4:** **Compared levels of hormones regulating food intake in AN patients and healthy matched population**.

Hormones regulating food intake*	AN/CT	Reference
**Ghrelin-related**		
Blood total ghrelin	↑	Otto et al. (2001, 2005), Krsek et al. (2003), Nedvidkova et al. (2003), Tanaka et al. (2003a,b,c, 2004), Tolle et al. (2003), Broglio et al. (2004a), Hotta et al. (2004), Misra et al. (2004c, 2005b, 2007, 2008), Soriano-Guillen et al. (2004), Tanaka et al. (2004), Bosy-Westphal et al. (2005), Stock et al. (2005), Troisi et al. (2005), Uehara et al. (2005), Germain et al. (2007, 2009, 2010), Janas-Kozik et al. (2007), Nakahara et al. (2007, 2008), Støving et al. (2007), Lawson et al. (2011b), Sedlackova et al. (2012)
Blood acyl ghrelin (active)	↑	Nakai et al. (2003), Hotta et al. (2004), Uehara et al. (2005), Nakahara et al. (2008), Germain et al. (2009, 2010)
Blood des-acyl ghrelin	↑	Hotta et al. (2004), Nakahara et al. (2008)
Blood obestatin	↑	Nakahara et al. (2008), Germain et al. (2009, 2010), Sedlackova et al. (2012)
Plasma insulin Leptin	(→)↓^1^	Uhe et al. (1992), Tamai et al. (1993), Støving et al. (1999), Gianotti et al. (2000), Gniuli et al. (2001), Delporte et al. (2003), Tanaka et al. (2003a), Weinbrenner et al. (2003), Misra et al. (2004a,b, 2007), Tagami et al. (2004), Bosy-Westphal et al. (2005), Housova et al. (2005), Misra et al. (2005c), Stock et al. (2005), Tomasik et al. (2005), Dolezalova et al. (2007), Dostalova et al. (2007), Kinzig et al. (2007), Nakahara et al. (2007), Støving et al. (2007), Haluzíková et al. (2009), Brick et al. (2010), Fazeli et al. (2010b), Karczewska-Kupczewska et al. (2010, 2012)
**Leptin**		
CSF leptin	↓	Mantzoros et al. (1997), Gendall et al. (1999)
Blood leptin	↓	Ferron et al. (1997), Hebebrand et al. (1997), Mantzoros et al. (1997), Balligand et al. (1998), Gendall et al. (1999), Støving et al. (1999), Monteleone et al. (2000, 2002a,b), Nedvidkova et al. (2000), Di Carlo et al. (2002), Krizova et al. (2002), Delporte et al. (2003), Holtkamp et al. (2003a,b, 2004), Misra et al. (2003, 2004a,b, 2005a,c, 2006, 2007, 2008), Pannacciulli et al. (2003), Tolle et al. (2003), Weinbrenner et al. (2003), Djurovic et al. (2004), Heer et al. (2004), Popovic et al. (2004), Tagami et al. (2004), Dostalova et al. (2005, 2007), Haas et al. (2005), Miljic et al. (2006), Ohwada et al. (2006, 2007), Dolezalova et al. (2007), Germain et al. (2007), Mika et al. (2007), Modan-Moses et al. (2007), Muñoz-Calvo et al. (2007), Nakahara et al. (2007), Haluzíková et al. (2009), Arimura et al. (2010), Estour et al. (2010), Fazeli et al. (2010b), Nogueira et al. (2010), Lawson et al. (2011b), Faje et al. (2012)
Total blood adiponectin	(→↓)↑^2^	Delporte et al. (2003), Iwahashi et al. (2003), Pannacciulli et al. (2003), Misra et al. (2004a), Tagami et al. (2004), Bosy-Westphal et al. (2005), Housova et al. (2005), Dolezalova et al. (2007), Dostalova et al. (2007), Modan-Moses et al. (2007), Nakahara et al. (2007), Støving et al. (2007), Haluzíková et al. (2009), Karczewska-Kupczewska et al. (2010, 2012), Nogueira et al. (2010)
**Cholecystokinin (CKK)**		
CSF or blood CCK	→↓	Phillipp et al. (1991), Geracioti et al. (1992), Tamai et al. (1993), Fujimoto et al. (1997), Tomasik et al. (2004, 2005)
Blood glucagon-like peptide 1	→↓	Tomasik et al. (2002, 2004, 2005)
Blood peptide YY	(→)↑^3^	Stock et al. (2005), Misra et al. (2006, 2007, 2008), Germain et al. (2007), Nakahara et al. (2007), Otto et al. (2007), Lawson et al. (2011b), Sedlackova et al. (2012)

*^1^Most of the studies found decreased insulin levels*.

*^2^Only three studies found no significant differences when compared to control group, and one found a decrease, while all the other found increased adiponectin levels*.

*^3^Most of the studies found increased PYY levels*.

*Hormones inhibiting food intake are on gray background.

More than 90% of adult women with AN are osteopenic, and almost 40% are osteoporotic at one or more sites (Grinspoon et al., 2000). Osteopenia and osteoporosis are frequent consequences of AN, that very often persist after weight gain. Moreover, as synthesized by Confavreux et al. (2009), “*bone can now be considered as a true endocrine organ secreting osteocalcin, a hormone pharmacologically active on glucose and fat metabolism. Indeed osteocalcin stimulates insulin secretion and β-cell proliferation. Simultaneously, osteocalcin acts on adipocytes to induce adiponectin, which secondarily reduce insulin resistance*.” For these reasons, studies comparing bone turnover markers in AN patients with healthy control are presented in Table 5. Anorectic patients display increased levels of bone resorption markers and decreased bone formation markers. We may conclude that the bone mass alteration in patients with AN is dual: an increase of resorption and a decrease of bone formation. Moreover, the decrease in osteocalcin level could also contribute to the hypoinsulinemia and hypoadiponectinemia usually described.

**Table 5:** **Compared levels of bone turnover markers in AN patients and healthy matched population**.

Bone turnover markers*	AN/CT	Reference
Blood OC	(→)↓^1^	Calero et al. (1999), Grinspoon et al. (1999), Caillot-Augusseau et al. (2000), Gordon et al. (2002), Misra et al. (2003, 2004b, 2006, 2007, 2008), Weinbrenner et al. (2003), Galusca et al. (2006), Legroux-Gérot et al. (2007), Ohwada et al. (2007), Viapiana et al. (2007), Estour et al. (2010), Ostrowska et al. (2010, 2012a,b)
Blood procollagen type 1 N-terminal propeptide (PINP)	→↓	Calero et al. (1999), Faje et al. (2012)
Blood procollagen type 1 C-terminal propeptide (PICP)	↓	Misra et al. (2003, 2004b, 2006, 2007), Heer et al. (2004), Mika et al. (2007)
Blood bone specific alkaline phosphatase (BSAP)	(→)↓^2^	Calero et al. (1999), Gordon et al. (2002), Misra et al. (2003, 2006), Heer et al. (2004), Bolton et al. (2005), Galusca et al. (2006), Legroux-Gérot et al. (2007), Mika et al. (2007), Ohwada et al. (2007), Viapiana et al. (2007)
Blood or urinary c-terminal cross-linking telopeptide of type 1 collagen (CTX)	↑↓^3^	Caillot-Augusseau et al. (2000), Weinbrenner et al. (2003), Galusca et al. (2006), Mika et al. (2007), Estour et al. (2010), Ostrowska et al. (2010, 2012a,b), Faje et al. (2012)
Blood cross-linked N-telopeptides of type 1 collagen (NTX)	↑↓	Gordon et al. (2002), Dominguez et al. (2007)
Urinary NTX/creatinine	↑↓^4^	Grinspoon et al. (1999), Misra et al. (2003, 2006, 2007, 2008), Dominguez et al. (2007)

*^1^All but three studies found significant or non-significant decreased OC levels*.

^2^All but three studies found significant or non-significant decreased BSAP levels

*^3^Increased CTX found by Caillot-Augusseau et al. (2000), Weinbrenner et al. (2003), Galusca et al. (2006), Ohwada et al. (2007), and Estour et al. (2010)*.

*^4^Only Grinspoon found increased urinary NTX/creatinine levels*.

*Bone resorption markers are on gray background.

A number of other endocrine disturbances have also been described in AN patients (Table 6). Hypothalamic-pituitary-adrenal axis deregulation is commonly suggested in AN. Indeed, AN is characterized by hypercortisolemia (Table 6) and, as mentioned by Miller (2011): “*overnight blood cortisol levels are inversely associated with bone mineral density and positively associated with severity of depression and anxiety symptoms in women with anorexia nervosa (Lawson et al., 2009). Therefore, hypercortisolemia may also contribute to the severe bone loss incurred and the highly prevalent psychiatric comorbidities in women with anorexia nervosa*.” Hypothalamic amenorrhea is another characteristic feature of AN, and has been attributed to a state of severe energy deficit from restricted energy intake, increased energy expenditure or both. Women and adolescent girls with AN have lower levels of estradiol, luteinizing hormone (LH), and for some of them follicle stimulating hormone (FSH; Table 6). The low levels of insulin-like growth factor-1 (IGF-1) and insulin may also contribute to this hypogonadal state and impact on bone turnover. The GH/IGF-1 axis is also altered in most of the studies (Table 6). Indeed, AN is associated with a nutritionally acquired hepatic resistance to GH with decreased production of IGF-1 and increased GH levels. Such an increase is due to a reduced feedback at the level of the pituitary and hypothalamus from low IGF-1 levels, and high levels of ghrelin (Table 4, see *infra*). Most studies report low levels of T3 and/or T4 thyroid hormones in patients with AN (Table 6). T3 and T4 plasma levels are enhanced by leptin administration in women with AN, and the levels of these three hormones are positively associated (Haas et al., 2005; Misra et al., 2005a). Moreover, ghrelin is known to inhibit the release of pituitary thyroid stimulating hormone (Wren et al., 2000), and studies indicate negative correlations between ghrelin and thyroid hormones plasma levels in AN (Misra et al., 2005c). These data suggest that low leptin and high ghrelin levels may contribute to lower thyroid hormone levels in AN. Finally, inflammatory cytokines were assayed in AN patients and matched healthy controls (Corcos et al., 2003). Data did not show significant variations suggesting that AN might not have an inflammatory component.

**Table 6:** **Compared levels of other factors altered in AN patients**.

Other factors	AN/CT	Reference
**Growth factors**		
Blood GH	(→)↑^1^	Gianotti et al. (2000), Misra et al. (2003, 2004a,c, 2005c, 2006, 2007), Støving et al. (2003), Tolle et al. (2003), Broglio et al. (2004a), Tanaka et al. (2004), Miljic et al. (2006), Germain et al. (2007), Polli et al. (2008), Arimura et al. (2010), Estour et al. (2010)
Blood IGF-1	↓	Grinspoon et al. (1999), Støving et al. (1999, 2003, 2007), Gianotti et al. (2000), Nedvidkova et al. (2000), Di Carlo et al. (2002), Gordon et al. (2002), Misra et al. (2003, 2004a, 2005c, 2006, 2007, 2008), Tolle et al. (2003), Broglio et al. (2004a), Heer et al. (2004), Ohwada et al. (2006, 2007), Germain et al. (2007), Legroux-Gérot et al. (2007), Mika et al. (2007), Polli et al. (2008), Haas et al. (2009), Arimura et al. (2010), Brick et al. (2010), Estour et al. (2010), Fazeli et al. (2010b), Faje et al. (2012)
**Steroid and sexual hormones**		
Blood LH	↓	Støving et al. (1999, 2007), Di Carlo et al. (2002), Holtkamp et al. (2003b), Popovic et al. (2004), Misra et al. (2005c), Dominguez et al. (2007), Germain et al. (2007), Oswiecimska et al. (2007), Tomova et al. (2007), Nogal et al. (2008), Arimura et al. (2010), Estour et al. (2010), Ziora et al. (2011)
Blood FSH	(→)↓^2^	Støving et al. (1999, 2007), Di Carlo et al. (2002), Holtkamp et al. (2003b), Popovic et al. (2004), Dominguez et al. (2007), Germain et al. (2007), Oswiecimska et al. (2007), Tomova et al. (2007), Nogal et al. (2008), Arimura et al. (2010), Estour et al. (2010), Ziora et al. (2011)
Estrogens	(→)↑^3^	Grinspoon et al. (1999), Støving et al. (1999, 2007), Monteleone et al. (2000, 2001), Di Carlo et al. (2002), Holtkamp et al. (2003b), Misra et al. (2003, 2004a,b, 2005c, 2006, 2007, 2008), Tolle et al. (2003), Heer et al. (2004), Popovic et al. (2004), Bolton et al. (2005), Dominguez et al. (2007), Germain et al. (2007), Mika et al. (2007), Ohwada et al. (2007), Oswiecimska et al. (2007), Haas et al. (2009), Arimura et al. (2010), Brick et al. (2010), Estour et al. (2010), Buehren et al. (2011), Ziora et al. (2011), Faje et al. (2012)
Cortisol	(→)↑^4^	Grinspoon et al. (1999), Støving et al. (1999), Monteleone et al. (2000, 2001), Putignano et al. (2001), Misra et al. (2003, 2004b, 2005c, 2006, 2007, 2008), Tolle et al. (2003), Weinbrenner et al. (2003), Heer et al. (2004), Troisi et al. (2005), Miljic et al. (2006), Germain et al. (2007), Oswiecimska et al. (2007), Nogal et al. (2008), Haas et al. (2009), Arimura et al. (2010), Estour et al. (2010), Buehren et al. (2011), Ziora et al. (2011), Faje et al. (2012)
Thyroid hormones	(→)↓^5^	Nedvidkova et al. (2000), Di Carlo et al. (2002), Holtkamp et al. (2003b), Weinbrenner et al. (2003), Onur et al. (2005), Troisi et al. (2005), Brambilla et al. (2006), Ohwada et al. (2006, 2007), Oswiecimska et al. (2007), Nogal et al. (2008), Arimura et al. (2010), Estour et al. (2010), Buehren et al. (2011), Ziora et al. (2011)

*^1^All but two studies found increased GH levels*.

*^2^All but one study found decreased FSH*.

*^3^Most of the studies found decreased estrogen levels*.

*^4^Most of the studies found increased Cortisol levels*.

*^5^All but two studies found decreased T3 and/or T4 levels*.

### Animal models of chronic food restriction: A way to decipher the physiological mechanisms of anorexia nervosa

The use of appropriate animal models mimicking most of the physiological changes occurring in AN might help to determine more precisely the potential mechanisms, central and/or peripheral, involved in the early adaptive state that precedes exhaustion of the individual/animal. However, developing and using animal models of psychiatric disorders is inherently difficult due to the complex nature of these illnesses. In the literature, numerous models of genetic deficient mice for one or multiple genes involved in the regulation of feeding behavior/reward/energy balance have been developed (for review, see Siegfried et al., 2003; Kim, 2012). These genetic models give essential mechanistic data related to one specific pathway but do not completely mirror the symptoms observed in human disease (Willner, 1984; Smith, 1989). Indeed, the use of more “environmental models” that mimic most of the physiological symptoms of AN are preferred as they provide insight regarding how the disease might progress toward exhaustion. Initially, the most widely used animal model, whatever the species, is the chronic food restriction model (for review, see Kim, 2012). However, it does not take into account several conditions observed in AN patients that are self-starvation, hyperactivity, and chronic stress. The rat model of self-starvation developed by Routtenberg and Kuznesof (1967) consists in housing one rat in a cage equipped with a running wheel and submitting the animal to a food restriction (1 h-feeding per day). This model later coined the term “the activity-based anorexia (ABA) model.” It produces a rapid decrease in body weight and food intake, hyperactivity, hypothermia, loss of estrus, and an increase in HPA axis activity (Hall and Hanford, 1954; Routtenberg and Kuznesof, 1967; Burden et al., 1993). Moreover, in this model, the rats eat less than inactive rats fed with the same schedule, and can even starve themselves to death. In the ABA model, the long term exposure (few days) to low leptin and high ghrelin levels, induced a tissue-specific expression pattern of ghrelin and leptin receptors (Pardo et al., 2010). Furthermore, Verhagen et al. (2011) found that plasma ghrelin levels are highly associated with food anticipatory behavior, measured by running wheel activity in rats. This effect is dependent of the central ghrelin signaling system *via* growth hormone secretagogue receptor 1a (GHS-R1A). In many aspects this model mimics numerous physiological alterations observed in AN. However, as specified by Klenotich and Dulawa (2012), the ABA paradigm is strongly dependent upon factors that amplify or reduce some parts of the phenotype, i.e., the choice of rodent strain (more or less resistant to ABA, Gelegen et al., 2007), the sex of animal (female are more vulnerable to ABA), the age (younger animals are more susceptible to ABA), the temperature (increasing the temperature to 32°C strongly reduces the ABA behavior, Cerrato et al., 2012), the time of the day the animals receive food, and the hydration/dryness of the food. In fact, Boakes and Juraskova, 2001; Boakes, 2007) demonstrated that the “self-starvation” observed in ABA rats might reflect both the reduced palatability of the dry chow for a dehydrated animal and satiety signals from a stomach full of water. Thus, giving hydrated food during the 1 h-feeding schedule completely abolishes the ABA phenotype (rapid weight loss, hyperactivity, etc.). Currently, we are developing an adaptation of the ABA model in female mice that aims to follow the long term (more than 2 weeks) physiological alterations induced by a combination of physical activity and 50% food restriction. Our recent results, involving a 2-week protocol, indicate that our selected ABA model induces a rapid and stable loss of weight, changes the circadian locomotor activity, alters energy balance (corresponding to the passage from phase I to II; Table 1), induces hypoglycemia, hypoleptinemia, hyperghrelinemia, and a central alteration in the hypothalamic feeding centers (Méquinion, personal data). Thus, the combination of exercise (in running wheels) and food deprivation following a certain schedule might be considered as two important chronic stress factors that could reinforce the weight loss by modifying feeding behavior, as observed in our study. Other models based on chronic stress that are associated with food deprivation use tail pinching, cold swimming (Shimizu et al., 1989; Wong et al., 1993), or separation. We choose this last model, named “Separation-Based Anorexia” (SBA) to study the impact of chronic stress associated with caloric insufficiency. In our protocol, 8-week old female mice are separated and fed with a time-restricted food access for up to 10 weeks. Our recent results showed a 20–25% body weight loss with a cumulative food intake just under that of the control group. Moreover, SBA mice displayed reduced lean, fat, and bone masses associated with hypoleptinemia and alteration in GH/IGF-1 axis. Finally, an alteration of the estrous cycle was also observed (Zgheib, personal data).

To date, these described “environmental animal models” (ABA and SBA) remain the only models that enable long term studies of how chronic food restriction impacts upon physiology at different levels (energy balance, reproduction, bone/fat regulation, etc.), and of the mechanisms responsible for the sustenance of these alterations on different tissues often not available on patients (brain, bones, fat, muscle, liver, etc.). They will also facilitate determination of whether the dramatic outcome of the patients might be related to a specific dysregulation of one or many biological factors that can be considered as a marker of the disease and potentially also in its evolution. In addition, ABA and SBA models exhibit good face validity for most of the physiological symptoms of AN.

## Ghrelin a Key Energy Balance Hormone: Role in Anorexia Nervosa

### Origin and biosynthesis of ghrelin

**Figure 1:**
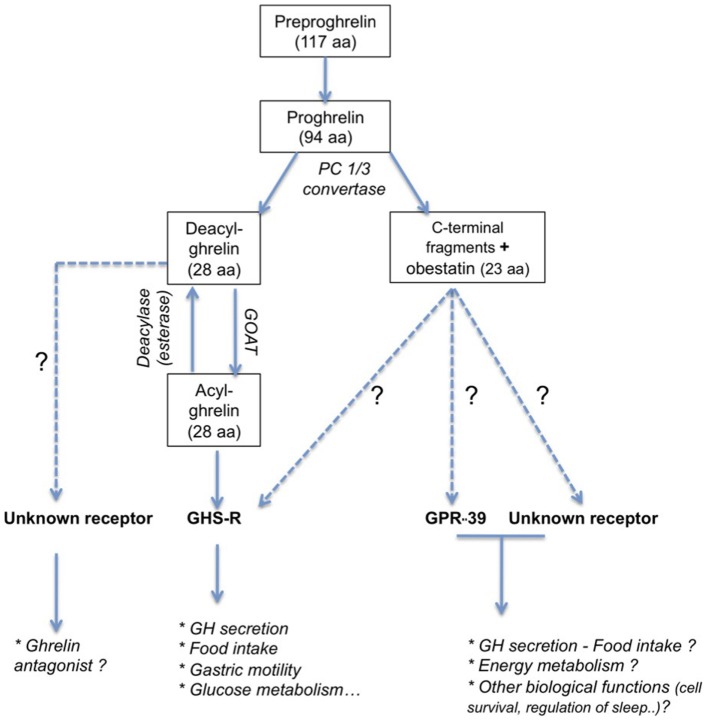
**Principal peptide products obtained by post-translational processing of preproghrelin peptide**. Ghrelin and obestatin act on receptors belonging to the GPCR family. Even if the exact role of obestatin remains question of debate (see Hassouna et al., 2010), ghrelin has been first described to be a GH-secretagogue. Beside an obvious role in the regulation of food intake, ghrelin is also implicated in numerous biological function (see Veldhuis and Bowers, 2010). The active form of ghrelin, acyl ghrelin, is obtained by octanoylation of deacyl ghrelin. Its receptor is not yet identified and its function is currently unclear even if some evidences support an antagonistic effect to ghrelin (see Delhanty et al., 2012).

Ghrelin is a 28 amino acid, initially isolated from rat stomach (Kojima et al., 1999). The preproghrelin messenger RNA (mRNA) is mainly expressed in the X/A-like oxyntic gland cells of the gastric fundus mucosa equivalent to P/D1 cells in humans (Bordi et al., 2000). Ghrelin is also produced in other parts of the gastrointestinal tract, and it is expressed at lower levels in pancreas, kidney, testis, placenta, and bone (Gnanapavan et al., 2002; González et al., 2008) and hypothalamic neurons (Cowley et al., 2003). The 117 amino acid preproghrelin is processed by cleavage and results in two peptides (Figure 1): obestatin and proghrelin (Jeffery et al., 2005). Des-acyl ghrelin is then cleaved from the 94 amino acid proghrelin precursor by enzymes like the prohormone convertase 1/3 (Zhu et al., 2006). This 28 amino acid peptide is modified post-translationally in the active acylated form of ghrelin, capable to bind to its receptor, the GHS-R1a. The octanoylation at the third N-terminal amino acid, usually serine (Kojima et al., 1999), is catalyzed the enzyme ghrelin octanoyl-acyltransferase (GOAT, Yang et al., 2008), which is expressed predominantly in the stomach, gut, and pancreas, but also at other sites (Kang et al., 2012). Ghrelin concentrations in blood comprise principally des-acyl ghrelin (85–90%) and in lesser amounts acyl ghrelin (10–15%) and C-terminal proghrelin peptides (Pemberton and Richards, 2007).

### Ghrelin receptor and distribution

Ghrelin is the only known ligand to bind to GHS-R1a (Howard et al., 1996; Gutierrez et al., 2008). This receptor belongs to the G-protein coupled receptor family (GPCR; Holst and Schwartz, 2006) and has two variants, GHS-R1a and GHS-R1b, which are splice variants of the same gene. Type 1a is the full length, seven-transmembrane domain receptor, and the type 1b isoform is a C-terminally truncated, five-transmembrane domain variant (Kojima et al., 1999). Only the GHS-R1a is fully functional, binding mostly with acylated ghrelin on Gα_q_-protein, whereas the 1b isoform is thought to be physiologically inactive. It should be noted that des-acyl ghrelin does not compete with acyl ghrelin for GHS-R1a to any significant extent. Indeed, supraphysiological concentrations of des-acyl ghrelin are necessary to allow binding and activation of GHS-R1a (Veldhuis and Bowers, 2010; Delhanty et al., 2012). Although derived from the same precursor, obestatin is a cognate ligand for the orphan receptor GPR39, another member of the ghrelin receptor subfamily (McKee et al., 1997; Holst et al., 2004).

Growth hormone secretagogue receptor 1a is abundantly expressed within the CNS. Notably, a large population of GHS-R1a-expressing neurons are located in the hypothalamic arcuate nucleus (ARC), which has a crucial role in energy balance control. Other hypothalamic areas expressing this receptor of relevance for feeding control include the ventromedial hypothalamus (VMH), paraventricular nucleus (PVN), anteroventral preoptic nucleus, anterior hypothalamic area, lateral hypothalamic area (LHA), suprachiasmatic nucleus, supraoptic nucleus, and the tuberomammillary nuclei (Guan et al., 1997; Gnanapavan et al., 2002; Camiña, 2006; Harrold et al., 2008). Moreover, mRNA studies also demonstrate the presence of this receptor in limbic and mesolimbic structures known to be involved in motor control, emotional reactivity and reward/motivation systems such as the hippocampus (dentate gyrus, CA2, and CA3 regions), pars compacta of the substantia nigra, ventral tegmental area (VTA), raphe nuclei, laterodorsal tegmental nucleus (Guan et al., 1997; Zigman et al., 2006), and amygdala (Alvarez-Crespo et al., 2012). In the periphery, GHS-R1a is expressed in different tissues and organs implicated in energy balance, e.g., in the anterior lobe of the pituitary on somatotroph cells (Briggs and Andrews, 2011), pancreas, spleen, stomach, intestine, heart, thyroid, gonads, adrenal, liver, skeletal muscle, and adipose tissues (Papotti et al., 2000). The truncated form is also found in various tissues but its exact role is not well known (Gnanapavan et al., 2002; Camiña, 2006). Interestingly, GHS-R1a is constitutively active even when unstimulated by the afferent ghrelin signal (Petersen et al., 2009). Finally, ghrelin receptors are able to interact with other receptors to form homo- or hetero-dimers, like GHS-R1a/GHS-R1a and GHS-R1a/GHS-R1b (Chow et al., 2012). Co-expression of the truncated variant of ghrelin receptor with the full length variant attenuated the constitutive signaling probably because the translocation of the ghrelin receptor from the plasma membrane to the cell nucleus is decreased (Mokrosinski and Holst, 2010). GHS-R1a also appears to heterodimerize with other GPCRs, at least in *in vitro* test systems, and can explain the differential ghrelin signaling (review in Schellekens et al., 2010). Heterodimerization of GHS-R1A with the dopamine receptor D2 has recently been shown to occur in mouse hypothalamic neurons that regulate appetite (Kern et al., 2012) Since GHS-R1a acts as an allosteric modulator of D1 and D2 signaling, this finding implies a functional role for the expressed GHS-R1a in brain areas that may be less accessible to peripherally produced ghrelin and where there is no local production of ghrelin (Jiang et al., 2006; Kern et al., 2012).

### Roles of ghrelin

In line with the broad expression of ghrelin and its receptor, this hormone is involved in multiple biological functions, many of which are linked to feeding control. Initially, this gut-brain signal was shown to have direct pituitary GH-releasing effects, reproducing the known effects of the so-called growth hormone secretagogues (GHS). This term refers a group of synthetic GHS-R1a ligands, the first group of which was derived from metenkephalin (described by Bowers et al., 1977) and included the hexapeptide GHRP-6 (Bowers et al., 1984) that is now recognized as a synthetic ghrelin mimetic. Both ghrelin and its receptor have been strongly conserved during evolution, supporting the notion that GHS-R1a and its natural ligand play a fundamentally important role in biology (Palyha et al., 2000).

Ghrelin’s most characterized effects are: (i) its ability to stimulate GH secretion, likely of relevance for glucose homeostasis and energy balance; (ii) its role as an orexigenic hormone acting at key hypothalamic and midbrain circuits involved in feeding control; (iii) its involvement in various other physiological functions like gastrointestinal, cardiovascular, pulmonary and immune function, sleep duration, learning, memory, and behavior, cellular proliferation, immunomodulation, reproduction, and bone physiology. Most of these physiological functions are altered in AN indicating a potential role for ghrelin in the pathogenesis of this disease.

#### Role of ghrelin in the regulation of appetite, food intake, and energy balance

Ghrelin acts at different levels to stimulate GH secretion and thus modulate hepatic IGF-1 production (Peino et al., 2000). GHS-R1a is expressed by GHRH arcuate neurons but also by GH cells in the anterior pituitary gland (Kojima et al., 1999). Ghrelin and GHS activate ARC cells (Dickson et al., 1993; Hewson and Dickson, 2000) including neuroendocrine cells in this region (Dickson et al., 1996), notably a sub-population of GHRH neurons (Dickson and Luckman, 1997; Osterstock et al., 2010). Indeed, these compounds acts in synergy with GHRH to induce a greater GH release than would be induced by GHRH alone (Bowers et al., 1984; Arvat et al., 2001; Hataya et al., 2001). This ghrelin-stimulated GH release is dose-dependent and could explain why AN patients have elevated circulating GH levels (Kojima and Kangawa, 2005; Miljic et al., 2006; Misra et al., 2006; Germain et al., 2007, 2010; Estour et al., 2010). Chronic starvation is associated with GH resistance and relatively low IGF-1 levels involving a feedback mechanism, rather than body composition parameters or other circulating factors, e.g., free fatty acid or insulin levels (Støving et al., 1999; Brick et al., 2010). Besides its role in growth, GH stimulates lipolysis through a mechanism independent of IGF-1 (Fazeli et al., 2010a), for which the effects are largely anabolic. The increased GH secretion and the reduction of IGF-1 in starvation may be adaptive since they respectively serve the function of mobilizing fat stores in the setting of reduced energy availability and reduce anabolism. However, the reduction of IGF-1 levels may also have deleterious effects, contributing to bone, and muscle loss in AN women. Even if the mechanisms underlying the development of GH resistance in states of chronic undernutrition are not as well established, ghrelin might strongly participate to such endocrine dysregulation. Studies conducted in rodents support a close link between ghrelin signaling and altered GH/IGF-1 status. In rodents, fasting induced higher expression of GHS-R1a and overexpression of GHS-R1a in female mice provoked higher expression of GH and GHRH (Veldhuis and Bowers, 2010). Furthermore, suppressed ghrelin signaling (using antisense RNA knockdown against GHS-R1a or a GHS-R1a antagonist, BIM-28163), caused a decrease in GH peak pulsatility with or without a decrease in plasma levels of IGF-1 (Zizzari et al., 2005; Veldhuis and Bowers, 2010). Finally, plasma ghrelin concentration is negatively correlated with body weight and subcutaneous, visceral, and total adiposity (reviewed in Veldhuis and Bowers, 2010), probably due to a long term effect on ghrelin in driving pulsatile GH secretion, which is strongly lipolytic.

Ghrelin is perhaps best known as a circulating hunger signal necessary for meal initiation and meal anticipation with a secretion occurring in a pulsatile manner starting with a preprandial rise and postprandial fall 1 h after food intake (Ariyasu et al., 2001; Cummings et al., 2001; Tschöp et al., 2001; Zizzari et al., 2011; Merkestein et al., 2012). Moreover, whatever the route of injection, ghrelin increases food intake both in humans and animals (Wren et al., 2001a,b). In addition, prolonged food reduction or severe caloric restriction causes an increase in plasma ghrelin concentration (Wren et al., 2001b; Méquinion, personal data). In AN patients, ghrelin levels are increased up to twofold and return to normal levels after weight restoration (Otto et al., 2001, 2005; Tolle et al., 2003; Germain et al., 2009; Yi et al., 2011). However, it appears that fluctuations in ghrelin are not always influenced by food intake in AN (Germain et al., 2009) suggesting impairment in its regulation, probably due to a chronic adaptation to long term food restriction (Yi et al., 2011). Mice lacking the gene for ghrelin or its receptor have normal food intake when fed chow, probably due to compensatory adaptation during embryonic development, but show a degree of protection from obesity when fed a high-fat diet, especially from an early age (Sun et al., 2003, 2004; Wortley et al., 2004, 2005).

Ghrelin also appears to be of importance in the regulation of lipid and glucose metabolism. It has been attributed a role in the maintenance of normal blood glucose levels (Grove and Cowley, 2005). There are indications that ghrelin’s effects on the GH axis may have relevance for glucose homeostasis (and even survival) during chronic food deprivation. Mice lacking acyl ghrelin (due to knockout of GOAT) lose glycemic control and become moribund by 1 week of 60% food deprivation, an effect that can be circumvented by infusion of either acyl ghrelin or GH throughout this 1-week period (Zhao et al., 2010). The work of Sun et al. (2008) on ghrelin homeostasis in ghrelin KO and GHS-R KO mice demonstrate that the ghrelin/GHS-R pathway appears to play an important role in glucose homeostasis by regulating insulin sensitivity and glucose sensing, particularly under conditions of negative energy balance. However, data indicating an action of ghrelin on plasma insulin levels are still controversial (Castañeda et al., 2010; Sangiao-Alvarellos and Cordido, 2010). Intravenous ghrelin injection leads to a decrease in plasma insulin and an increase in blood glucose (Broglio et al., 2001). This result is not found universally and it may be related to the physiological vs. pharmacological doses used (Castañeda et al., 2010). Ghrelin could potentially decrease insulin secretion by altering insulin sensitivity (Castañeda et al., 2010). This is in agreement with the results obtained during insulin and glucose tolerance tests performed in AN patients (Broglio et al., 2004a; Harada et al., 2008). Moreover, under chronic food restriction, fatty acids are mobilized and their oxidation could increase the production of octanoic acid, thereafter used to octanoylate des-acyl ghrelin, leading to a global increase of plasma ghrelin levels. Thus, the greater levels of glucose observed in ABA mice (Méquinion, personal data) or wild type mice during the early phases of the chronic food restriction might be driven by this increase in ghrelin. This might contribute to the adaptive state in the first stages of AN, before a depletion of the supply of free fatty acids and induction of ketosis. Ghrelin acts directly on the liver, to favor glycogenolysis but also on muscle and adipose tissues. Indeed, subcutaneous injection of ghrelin in rats induces an increase of hepatic triglycerides associated with an increase in the gene expression of enzymes involved in lipogenesis like acetyl-CoA carboxylase (ACC) and fatty acid synthase (FAS, Barazzoni et al., 2005) as well as increased expression of ACC and FAS mRNA in white visceral adipose tissue (Thompson et al., 2004; Barazzoni et al., 2005). By contrast, subcutaneous ghrelin injection induces a decrease of triglyceride content in muscle without modifying ACC expression and AMPK phosphorylation (Barazzoni et al., 2005). These effects are especially observed in the gastrocnemius muscle, a fast-twitch muscle that is predominantly glycolytic. Similar variations are observed in the food-restricted condition (Samec et al., 2002). Finally, Pardo et al. (2010) describe, in the ABA model, a tissue-specific expression pattern of GHS-R1a receptors in visceral and subcutaneous fat and within the muscle. Indeed, the oxidative-soleus type of muscle appears to be more susceptible to circulating ghrelin levels than the glycolytic-gastrocnemius type under exercise and food restriction situations. All of these modifications could provide a defense mechanism to maintain energy homeostasis in the unbalanced energy state that is found in AN patients.

#### The central orexigenic effect of ghrelin

**Figure 2:**
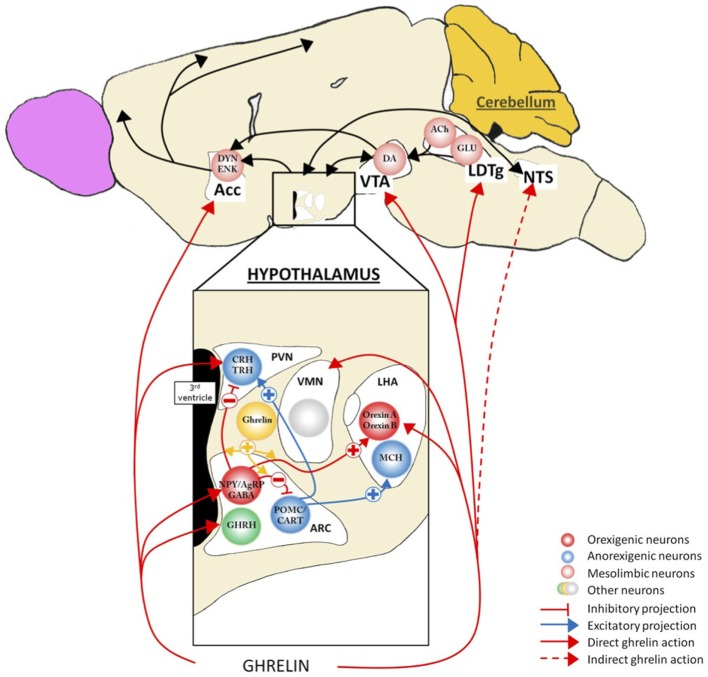
**Action of ghrelin in the brain**. Ghrelin acts at different levels of the brain to stimulate food intake *via* hypothalamus and meso-cortico-limbic pathway. In the hypothalamus, ghrelin activates orexigenic neurons (AgRP/NPY), which inhibit anorexigenic neurons (POMC/CART) *via* GABA projections. They are connected to second order neurons like CRH and TRH neurons located in the PVN and/or the orexin neurons found in the LHA. POMC/CART neurons activate MCH neurons. Ghrelin acts also at different levels of meso-cortico-limbic pathway: LDTg, VTA, and Acc. Ghrelin acts directly on VTA to stimulate dopamine release in Acc. dopamine release is controlled by cholinergic LDTg neurons. Ghrelin could also act on NTS to stimulate the food intake *via* either vagal nerve or area postrema, see Figure 4. Acc, accumbens nucleus; ACh, acétylcholine; AgRP, agouti-related peptide; ARC, arcuate nucleus; CART, cocaine- and amphetamine-regulated transcript; CRH, corticotropin-releasing hormone; DA, dopamine; DYN, dynorphin; ENK, enkephalin; GABA, γ-aminobutyric acid; GHRH growth-hormone-releasing hormone; GLU, glutamate; LDTg, laterodorsal tegmental area; LHA, lateral hypothalamic area; MCH, melanin-concentrating hormone; NPY, neuropeptide Y; NTS, nucleus tractus solitarius; POMC, pro-opiomelanocortin; PVN, paraventricular nucleus; TRH, thyrotropin releasing hormone; VMH, ventromedial nucleus; VTA, ventral tegmental area.

Appetite, food intake, and energy balance are finely tuned by a complex intercommunication between neural networks and peripheral tissues (Figure 2). Within the CNS, various hypothalamic nuclei containing orexigenic and anorexigenic neurons regulate the different facets of food intake. The ARC cells targeted by ghrelin and its mimetics include the orexigenic neuropeptide Y (NPY) cells (Dickson and Luckman, 1997) that co-express another orexigenic peptide, agouti-related peptide (AgRP). Following administration of ghrelin, these neurons are activated, reflected by the induction of Fos-protein in discrete cell groups (Hewson and Dickson, 2000; Wang et al., 2002), by increased action potential firing (Cowley et al., 2003; Andrews et al., 2008) and by an increased expression of NPY and AgRP mRNA (Kamegai et al., 2000, 2001; Nakazato et al., 2001). Furthermore, the stimulatory effects of ghrelin on NPY/AgRP neurons are complemented by a reduction of the ARC anorexigenic pro-opiomelanocortin (POMC) neuronal activity *via* inhibitory GABA-ergic inputs from NPY/AgRP neurons (Cowley et al., 2003). Interestingly, sensitivity of the ARC cells to ghrelin appears to be nutritionally regulated as the Fos response was increased up to threefold in fasting rats relative to fed animals (Hewson and Dickson, 2000) an effect that was reversed once again upon refeeding (Luckman et al., 1999). Collectively, these data indicate that ghrelin activates a key orexigenic pathway in the hypothalamic ARC, the NPY/AgRP cells and that this response is metabolically regulated. Consistent with this, stimulation of hypothalamic GHS-R1a results in an anabolic response characterized by an increase in food intake (Wren et al., 2000) and a decrease in energy utilization (Tschöp et al., 2000). The feeding effects ghrelin appear to require normal NPY/AgRP signaling since ablation of NPY or AgRP neurons or the use of NPY receptor antagonists abolish these effects (Chen et al., 2004; Luquet et al., 2007). Conditional deletion of NPY/AgRP co-expressing neurons in the ARC of adult mice (by targeting the human diphtheria toxin to the AgRP locus) caused a rapid starvation to death (Luquet et al., 2005). Moreover, mice homozygous for the anorexia (*anx*) mutation, characterized by poor food intake and death by 3–5 weeks after birth (Maltais et al., 1984) display a lower density of hypothalamic neuropeptides. The data of Nilsson et al. (2011) support the hypothesis of degeneration of hypothalamic ARC neuron populations; the AgRP system appears to be the first system affected and the POMC system being secondary in this process. Finally, in our models of chronic food restriction (SBA and ABA), we found an alteration of the AgRP signal with an accumulation of this peptide in ARC neurons (Méquinion and Nilsson, personal data). Thus, there are numerous lines of evidence supporting the fact that in chronic food restriction (and probably in AN), a dysregulation of the AgRP system occurs that contributes to deficient ghrelin signaling at the level of the ARC. Recent neuroimaging data obtained from AN patients differing in disease duration showed a significant reduction of total white matter volume and focal gray matter atrophy in various brain areas such as the hypothalamus, especially in patients with shorter food restriction. Collectively, these studies highlight the potential role of endocrine and central (hypothalamic) dysfunction in the altered homeostatic metabolic status in AN, as described in animal models (Boghi et al., 2011).

At the hypothalamic level, ghrelin has also been reported to act directly or indirectly on other nuclei linked to feeding control such as the VMH, PVN, and LHA (López et al., 2008; Mano-Otagiri et al., 2009; Lamont et al., 2012). Although not coupled to c-fos expression, VMN cells exhibit a robust electrical response following bath application of a ghrelin agonist (Hewson et al., 1999). The elegant study of López et al. (2008) showed that, in fasted rats, an elevated ghrelin tone was associated with an increased activation of hypothalamic AMPK and a decreased mRNA expression of enzymes (like FAS) involved in the *de novo* fatty acid biosynthesis only in the VMH. They concluded that the energy peripheral signals sensed to regulate fatty acid metabolism in the hypothalamus and consequently the feeding behavior, may not be a nutrient, but ghrelin through an action at the level of the VMH.

Ghrelin’s effects in the PVN are also likely linked to feeding control as direct intra-PVN injection of ghrelin induces a robust feeding response that is coupled to neuronal (c-fos) activation (Olszewski et al., 2003). Consistent with this, a reduction of GHS-R1a gene in the PVN using RNA interference in rats, significantly reduced body weight and blood ghrelin levels without affecting food intake (Shrestha et al., 2009). These data reflect a role for ghrelin in the modulation of PVN neuron activity in that is linked to energy homeostasis, but the mechanisms of action remains to be elucidated.

In the LHA, ghrelin is thought to mediate hyperphagia through orexin neurons. Indeed, central administration of ghrelin or a ghrelin mimetic induces Fos expression in orexin-containing, but not melanin-concentrating hormone-containing, neurons (Lawrence et al., 2002; Olszewski et al., 2003), and activates glucose responding neurons (Chen et al., 2005a) in this area. Furthermore, ghrelin-induced feeding is suppressed in orexin KO mice (Toshinai et al., 2003). The role of orexin neurons to simulate feeding behavior (appetite/metabolism) is now well established although they are also especially important for sleep and wakefulness (España and Scammell, 2011; Gao, 2012) and play important roles in the stress response, in analgesia and reward/addiction (see Kukkonen, 2013). Moreover, ghrelin’s effects to increase the reward value of a high-fat diet appear to involve a LHA-VTA orexin pathway (Perello et al., 2010).

The action of ghrelin to increase food intake and associated appetitive behaviors involves an integrated neurobiological response exerted at many levels, not only via the hypothalamus. For example, structures located in the caudal brainstem also express GHS-R1a. In particular, ghrelin receptors are found in all three components of the dorsal vagal complex with a highest expression within the area postrema, a moderately dense signal in the nucleus of the solitary tract and a low density signal in the dorsal motor nucleus of the vagus (Zigman et al., 2006). Peripheral injection of ghrelin and, prior to its discover, ghrelin mimetics, induces c-Fos induction in the nucleus of the solitary tract and area postrema (Bailey et al., 2000; Lawrence et al., 2002). The effects of ghrelin on food intake/behavior are similar when injected into the fourth as for the third ventricle, in terms of the amount of food eaten, the number of meals and meal size during the first few hours after treatment (Faulconbridge et al., 2003). The effects of ghrelin in the dorsal vagal complex might be more related to autonomic effects such as on the cardiovascular system. However, since the hypothalamus is strongly connected with the nucleus solitary tract, we cannot exclude an indirect effect of the ghrelin to hypothalamic structures through an activation of brainstem areas, although appears not to include a noradrenergic pathway (Bailey et al., 2000; see *infra*).

#### Ghrelin and the reward system

**Figure 3:**
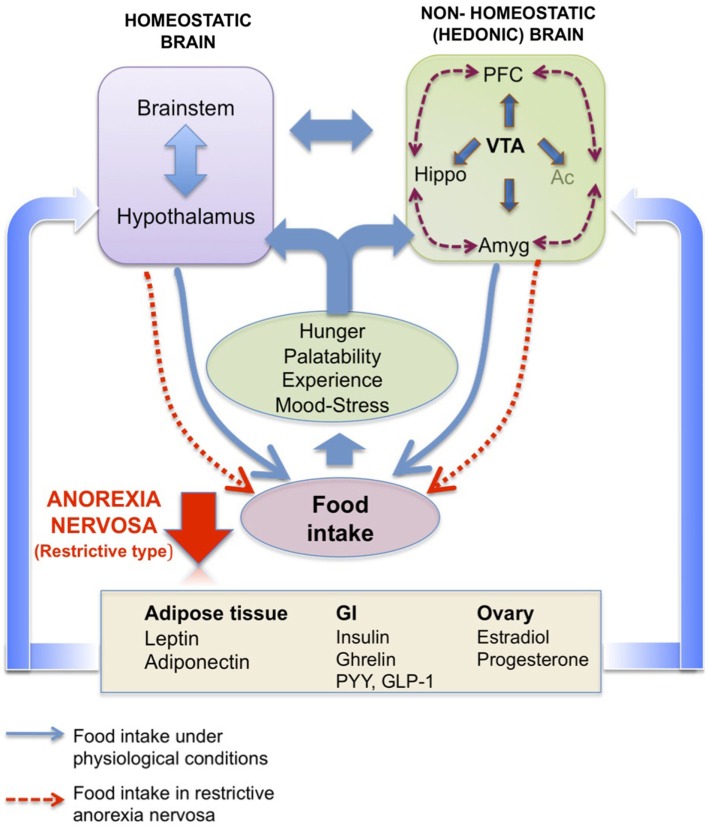
**Homeostatic brain vs. non-homeostatic (hedonic) brain**. Schematic representation of the potential interaction of homeostatic hypothalamic and brainstem areas with non-homeostatic (hedonic) brain structures to control food intake. The hedonic brain comprises mainly the meso-cortico-limbic system, which includes the ventral tegmental area (VTA), nucleus accumbens (Ac), prefrontal cortex (PFC), hippocampus (Hippo), and amygdala (Amyg). Hormones from peripheral compartments like adipose tissue, gastrointestinal tract or ovary reach these areas, directly or indirectly to activate pathways controlling both energy balance (homeostatic brain) and pleasure (hedonic brain) associated with eating (hunger level, palatability of the food, past experiences, mood, level of stress). In anorexia nervosa (restrictive type), a deregulation of one or more of these pathways as well as the cross-talk between periphery and brain might be considered. Adapted from Van Vugt (2010).

Besides the homeostatic ghrelin sensitive pathways, ghrelin also appears to target mesolimbic circuits linked to reward. Hedonic (non-homeostatic) brain pathways are also involved in feeding control and are modulated by circulating energy balance signals such as ghrelin, thereby influencing the evaluation of the pleasure derived from the taste, smell, or texture of food. Many regions of the corticolimbic and mesolimbic brain are thus involved in learning, memory, emotion, and reward processing associated with food. Among these complex feeding networks (for review, see Van Vugt, 2010; Figure 3), the VTA-Acc (dopaminergic) pathway plays a pivotal role in conferring reward from a wide range of reinforcers, from chemical drugs of abuse to natural rewards such as food (Nestler, 1996; Corwin et al., 2011). Using optogenetic techniques, Adamantidis et al. (2011) demonstrated that phasic activation dopaminergic VTA neurons is associated with reward-predicting cues and facilitates the development of positive reinforcement during reward-seeking and behavioral flexibility. As reviewed in Carr (2011), burst firing of VTA dopaminergic neurons may operate as a “teaching signal.” For example, in the case of food intake, when rats are presented with a highly palatable food for the first time, this triggers dopamine release in the Acc (shell), whereas repeated exposure to the same palatable food blunts the dopamine response despite avid consumption. Interestingly, food restriction has been described to sustain the Acc (shell) dopamine release in this model. Moreover, simply delivering cues linked previously to the food reward can be sufficient to reinstate the Acc (shell) dopamine response, indicating transference of the dopamine response from the reinforcer to the cue (for review, see Volkov et al. (2011). Ghrelin has emerged as one important modulator of the VTA-NAcc (dopaminergic) reward pathway (for review, see Skibicka and Dickson, 2011). More than 50% of the dopaminergic VTA neurons express GHS-R (Zigman et al., 2006), although it is also expressed on other cell types in this area (Abizaid et al., 2006). Whether administered peripherally, into the brain ventricles or into the VTA, ghrelin administration triggers a robust Acc dopamine response (Abizaid et al., 2006; Jerlhag et al., 2006, 2007) that is accompanied by an increased feeding response in rodents (Naleid et al., 2005; Egecioglu et al., 2010) and an increase in food-motivated behavior (Skibicka and Dickson, 2011; Skibicka et al., 2012). Central ghrelin signaling, *via* GHS-R1A, appears to be important not only for food reward (Egecioglu et al., 2010), but also for the reward associated with artificial rewards like alcohol, cocaine, and amphetamine (Wellman et al., 2005; Jerlhag et al., 2009, 2010). As an example, the locomotor stimulating effect of cocaine is decreased in ghrelin KO mice compared to their wild type littermates (Abizaid et al., 2010). Furthermore, in rodents, ghrelin elevates the motivation to obtain high-sugar or high-fat reward (Perello et al., 2010; Skibicka and Dickson, 2011; Skibicka et al., 2012). In particular, both peripheral and central injections of ghrelin augment the food-motivated behavior of a satiated rat to get sugar whereas blockade of ghrelin signaling reduces the operant responding of an hungry rat to the level of a satiated rat (Skibicka et al., 2012). These data strongly support the involvement of ghrelin in behaviors related to food reward. Thereby one can suggest that ghrelin could be considered as a key internal cue, available during period of energy deficit to motivate adequate food intake behavior (Skibicka and Dickson, 2011). These data reinforce the now well-documented role of ghrelin in food reward, considering that the shell region of the Acc is described to process unpredicted rewards and motivational states to reinforce food intake behaviors, but also use of drugs of abuse. However, the action of circulating ghrelin upon GHS-R1A-expressing cells in the VTA, located mostly on dopaminergic neurons, begs the question of how this hormone reaches deep brain structures that are far away from circumventricular organs (see *infra*). Finally, one study shows that presence of food is necessary to induce dopamine release (Kawahara et al., 2009). Chronic ghrelin treatment also modifies the expression of dopaminergic receptors in Acc, more specially D1 and D3 (Skibicka et al., 2012), which are described to be involved in obesity, food reward (D1 receptors) and inhibition of reward behavior (D3 receptors). Similar data were obtained from human imaging studies that emphasize the role of ghrelin in food reward. Indeed, intravenous ghrelin injection to human subjects increases activity in brain areas involved in the evaluation of the reward value attributed to food and food cues including the striatum, amygdala, insula, and orbitofrontal cortex (Malik et al., 2008).

#### Other functions

Almost 6000 articles have been published on ghrelin, since its discovery in Kojima et al. (1999) and it is not surprising that its biological effects extend beyond feeding and energy balance, effects that may also be relevant for AN. These include peripheral effects (e.g., gastric motility, bone homeostasis, cardiovascular system, glucose homeostasis, reproduction, immune system) as well as CNS effects (mood disorder, sleep disturbance).

There is now increasing evidence that ghrelin stimulates motor activity in the gastrointestinal tract (gastric motility and emptying). In fact, ghrelin shares high homology degree with motilin (Kojima and Kangawa, 2005), a hormone released by endocrine cells of duodenum and jejunum during fasting and which increases gastric motility after feeding (Sanger, 2008). Ghrelin has been described to use both central and local pathways to exert its effects on the gut through receptors located on vagal afferents, in the nodose ganglion and myenteric plexus. In fact in normal rodents, central pathways are operational whereas after vagotomy, ghrelin is able to exert effects *via* the myenteric plexus (see review of Peeters, 2003). Moreover, in mice, central administration of ghrelin accelerates gastric emptying (Asakawa et al., 2001) and changes the excitability of neurons located in the PVN identified as receiving ascending afferent signals from mechanoreceptors in the stomach (Zhao et al., 2003). Clinically, it has been reported that the intravenous administration of ghrelin accelerates the rate of gastric emptying and induces gastrointestinal contraction in healthy volunteers (Fujitsuka et al., 2012). Since the plasma levels of ghrelin are high in AN patients, one can hypothesize an alteration of the signaling both at central and peripheral levels that may worsen the outcome of the patients. These findings suggest that ghrelin could provide a therapeutic target for disorders related to gastrointestinal discomfort.

Surprisingly, ghrelin is also involved in sleep-wakefulness regulation. Indeed, experiments conducted in adult male rats demonstrate that repeated intravenously administrations of ghrelin stimulate wakefulness, decrease slow-wave sleep, and reduce the duration of rapid eye movement sleep (Tolle et al., 2002). This action could involve a reduction in the release of acetylcholine from the dorsal tegmental nucleus (LDTg) on neurons expressing somatotropin release-inhibiting factor known to indirectly regulate the rapid eye movement sleep periods (Tolle et al., 2002). In fact, among the various neurochemical systems involved in wakefulness (acetylcholine, norepinephrine, dopamine, serotonin, histamine), the hypothalamic orexigenic neurons are crucial promoters of wakefulness since deficiency in the orexin system leads to disorders such as narcolepsy (Modirrousta et al., 2005). The status of activity in orexin neurons is closely related with the nutritional and behavioral state of animals. Moreover, Lamont et al. (2012) observed that both GHS-R and ghrelin KO mice had fewer orexin-immunoreactive cells than their wild type littermates. Their data support the synergistic relationship between ghrelin and orexin in the coordination of metabolism, reward and arousal to adopt the adapted behavior for food seeking and restoration of energy deficiency. In humans, AN patients exhibit sleep disorders. As an example, AN adolescents have an increase in wakefulness after sleep onset, a fragmentation of sleep as well as a reduction of slow-wave sleep and slow-wave activity during their total sleep time (Lauer and Krieg, 2004; Nobili et al., 2004). Even if deepening of nocturnal sleep follows a partial weight restoration, the neurobiological mechanisms linking starvation, mood disorders, and sleep disturbance remain to be elucidated.

The impact of ghrelin on anxiety behaviors remains controversial: studies show an anxiolytic effect under caloric restriction or after subcutaneous ghrelin injection (Lutter et al., 2008) while anxiogenic effects are observed in others (acute) studies with intracerebroventricular or intraperitoneal ghrelin injection (Asakawa et al., 2001; see review Chuang and Zigman, 2010). Interestingly, chronic central ghrelin treatment was found to increase anxiety-like behavior in rats (Hansson et al., 2011). Recently, one study investigating the amygdala as a target for ghrelin found that acute ghrelin injection at this site elicits behaviors consist with a reduction in anxiety-like behavior, but only in rats that were not allowed access to food during the initial hour after injection. It was concluded that ghrelin, acting at the level of the amygdala, may provide an especially important signal to suppress anxiety-like behaviors that would otherwise prohibit the animal from finding food (Alvarez-Crespo et al., 2012). It is not yet known whether the ghrelin system regulates anxiety behavior associated with AN. One study found that a SNP in the preproghrelin gene was associated with panic disorder in a small patient group (Hansson et al., 2011).

Among the disorders described in AN, osteopenia/osteoporosis is also one major problem that cause long term outcomes with in particular a strong increase of the bone fracture incidence (Lucas et al., 1999). Ghrelin has and ghrelin mimetics have been shown to increase bone mineral density (Svensson et al., 2000; Fukushima et al., 2005; Delhanty et al., 2006) by a mechanism that appears to include the promotion of both proliferation and differentiation of osteoblasts (cells involved in bone formation), involving GHS-R1a and GHS-R1b receptors (Fukushima et al., 2005; Delhanty et al., 2006). The etiopathogenesis of bone disease in AN is complex and multifaceted. Indeed, the low bone mineral density (Legroux-Gerot et al., 2005, 2008; Legroux-Gérot et al., 2007; Estour et al., 2010) is usually linked with alteration of multiple factors (Tables 3– 6) that are thought to contribute to the “uncoupling” of bone turnover, leading to increased bone resorption, and decreased bone formation (see Howgate et al., 2013). However, it has been demonstrated that ghrelin affects bone metabolism by operating in an autocrine/paracrine mode, independent of the GH/IGF-1 axis (see Nikolopoulos et al., 2010). Weight recovery is associated with partial recovery of bone mineral density. There is currently no approved effective therapy that completely reverses the bone mineral density deficit. The most convincing results were obtained with a treatment of recombinant human IGF-1 alone or in combination with the oral contraceptive pills (see Misra and Klibanski, 2011). The link between ghrelin and estrogen on bone metabolism is always matter of debate even if it is established that ghrelin suppresses pulsatile LH and FSH pulsatility (Meczekalski et al., 2008; Kluge et al., 2012).

Another criterion used to characterize AN patients is amenorrhea. Indeed, in negative energy balance conditions like in AN, the increase of plasma ghrelin is associated with decrease of LH secretion (Table 6). Evidence is mounting that ghrelin may operate as a pleitropic modulator of gonadal function and reproduction (Tena-Sempere, 2008; Muccioli et al., 2011; Repaci et al., 2011). Notably, most of the actions of ghrelin upon the reproductive axis reported to date are inhibitory. Ghrelin can suppress not only LH, but also FSH secretion in male and female rats (Fernández-Fernández et al., 2005; Martini et al., 2006). Such effects are also described in humans (Kluge et al., 2012). Centrally, ghrelin exerts a predominant action directly at the level of the GnRH pulse generator by inhibiting directly GnRH release (Fernández-Fernández et al., 2005; Muccioli et al., 2011) or by an indirect modulation of other neuronal pathways. For example, Forbes et al. (2009) recently showed the ability of ghrelin to decrease Kiss1 mRNA expression in the medial preoptic area. Given the importance of the kisspeptin system to control the reproductive axis, these data provide new hypothesis for ghrelin-induced suppression of pulsatile LH secretion. Once again, in the AN, ghrelin might dynamically mediate the suppressive effect of energy deficit on the onset of puberty, gonadal function, and fertility. Here, the effects of ghrelin on the gonadal axis might protect females in a condition of strong energy insufficiency to develop a reproductive behavior that can be deleterious for her and her progeny.

Finally, ghrelin is also involved in other physiological functions that are more or less affected in AN like cardiovascular function or immune system. Among the cardiovascular effects, this hormone improves left ventricular contractility and cardiac output in healthy humans (Enomoto et al., 2003; Tesauro et al., 2010) and lowers blood pressure in mice concomitantly with a decrease in sympathetic nerve activity that is not caused by a direct action on blood vessels (Callaghan et al., 2012). In AN, the neuroendocrine alterations are also accompanied by autonomic dysfunctions like lower blood pressure values, lack of circadian variation of blood pressure and bradycardia (Oswiecimska et al., 2007). Thus, ghrelin might participate in AN to the cardiovascular complications observed in AN (Casiero and Frishman, 2006; Jáuregui-Garrido and Jáuregui-Lobera, 2012), but no studies currently display any correlation between these cardiovascular risks and the high plasma levels of ghrelin.

The role of ghrelin on the immune system remains unclear. However, Taub (2008) describes its implication in the regulation of immune factors, by inhibiting inflammatory cytokine production, more specifically in mediating anti-inflammatory effects on IL-1, TNF-α, and IL-6 cytokine expression by T-cells and mononuclear cells *via* GHS-R, and promoting thymic function. In AN, data related to the evaluation of circulating pro-inflammatory or inflammatory cytokines or in adipocytes are still a matter of debate and, as underlined by Nova and Marcos (2006), “controversial findings have been published regarding some aspects of the immune system that are otherwise impaired in more typical types of malnutrition.”

#### Des-acyl ghrelin and obestatin: a controversial metabolic function?

Concerning des-acyl ghrelin, its role in food intake has been much debated. The recent paper of Delhanty et al. (2012) gives numerous arguments supporting des-acyl ghrelin as an hormone that can be metabolically active, when co-administrated with acyl ghrelin, by counteracting the effects of acyl ghrelin on insulin secretion and glucose metabolism. Des-acyl ghrelin appears to be increased in AN patients (Harada et al., 2008; Germain et al., 2009). Kojima et al. (1999) showed that des-acyl ghrelin was not able to bind to GHS-R1a. Although the des-acyl ghrelin receptor remains unknown, the increasing data suggesting that des-acyl ghrelin is a biologically active molecular, indicate that a dedicated receptor may exist.

Several studies show controversial effects of des-acyl ghrelin on food intake that are either inhibitory (Asakawa et al., 2005; Chen et al., 2005b) or stimulatory (Toshinai et al., 2006) in rodents. These results can be due to the different methods used like the type of injection, the dose used, the time of injection (light or dark period), the nutritional status, fed, or fasted. However, the overexpression of des-acyl ghrelin in a transgenic mouse model results in a small phenotype, associated with a reduction of food intake and body fat mass, reduced IGF-1 plasma levels without significant changes in circulating GH and also higher des-acyl ghrelin with no change in total ghrelin plasma levels (Ariyasu et al., 2005). In these mice, no significant differences have been noticed for glycemia and insulinemia (Ariyasu et al., 2005; Asakawa et al., 2005) while studies other studies have shown that des-acyl ghrelin inhibits glucose release *in vivo* and *in vitro* (Broglio et al., 2004b; Gauna et al., 2005; Qader et al., 2008). Moreover, it appears that ghrelin and des-acyl ghrelin do not have the same blood concentration in systemic and in portal circulations suggesting that liver could be involved in ghrelin regulation (Goodyear et al., 2010).

Concerning lipid metabolism, *in vivo* studies showed that des-acyl ghrelin as well as ghrelin increase bone marrow adipogenesis in rat shinbone (Thompson et al., 2004) and both forms enhance lipid accumulation in visceral tissue in humans (Rodríguez et al., 2009). The mechanisms involved remain unclear. Similarly, acute or chronic des-acyl ghrelin injections in adult male rats cause an inhibition of LH secretion like ghrelin (Martini et al., 2006). By contrast, transgenic mice overexpressing des-acyl ghrelin do not display any changes in LH and FSH levels (Ariyasu et al., 2005).

Des-acyl ghrelin may also have a role in gastric motility. Indeed, intracerebroventricular or intravenous injections alter motor activity in the antrum with a decrease of antrum activity only in fasted rats (Chen et al., 2005b). Mice overexpressing des-acyl ghrelin exhibit a decrease in gastric emptying (Asakawa et al., 2005). Other studies are necessary to understand the mechanisms involved since vagotomy does not disrupt the (intravenous) des-acyl ghrelin effect (Chen et al., 2005b).

Studies about the effects of des-acyl ghrelin on the cardiovascular system are rare. Nevertheless, like acyl ghrelin, it promotes bradycardia and hypotension (Tsubota et al., 2005). Moreover, ghrelin and des-acyl ghrelin display vasodilator effect and no inotropic effects when they are applied on human artery *in vitro* (Kleinz et al., 2006).

Similarly, concerning obestatin, it remains again an open question whether this peptide is a physiologically relevant peptide to regulate energy homeostasis, food intake and gastric motility (Gourcerol et al., 2007). Obestatin binds to GRP 39, a receptor of the same subfamily than ghrelin receptor, to decrease food intake and body weight in an opposite manner to ghrelin (Stengel et al., 2009; Hassouna et al., 2010; Mokrosinski and Holst, 2010; Veldhuis and Bowers, 2010). Subsequent studies failed to show activation of this receptor and only few studies have reproduced the obestatin effects under specific conditions. Such results should be interpreted with caution since variations are observed according to the kits and conditions applied for obestatin assays (Hassouna et al., 2010). Due to their potential functions, it should be interesting to measure ghrelin/obestatin ratio to better understand their roles in the alteration of energy balance. It seems that AN affects obestatin blood levels with a lower ghrelin/obestatin ratio in AN patients of restrictive type compared to constitutional thin women (Germain et al., 2009, 2010). Moreover, other functions are attributed to this hormone such as the inhibition of thirst, gastric motility, cell survival, pancreatic hormone secretion, sleep, thermoregulation, memory, and anxiety (Szentirmai et al., 2009; Hassouna et al., 2010; Veldhuis and Bowers, 2010).

## Access of Ghrelin to Its Neuronal Targets

**Figure 4:**
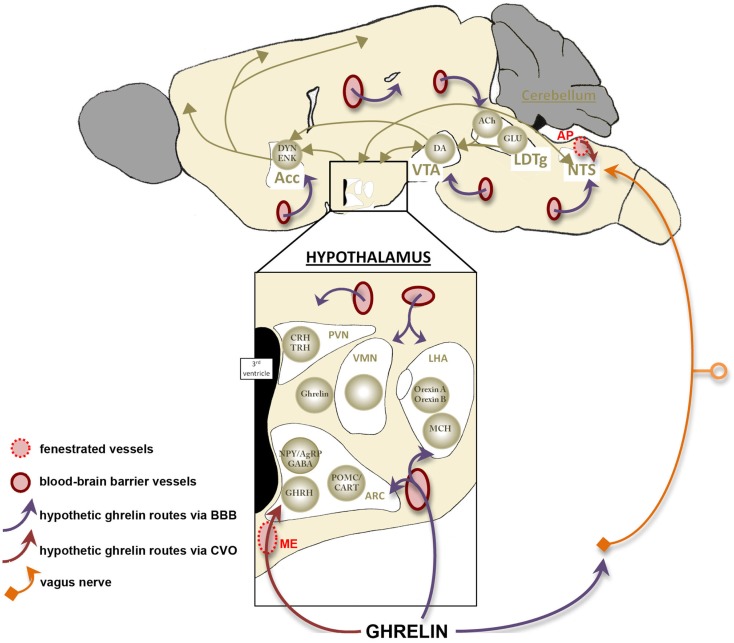
**Access of ghrelin signal to its neuronal targets**. This schema summarizes the three hypothetic access routes of ghrelin toward its neuronal targets (cf Figure 2). First, ghrelin would be able to target neuronal networks thanks to specific transcellular transports at the level of blood–brain barrier (BBB) located on brain capillaries (purple arrows). Most ghrelin sensitive areas present blood–brain barrier vasculature and this route represent the main one described in all regions of the brain. However free-BBB regions, called the median eminence and the area postrema, are recorded in the hypothalamus and the brainstem respectively. These areas contain a rich fenestrated vasculature, which could represent a direct vascular route while allowing passive diffusion of peripheral ghrelin (red arrows). This route may be responsible of acute regulation and complete chronic feedback accomplished by uptake of circulating molecules via receptor-mediated transport across the blood–brain barrier. Finally, activation of brainstem areas by ghrelin may occur without the entrance of ghrelin in the brain, but through its binding to gastric vagal afferent neurons (orange). Acc, accumbens nucleus; ACh, acétylcholine; AgRP, agouti-related peptide; AP, area postrema; ARC, arcuate nucleus; CART, cocaine- and amphetamine-regulated transcript; CRH, corticotropin-releasing hormone; DA, dopamine; DYN, dynorphin; ENK, enkephalin; GABA, γ-aminobutyric acid; GHRH, growth-hormone-releasing hormone; GLU, glutamate; LDTg, laterodorsal tegmental area; LHA, lateral hypothalamic area; MCH, melanin-concentrating hormone; ME, median eminence; NPY, neuropeptide Y; NTS, nucleus of the solitary tract; POMC, pro-opiomelanocortin; PVN, paraventricular nucleus; TRH, thyrotropin releasing hormone; VMH, ventromedial nucleus; VTA, ventral tegmental area.

To dynamically report energy homeostasis alterations and ensure an appropriate neuronal response, blood-borne ghrelin must rapidly access the central nervous system. Intriguingly, the physiological mechanisms controlling the access of ghrelin to its neuronal target remain currently debated. Indeed, although a central origin of ghrelin has been described (Cowley et al., 2003), it is now recognized that blood-derived ghrelin is able to target neuronal networks within the central nervous system to regulate energy homeostasis. However, it remains unclear how this key energy status-signaling hormone can rapidly access sensory neurons to alter feeding responses. Ghrelin mainly targets neurons located in the ARC where different blood/brain interfaces have been described. The blood–brain barrier is one such interface and one of the best described in the hypothalamic nuclei as in all other regions of the brain. The blood–brain barrier is located on brain capillaries where endothelial cells are tightly apposed by continuous tight junctions that prevent the free passage of molecules through the paracellular pathway. For circulating factors to access to the brain through the blood–brain barrier endothelium requires transcellular transport. Many studies have investigated the transport of circulating ghrelin across the blood–brain barrier. Banks et al. (2002) demonstrated the existence of ghrelin saturable transport system in mice from the brain to the blood but transport into the brain was much less pronounced. Remarkably, human ghrelin, which differs from mouse ghrelin by 2 amino acids, can be transported in both directions in mice. So, although receptor-mediated transport of ghrelin cannot be excluded, uptake mechanisms of this peptide remain unclear. Moreover, the efficiency of this blood–brain barrier remains to be studied in a chronic caloric restriction context. Improved CNS penetration during fating is one possible mechanism to explain the threefold increase in the number of cells expressing fos after peripheral ghrelin injection in fasted vs. fed rats (Hewson and Dickson, 2000). The role of another blood/brain interface that is materialized by fenestrated vessels is also to consider. Indeed, these vessels are part of the blood-CSF barrier that is mostly described in the median eminence, the circumventricular organ adjacent to the ARC (Mullier et al., 2010). Median eminence vasculature differs from typical brain vessels as they harbor a fenestrated endothelium that lacks tight junction complexes. These structural characteristics and the presence of various blood-derived molecules in the median eminence and the other circumventricular organs parenchyma suggest high permeability of this specific vasculature (Broadwell et al., 1983; Ciofi, 2011; Morita and Miyata, 2012). Permeable vasculature can be found in the external part of the median eminence forming pituitary portal capillary plexus that displays some long intrainfundibular loops spreading into the median eminence parenchyma. Interestingly, fenestrated vessels are also found within the ARC with a higher density into the ventromedial ARC where they are bordered by NPY expressing neurons (Ciofi et al., 2009). These data give support to the access of ghrelin from the circulation to the ventromedial sensory neurons *via* median eminence/ARC fenestrated vasculature (Figure 4).

The action of ghrelin on food intake may not be only due to its action on hypothalamus. Indeed, an indirect role of ghrelin on hypothalamic structures through an activation of brainstem areas has been suggested. Indirect pathway may occur through the binding of ghrelin to gastric vagal afferent neurons (Date et al., 2002). However, the expression of GHS-R1a within the dorsal vagal complex supports a direct action of ghrelin to brain parenchyma. Among this complex, the area postrema is a circumventricular organ that characteristically present fenestrated vasculature. These vessels may be responsible for the diffusion of ghrelin and its delivery to the dorsal vagal complex that communicates with hypothalamic control centers (Figure 4).

Fenestrated vasculature could represent a direct vascular route while allowing passive diffusion of peripheral molecules into the hypothalamus and the area postrema. This route may be responsible of acute regulation and complete chronic feedback accomplished by uptake of circulating molecules *via* receptor-mediated transport across the blood–brain barrier.

The transport of ghrelin through the blood/brain interfaces has been poorly investigated in metabolic disorders excepted in obesity where few data are available. Banks et al. (2008) showed an inverse relation between body weight and ghrelin access to the brain suggesting that physiological states influence the rate at which ghrelin is transported across the blood/brain interfaces. A better understanding of the access of ghrelin to its neuronal target may leads to novel therapeutic interventions.

## Conclusion: Ghrelin as a Potential Treatment for Anorexia Nervosa

In restrictive AN, the high plasma levels of ghrelin, even adaptive in view of its main role in meal initiation, let us to hypothesize a potential insensitivity to this endocrine signal both peripherally and centrally in association with this disease. It should be also mentioned that AN patients often cannot increase their food intake not only because of fear of obesity, but also because of chronic or recurrent abdominal discomfort, fullness, and chronic constipation, functions in which efficient ghrelin participation is required. Injections of exogenous ghrelin have been shown to increase the adiposity and to stimulate appetite in healthy individuals and cancer patients (Peino et al., 2000; Wren et al., 2001a; Neary et al., 2004).

For AN patients, only a few preliminary studies have been performed to examine the effects of ghrelin administration (Miljic et al., 2006; Hotta et al., 2009; Ogiso et al., 2011). In these clinical studies, the mode of administration was different leading to different outcomes. In the study of Miljic et al. (2006), a single-dose continuous administration of ghrelin for 5 h failed to affect appetite in all AN patients treated. Hotta et al. (2009) report that intravenous administration of ghrelin in anorectic patients twice a day for 14 days improves epigastric discomfort or constipation and increases the hunger score, which is related to gastric emptying. An increase of the body weight was obtained, from 1.5 to 2.4 kg, and daily energy intake during ghrelin infusion increased by 12–36% compared with the pretreatment period. Nutritional parameters such as total protein and triglyceride levels improved. These findings suggest that ghrelin may have therapeutic potentials in AN patients who cannot gain weight because of gastrointestinal dysfunction. Further studies are need to elucidate the potential impact of ghrelin by itself or agonists to ameliorate the outcomes of the AN patients.

In animals, several attempts have been made (see the other chapter submitted by Hassouna et al. “Actions of agonists and antagonists of the ghrelin/GHS-R pathway on GH secretion, appetite, and c-Fos activity”). Recently, Costantini et al. (2011) detailed an unexpected effect of GSK1614343, a novel ghrelin receptor antagonist with no partial agonist properties, that induced both in rats and dogs an increase of food intake, body weight, and reduced the POMC mRNA levels in the hypothalamus of rats chronically treated with the compound. Although it may seem counterintuitive to consider the use of ghrelin antagonists in this disease area, one study in rodents suggested that suppressed ghrelin signaling reduces (food anticipatory) hyperlocomotor activity in the ABA model (Verhagen et al., 2011). Barnett et al. (2010) demonstrated that the administration of a GOAT inhibitor improved glucose tolerance and decreased weight gain in wild type mice but not in ghrelin KO mice. Such treatment also led to decreased serum levels of acyl ghrelin without any effect on serum levels of des-acyl ghrelin. Even if this GOAT inhibitor might be expected to pave the way for clinical targeting of GOAT in metabolic diseases such as obesity and diabetes mellitus, one can suggest that, a GOAT activator, which has not been reported yet, may be a potential new treatment of AN if it improves food intake and body weight gain in patients with AN.

Finally, in the future it will be possible to assess more precisely the exact contribution of ghrelin, des-acyl ghrelin, and obestatin in the evolution of the AN both in humans and adequate animal models (Cardona Cano et al., 2012). The roles played by these peptides in feeding behavior, adaptation to starvation, reward mechanisms, emotional behavior, and stress responses in animals and humans led them to be potential therapeutic targets for AN treatments. The way and the mode of administration remain to be further determined and clarified. Neuroimaging studies have reported reduced brain volumes affecting both ventral and dorsal neural circuit dysfunctions in AN patients, with altered metabolisms of serotonin and dopamine that are closely associated to ghrelin, contributing to their puzzling symptoms (Kaye et al., 2009; Brooks et al., 2011). It will be important to determine whether the ghrelin signal reaches its central targets leading, as it is observed for leptin in obesity, to a « ghrelin-resistance » or a « transient ghrelin-insensitivity ». More investigations are needed to better suppress the neuronal activity of ghrelin signaling and to identify the specific pathways that may underlie the deleterious behaviors in patients suffering from AN. Investigation of the ghrelin peptide system will open up a new window of research for tackling psychosomatic disorders beyond the gastrointestinal tract, particularly restrictive AN and obesity/metabolic syndrome, two disorders at the extreme of the body weight continuum.

## Conflict of Interest Statement

The authors declare that the research was conducted in the absence of any commercial or financial relationships that could be construed as a potential conflict of interest.

## Author Note

Cross reference with another chapter of this issue: *Actions of agonists and antagonists of the ghrelin/GHS-R pathway on GH secretion, appetite and c-Fos activity* by Rim Hassouna, Alexandra Labarthe, Philippe Zizzari, Catherine Videau, Michael Culler, Jacques Epelbaum, and Virginie Tolle (Hassouna et al., 2013).

## References

[B1] Abizaid A. Liu Z. W. Andrews Z. B. Shanabrough M. Borok E. Elsworth J. D. (2006). Ghrelin modulates the activity and synaptic input organization of midbrain dopamine neurons while promoting appetite. J. Clin. Invest. 116, 3229–3239 10.1172/JCI29867 17060947 PMC1618869

[B2] Abizaid A. Mineur Y. S. Roth R. H. Elsworth J. D. Sleeman M. W. Picciotto M. R. (2010). Reduced locomotor responses to cocaine in ghrelin-deficient mice. Neuroscience 192, 500–506 10.1016/j.neuroscience.2011.06.001 21699961

[B3] Adamantidis A. R. Tsai H. C. Boutrel B. Zhang F. Stuber G. D. Budygin E. A. (2011). Optogenetic interrogation of dopaminergic modulation of the multiple phases of reward-seeking behavior. J. Neurosci. 31, 10829–10835 10.1523/JNEUROSCI.2246-11.2011 21795535 PMC3171183

[B4] Alguacil L. F. Salas E. González-Martín C. (2011). Identification of new drug targets and biomarkers related to obesity and eating disorders: an approach based on reward deficit and addiction. Curr. Pharm. Des. 17, 462–470 10.2174/138161211795164158 21375484

[B5] Alvarez-Crespo M. Skibicka K. P. Farkas I. Molnár C. S. Egecioglu E. Hrabovszky E. (2012). The amygdala as a neurobiological target for ghrelin in rats: neuroanatomical, electrophysiological and behavioral evidence. PLoS ONE 7:e46321 10.1371/journal.pone.0046321 23071554 PMC3468604

[B6] Andrews Z. B. Liu Z. W. Walllingford N. Erion D. M. Borok E. Friedman J. M. (2008). UCP2 mediates ghrelin’s action on NPY/AgRP neurons by lowering free radicals. Nature 454, 846–851 10.1038/nature07181 18668043 PMC4101536

[B7] Arimura C. Nozaki T. Takakura S. Kawai K. Takii M. Sudo N. (2010). Predictors of menstrual resumption by patients with anorexia nervosa. Eat. Weight Disord. 15, e226–e233 20458162 10.3275/7039

[B8] Ariyasu H. Takaya K. Iwakura H. Hosoda H. Akamizu T. Arai Y. (2005). Transgenic mice overexpressing des-acyl ghrelin show small phenotype. Endocrinology 146, 355–364 10.1210/en.2004-0629 15471959

[B9] Ariyasu H. Takaya K. Tagami T. Ogawa Y. Hosoda K. Akamizu T. (2001). Stomach is a major source of circulating ghrelin, and feeding state determines plasma ghrelin-like immunoreactivity levels in humans. J. Clin. Endocrinol. Metab. 86, 4753–4758 10.1210/jc.86.10.4753 11600536

[B10] Arvat E. Maccario M. Di Vito L. Broglio F. Benso A. Gottero C. (2001). Endocrine activities of ghrelin, a natural growth hormone secretagogue (GHS), in humans: comparison and interactions with hexarelin, a nonnatural peptidyl GHS, and GH-releasing hormone. J. Clin. Endocrinol. Metab. 86, 1169–1174 10.1210/jc.86.7.3176 11238504

[B11] Asakawa A. Inui A. Fujimiya M. Sakamaki R. Shinfuku N. Ueta Y. (2005). Stomach regulates energy balance via acylated ghrelin and desacyl ghrelin. Gut 54, 18–24 10.1136/gut.2004.038737 15591499 PMC1774379

[B12] Asakawa A. Inui A. Kaga T. Yuzuriha H. Nagata T. Ueno N. (2001). Ghrelin is an appetite-stimulatory signal from stomach with structural resemblance to motilin. Gastroenterology 120, 337–345 10.1016/S0016-5085(01)81675-9 11159873

[B13] Avena N. M. Bocarsly M. E. (2012). Dysregulation of brain reward systems in eating disorders: neurochemical information from animal models of binge eating, bulimia nervosa, and anorexia nervosa. Neuropharmacology 63, 87–96 10.1016/j.neuropharm.2011.11.010 22138162 PMC3366171

[B14] Bailey A. R. von Engelhardt N. Leng G. Smith R. G. Dickson S. L. (2000). Growth hormone secretagogue activation of the arcuate nucleus and brainstem occurs via a non-noradrenergic pathway. J. Neuroendocrinol. 12, 191–197 10.1046/j.1365-2826.2000.00398.x 10718914

[B15] Balligand J. L. Brichard S. M. Brichard V. Desager J. P. Lambert M. (1998). Hypoleptinemia in patients with anorexia nervosa: loss of circadian rhythm and unresponsiveness to short-term refeeding. Eur. J. Endocrinol. 138, 415–420 10.1530/eje.0.1380415 9578509

[B16] Banks W. A. Burney B. O. Robinson S. M. (2008). Effects of triglycerides, obesity, and starvation on ghrelin transport across the blood-brain barrier. Peptides 29, 2061–2065 10.1016/j.peptides.2008.07.001 18682266 PMC2586070

[B17] Banks W. A. Tschop M. Robinson S. M. Heiman M. L. (2002). Extent and direction of ghrelin transport across the blood-brain barrier is determined by its unique primary structure. J. Pharmacol. Exp. Ther. 302, 822–827 10.1124/jpet.102.036129 12130749

[B18] Baranowska B. (1990). Are disturbances in opioid and adrenergic systems involved in the hormonal dysfunction of anorexia nervosa. Psychoneuroendocrinology 15, 371–379 10.1016/0306-4530(90)90062-E 2129311

[B19] Baranowska B. Radzikowska M. Wasilewska-Dziubinska E. Roguski K. Borowiec M. (2000). The role of VIP and somatostatin in the control of GH and prolactin release in anorexia nervosa and in obesity. Ann. N. Y. Acad. Sci. 921, 443–455 10.1111/j.1749-6632.2000.tb07013.x 11193873

[B20] Baranowska B. Wasilewska-Dziubinska E. Radzikowska M. Plonowski A. Roguski K. (1997). Neuropeptide Y, galanin, and leptin release in obese women and in women with anorexia nervosa. Metab. Clin. Exp. 46, 1384–1389 10.1016/S0026-0495(97)90136-0 9439531

[B21] Baranowska B. Wolinska-Witort E. Wasilewska-Dziubinska E. Roguski K. Chmielowska M. (2001). Plasma leptin, neuropeptide Y (NPY) and galanin concentrations in bulimia nervosa and in anorexia nervosa. Neuro Endocrinol. Lett. 22, 356–358 11600878

[B22] Barazzoni R. Bosutti A. Stebel M. Cattin M. R. Roder E. Visintin L. (2005). Ghrelin regulates mitochondrial-lipid metabolism gene expression and tissue fat distribution in liver and skeletal muscle Am. J. Physiol. Endocrinol. Metab. 288, E228–E235 10.1152/ajpendo.00115.2004 15328073

[B23] Barbarich-Marsteller N. C. (2007). Neurochemistry and pharmacological treatments: where is the field of anorexia nervosa heading? Cent. Nerv. Syst. Agents Med. Chem. 7, 35–43 10.2174/187152407780059204

[B24] Barbarich-Marsteller N. C. Foltin R. W. Walsh B. T. (2011). Does anorexia nervosa resemble an addiction? Curr. Drug Abuse Rev. 4, 197–200 10.2174/1874473711104030197 21999694 PMC4438277

[B25] Barnett B. P. Hwang Y. Taylor M. S. Kirchner H. Pfluger P. T. Bernard V. (2010). Glucose and weight control in mice with a designed ghrelin O-acyltransferase inhibitor. Science 330, 1689–1692 10.1126/science.1196154 21097901 PMC3068526

[B26] Bergh C. Södersten P. (1996). Anorexia nervosa, self starvation and the reward of stress. Nat. Med. 2, 21–22 10.1038/nm0196-21 8564826

[B27] Berrettini W. H. Kaye W. H. Sunderland T. May C. Gwirtsman H. E. Mellow A. (1988). Galanin immunoreactivity in human CSF: studies in eating disorders and Alzheimer’s disease. Neuropsychobiology 19, 64–68 10.1159/000118446 2465504

[B28] Boakes R. A. (2007). Self-starvation in the rat: running versus eating. Span. J. Psychol. 10, 251–257 17992951 10.1017/s113874160000651x

[B29] Boakes R. A. Juraskova I. (2001). The role of drinking in the suppression of food intake by recent activity. Behav. Neurosci. 115, 718–730 10.1037/0735-7044.115.3.718 11439461

[B30] Boghi A. Sterpone S. Sales S. D’Agata F. Bradac G. B. Zullo G. (2011). In vivo evidence of global and focal brain alterations in anorexia nervosa. Psychiatry Res. 192, 154–159 10.1016/j.pscychresns.2010.12.008 21546219

[B31] Bolton J. G. Patel S. Lacey J. H. White S. (2005). A prospective study of changes in bone turnover and bone density associated with regaining weight in women with anorexia nervosa. Osteoporos. Int. 16, 1955–1962 10.1007/s00198-005-1972-7 16027954

[B32] Bordi C. D’Adda T. Azzoni C. Ferraro G. (2000). Classification of gastric endocrine cells at the light and electron microscopical levels. Microsc. Res. Tech. 48, 258–271 10.1002/(SICI)1097-0029(20000301)48:5<258::AID-JEMT3>3.0.CO;2-Q 10700043

[B33] Bosy-Westphal A. Brabant G. Haas V. Onur S. Paul T. Nutzinger D. (2005). Determinants of plasma adiponectin levels in patients with anorexia nervosa examined before and after weight gain. Eur. J. Nutr. 44, 355–359 10.1007/s00394-005-0533-3 15793670

[B34] Bowers C. Y. Chang J. Momany F. Folkers K. (1977). “Effect of the enkephalins and enkephalin analogs on release of pituitary hormones in vitro,” in Molecular Endocrinology, eds MacIntyre I. Szelke M. (Amsterdam: Elsevier/North Holland), 287–292

[B35] Bowers C. Y. Momany F. A. Reynolds G. A. Hong A. (1984). On the in vitro and in vivo activity of a new synthetic hexapeptide that acts on the pituitary to specifically release growth hormone. Endocrinology 114, 1537–1545 10.1210/endo-114-5-1537 6714155

[B36] Brambilla F. Ferrari E. Petraglia F. Facchinetti F. Catalano M. Genazzani A. R. (1991). Peripheral opioid secretory pattern in anorexia nervosa. Psychiatry Res. 39, 115–127 10.1016/0165-1781(91)90081-Y 1665917

[B37] Brambilla F. Santonastaso P. Caregaro L. Favaro A. (2006). Disorders of eating behavior: correlation between hypothalamo-pituitary-thyroid function and psychopathological aspects. Psychoneuroendocrinology 31, 131–136 10.1016/j.psyneuen.2005.06.003 16112494

[B38] Brick D. J. Gerweck A. V. Meenaghan E. Lawson E. A. Misra M. Fazeli P. (2010). Determinants of IGF1 and GH across the weight spectrum: from anorexia nervosa to obesity. Eur. J. Endocrinol. 163, 185–191 10.1530/EJE-10-0365 20501597 PMC2953770

[B39] Briggs D. I. Andrews Z. B. (2011). Metabolic status regulates ghrelin function on energy homeostasis. Neuroendocrinology 93, 48–57 10.1159/000322589 21124019

[B40] Broadwell R. D. Balin B. J. Salcman M. Kaplan R. S. (1983). Brain-blood barrier? Yes and no. Proc. Natl. Acad. Sci. U.S.A. 80, 7352–7356 10.1073/pnas.80.23.7352 6580650 PMC390053

[B41] Broglio F. Arvat E. Benso A. Gottero C. Muccioli G. Papotti M. (2001). Ghrelin, a natural GH secretagogue produced by the stomach, induces hyperglycemia and reduces insulin secretion in humans. J. Clin. Endocrinol. Metab. 86, 5083–5086 10.1210/jc.86.10.5083 11600590

[B42] Broglio F. Gianotti L. Destefanis S. Fassino S. Abbate Daga G. Mondelli V. (2004a). The endocrine response to acute ghrelin administration is blunted in patients with anorexia nervosa, a ghrelin hypersecretory state. Clin. Endocrinol. (Oxf.) 60, 592–599 10.1111/j.1365-2265.2004.02038.x 15104562

[B43] Broglio F. Gottero C. Prodam F. Gauna C. Muccioli G. Papotti M. (2004b). Non-acylated ghrelin counteracts the metabolic but not the neuroendocrine response to acylated ghrelin in humans. J. Clin. Endocrinol. Metab. 89, 3062–3065 10.1210/jc.2003-031517 15181099

[B44] Brooks S. J. Barker G. J. O’Daly O. G. Brammer M. Williams S. C. Benedict C. (2011). Restraint of appetite and reduced regional brain volumes in anorexia nervosa: a voxel-based morphometric study. BMC Psychiatry 17:179 10.1186/1471-244X-11-179 22093442 PMC3278387

[B45] Buehren K. Konrad K. Schaefer K. Kratzsch J. Kahraman-Lanzerath B. Lente C. (2011). Association between neuroendocrinological parameters and learning and memory functions in adolescent anorexia nervosa before and after weight recovery. J. Neural Transm. 118, 963–968 10.1007/s00702-010-0567-4 21207075

[B46] Burden V. R. White B. D. Dean R. G. Martin R. J. (1993). Activity of the hypothalamic-pituitary-adrenal axis is elevated in rats with activity-based anorexia. J. Nutr. 123, 1217–1225 8320563 10.1093/jn/123.7.1217

[B47] Caillot-Augusseau A. Lafage-Proust M. H. Margaillan P. Vergely N. Faure S. Paillet S. (2000). Weight gain reverses bone turnover and restores circadian variation of bone resorption in anorexic patients. Clin. Endocrinol. (Oxf.) 52, 113–121 10.1046/j.1365-2265.2000.00879.x 10651762

[B48] Calero J. A. Muñoz M. T. Argente J. Traba M. L. Méndez-Dávila C. García-Moreno C. (1999). A variation in Bone Alkaline Phosphatase levels that correlates positively with bone loss and normal levels of aminoterminal propeptide of collagen I in girls with anorexia nervosa. Clin. Chim. Acta 285, 121–129 10.1016/S0009-8981(99)00114-X 10481928

[B49] Callaghan B. Hunne B. Hirayama H. Sartor D. M. Nguyen T. V. Abogafie F. C. (2012). Sites of action of ghrelin receptor ligands in cardiovascular control. Am. J. Physiol. Heart Circ. Physiol. 303, H1011–H1021 10.1152/ajpheart.00418.2012 22886413

[B50] Camiña J. P. (2006). Cell biology of the ghrelin receptor. J. Neuroendocrinol. 18, 65–76 10.1111/j.1365-2826.2005.01379.x 16451222

[B51] Cardona Cano S. Merkestein M. Skibicka K. P. Dickson S. L. Adan R. A. (2012). Role of ghrelin in the pathophysiology of eating disorders: implications for pharmacotherapy. CNS Drugs 26, 281–296 10.2165/11599890-000000000-00000 22452525

[B52] Carr K. D. (2011). Food scarcity, neuroadaptations, and the pathogenic potential of dieting in an unnatural ecology: binge eating and drug abuse. Physiol. Behav. 104, 162–167 10.1016/j.physbeh.2011.04.023 21530562 PMC3107914

[B53] Casiero D. Frishman W. H. (2006). Cardiovascular complications of eating disorders. Cardiol. Rev. 14, 227–231 10.1097/01.crd.0000216745.96062.7c 16924163

[B54] Casper R. C. (1998). Behavioral activation and lack of concern, core symptoms of anorexia nervosa? Int. J. Eat. Disord. 24, 381–393 10.1002/(SICI)1098-108X(199812)24:4<381::AID-EAT5>3.0.CO;2-Q 9813763

[B55] Castañeda T. R. Tong J. Datta R. Culler M. Tschöp M. H. (2010). Ghrelin in the regulation of body weight and metabolism. Front. Neuroendocrinol. 31, 44–60 10.1016/j.yfrne.2009.10.008 19896496

[B56] Cerrato M. Carrera O. Vazquez R. Echevarría E. Gutierrez E. (2012). Heat makes a difference in activity-based anorexia: a translational approach to treatment development in anorexia nervosa. Int. J. Eat. Disord. 45, 26–35 10.1002/eat.20884 22170019

[B57] Chen H. Y. Trumbauer M. E. Chen A. S. Weingarth D. T. Adams J. R. Frazier E. G. (2004). Orexigenic action of peripheral ghrelin is mediated by neuropeptide Y and agouti-related protein. Endocrinology 145, 2607–2612 10.1210/en.2003-1596 14962995

[B58] Chen X. Ge Y. L. Jiang Z. Y. Liu C. Q. Depoortere I. Peeters T. L. (2005a). Effects of ghrelin on hypothalamic glucose responding neurons in rats. Brain Res. 1055, 131–136 10.1016/j.brainres.2005.06.080 16084498

[B59] Chen C. Y. Inui A. Asakawa A. Fujino K. Kato I. Chen C. C. (2005b). Des-acyl ghrelin acts by CRF type 2 receptors to disrupt fasted stomach motility in conscious rats. Gastroenterology 129, 8–25 10.1053/j.gastro.2005.05.039 16012930

[B60] Chiodera P. Volpi R. Capretti L. Marchesi C. d’Amato L. De Ferri A. (1991). Effect of estrogen or insulin-induced hypoglycemia on plasma oxytocin levels in bulimia and anorexia nervosa. Metab. Clin. Exp. 40, 1226–1230 10.1016/0026-0495(91)90220-Q 1943752

[B61] Chow K. B. Sun J. Chu K. M. Tai Cheung W. Cheng C. H. Wise H. (2012). The truncated ghrelin receptor polypeptide (GHS-R1b) is localized in the endoplasmic reticulum where it forms heterodimers with ghrelin receptors (GHS-R1a) to attenuate their cell surface expression. Mol. Cell. Endocrinol. 348, 247–254 10.1016/j.mce.2011.08.034 21903149

[B62] Chuang J. C. Zigman J. M. (2010). Ghrelin’s roles in stress, mood, and anxiety regulation. Int. J. Pept. 2010, pii: 460549. 10.1155/2010/460549 PMC2915752 20721341

[B63] Ciofi P. (2011). The arcuate nucleus as a circumventricular organ in the mouse. Neurosci. Lett. 487, 187–190 10.1016/j.neulet.2010.10.019 20951768

[B64] Ciofi P. Garret M. Lapirot O. Lafon P. Loyens A. Prevot V. (2009). Brain-endocrine interactions: a microvascular route in the mediobasal hypothalamus. Endocrinology 150, 5509–5519 10.1210/en.2009-0584 19837874 PMC2819742

[B65] Confavreux C. B. Levine R. L. Karsenty G. (2009). A paradigm of integrative physiology, the crosstalk between bone and energy balances. Mol. Cell. Endocrinol. 310, 21–29 10.1016/j.mce.2009.04.004 19376193 PMC3667507

[B66] Corcos M. Guilbaud O. Paterniti S. Moussa M. Chambry J. Chaouat G. (2003). Involvement of cytokines in eating disorders: a critical review of the human literature. Psychoneuroendocrinology 28, 229–249 10.1016/S0306-4530(02)00021-5 12573293

[B67] Corwin R. L. Avena N. M. Boggiano M. M. (2011). Feeding and reward: perspectives from three rat models of binge eating. Physiol. Behav. 104, 87–97 10.1016/j.physbeh.2011.04.041 21549136 PMC3132131

[B68] Costantini V. J. Vicentini E. Sabbatini F. M. Valerio E. Lepore S. Tessari M. (2011). GSK1614343, a novel ghrelin receptor antagonist, produces an unexpected increase of food intake and body weight in rodents and dogs. Neuroendocrinology 94, 158–168 10.1159/000328968 21778696

[B69] Cowley M. A. Smith R. G. Diano S. Tschöp M. Pronchuk N. Grove K. L. (2003). The distribution and mechanism of action of ghrelin in the CNS demonstrates a novel hypothalamic circuit regulating energy homeostasis. Neuron 37, 649–661 10.1016/S0896-6273(03)00063-1 12597862

[B70] Cummings D. E. Purnell J. Q. Frayo R. S. Schmidova K. Wisse B. E. Weigle D. S. (2001). A preprandial rise in plasma ghrelin levels suggests a role in meal initiation in humans. Diabetes 50, 1714–1719 10.2337/diabetes.50.8.1714 11473029

[B71] Date Y. Murakami N. Toshinai K. Matsukura S. Niijima A. Matsuo H. (2002). The role of the gastric afferent vagal nerve in ghrelin-induced feeding and growth hormone secretion in rats. Gastroenterology 123, 1120–1128 10.1053/gast.2002.35954 12360474

[B72] Davis C. Claridge G. (1998). The eating disorders as addiction: a psychobiological perpspective. Addict. Behav. 23, 463–475 10.1016/S0306-4603(98)00009-4 9698975

[B73] Davis C. Katzman D. K. Kaptein S. Kirsh C. Brewer H. Kalmbach K. (1997). The prevalence of high-level exercise in the eating disorders: etiological implications. Compr. Psychiatry 38, 321–326 10.1016/S0010-440X(97)90927-5 9406737

[B74] Delhanty P. J. Neggers S. J. Van der Lely A. J. (2012). Ghrelin: the differences between Acyl- and Des-acyl Ghrelin. Eur. J. Endocrinol. 167, 601–608 10.1530/EJE-12-0456 22898499

[B75] Delhanty P. J. van der Eerden B. C. van der Velde M. Gauna C. Pols H. A. Jahr H. (2006). Ghrelin and unacylated ghrelin stimulate human osteoblast growth via mitogen-activated protein kinase (MAPK)/phosphoinositide 3-kinase (PI3K) pathways in the absence of GHS-R1a. J. Endocrinol. 188, 37–47 10.1677/joe.1.06404 16394173

[B76] Delporte M. L. Brichard S. M. Hermans M. P. Beguin C. Lambert M. (2003). Hyperadiponectinaemia in anorexia nervosa. Clin. Endocrinol. (Oxf.) 58, 22–29 10.1046/j.1365-2265.2003.01702.x 12519408

[B77] Demitrack M. A. Lesem M. D. Listwak S. J. Brandt H. A. Jimerson D. C. Gold P. W. (1990). CSF oxytocin in anorexia nervosa and bulimia nervosa: clinical and pathophysiologic considerations. Am. J. Psychiatry 147, 882–886 2356873 10.1176/ajp.147.7.882

[B78] Di Carlo C. Tommaselli G. A. De Filippo E. Pisano G. Nasti A. Bifulco G. (2002). Menstrual status and serum leptin levels in anorectic and in menstruating women with low body mass indexes. Fertil. Steril. 78, 376–382 10.1016/S0015-0282(02)03221-1 12137877

[B79] Dickson S. L. Doutrelant-Viltart O. Dyball R. E. Leng G. (1996). Retrogradely labelled neurosecretory neurons of the rat hypothalamic arcuate nucleus express Fos protein following systemic injection of GH-releasing peptide-6. J. Endocrinol. 151, 323–331 10.1677/joe.0.1510323 8958794

[B80] Dickson S. L. Leng G. Robinson I. C. (1993). Systemic administration of growth hormone-releasing peptide activates hypothalamic arcuate neurons. Neuroscience 53, 303–306 10.1016/0306-4522(93)90197-N 8492908

[B81] Dickson S. L. Luckman S. M. (1997). Induction of c-fos messenger ribonucleic acid in neuropeptide Y and growth hormone (GH)-releasing factor neurons in the rat arcuate nucleus following systemic injection of the GH secretagogue, GH-releasing peptide-6. Endocrinology 138, 771–777 10.1210/en.138.2.771 9003014

[B82] Djurovic M. Pekic S. Petakov M. Damjanovic S. Doknic M. Dieguez C. (2004). Gonadotropin response to clomiphene and plasma leptin levels in weight recovered but amenorrhoeic patients with anorexia nervosa. J. Endocrinol. Invest. 27, 523–527 15717648 10.1007/BF03347473

[B83] Dolezalova R. Lacinova Z. Dolinkova M. Kleiblova P. Haluzikova D. Housa D. (2007). Changes of endocrine function of adipose tissue in anorexia nervosa: comparison of circulating levels versus subcutaneous mRNA expression. Clin. Endocrinol. (Oxf.) 67, 674–678 10.1111/j.1365-2265.2007.02944.x 17953628

[B84] Dominguez J. Goodman L. Sen Gupta S. Mayer L. Etu S. F. Walsh B. T. (2007). Treatment of anorexia nervosa is associated with increases in bone mineral density, and recovery is a biphasic process involving both nutrition and return of menses. Am. J. Clin. Nutr. 86, 92–99 17616767 10.1093/ajcn/86.1.92

[B85] Dostalova I. Kopsky V. Duskova J. Papezova H. Pacak K. Nedvidkova J. (2005). Leptin concentrations in the abdominal subcutaneous adipose tissue of patients with anorexia nervosa assessed by in vivo microdialysis. Regul. Pept. 128, 63–68 10.1016/j.regpep.2004.12.029 15721489

[B86] Dostalova I. Smitka K. Papezova H. Kvasnickova H. Nedvidkova J. (2007). Increased insulin sensitivity in patients with anorexia nervosa: the role of adipocytokines. Physiol. Res. 56, 587–594 17184143 10.33549/physiolres.931089

[B87] Eddy K. T. Dorer D. J. Franko D. L. Tahilani K. Thompson-Brenner H. Herzog D. B. (2008). Diagnostic crossover in anorexia nervosa and bulimia nervosa: implications for DSM-V. Am. J. Psychiatry 165, 245–250 10.1176/appi.ajp.2007.07060951 18198267 PMC3684068

[B88] Egecioglu E. Jerlhag E. Salomé N. Skibicka K. P. Haage D. Bohlooly Y. M. (2010). Ghrelin increases intake of rewarding food in rodents. Addict. Biol. 15, 304–311 10.1111/j.1369-1600.2010.00216.x 20477752 PMC2901520

[B89] Ehrlich S. Salbach-Andrae H. Eckart S. Merle J. V. Burghardt R. Pfeiffer E. (2009). Serum brain-derived neurotrophic factor and peripheral indicators of the serotonin system in underweight and weight-recovered adolescent girls and women with anorexia nervosa. J. Psychiatry Neurosci. 34, 323–329 19568484 PMC2702450

[B90] Enomoto M. Nagaya N. Uematsu M. Okumura H. Nakagawa E. Ono F. (2003). Cardiovascular and hormonal effects of subcutaneous administration of ghrelin, a novel growth hormone-releasing peptide, in healthy humans. Clin. Sci. (Lond.) 105, 431–435 10.1042/CS20030184 12773196

[B91] Erdur L. Kallenbach-Dermutz B. Lehmann V. Zimmermann-Viehoff F. Köpp W. Weber C. (2012). Somatic comorbidity in anorexia nervosa: first results of a 21-year follow-up study on female inpatients. Biopsychosoc. Med. 6, 4 10.1186/1751-0759-6-4 22300749 PMC3299644

[B92] España R. A. Scammell T. E. (2011). Sleep neurobiology from a clinical perspective. Sleep 34, 845–858 21731134 10.5665/SLEEP.1112 PMC3119826

[B93] Estour B. Germain N. Diconne E. Frere D. Cottet-Emard J. M. Carrot G. (2010). Hormonal profile heterogeneity and short-term physical risk in restrictive anorexia nervosa. J. Clin. Endocrinol. Metab. 95, 2203–2210 10.1210/jc.2009-2608 20305007

[B94] Faje A. T. Fazeli P. K. Katzman D. K. Miller K. K. Breggia A. Rosen C. J. (2012). Sclerostin levels and bone turnover markers in adolescents with anorexia nervosa and healthy adolescent girls. Bone 51, 474–479 10.1016/j.bone.2012.06.006 22728230 PMC3412913

[B95] Faulconbridge L. F. Cummings D. E. Kaplan J. M. Grill H. J. (2003). Hyperphagic effects of brainstem ghrelin administration. Diabetes 52, 2260–2265 10.2337/diabetes.52.9.2260 12941764

[B96] Fazeli P. K. Lawson E. A. Prabhakaran R. Miller K. K. Donoho D. A. Clemmons D. R. (2010a). Effects of recombinant human growth hormone in anorexia nervosa: a randomized, placebo-controlled study. J. Clin. Endocrinol. Metab. 95, 4889–4897 10.1210/jc.2009-1730 20668047 PMC2968733

[B97] Fazeli P. K. Misra M. Goldstein M. Miller K. K. Klibanski A. (2010b). Fibroblast growth factor-21 may mediate growth hormone resistance in anorexia nervosa. J. Clin. Endocrinol. Metab. 95, 369–374 10.1210/jc.2009-1730 19926712 PMC2805486

[B98] Fernández-Fernández R. Tena-Sempere M. Navarro V. M. Barreiro M. L. Castellano J. M. Aguilar E. (2005). Effects of ghrelin upon gonadotropin-releasing hormone and gonadotropin secretion in adult female rats: in vivo and in vitro studies. Neuroendocrinology 82, 245–255 10.1159/000092753 16721030

[B99] Ferron F. Considine R. V. Peino R. Lado I. G. Dieguez C. Casanueva F. F. (1997). Serum leptin concentrations in patients with anorexia nervosa, bulimia nervosa and nonspecific eating disorders correlate with the body mass index but are independent of the respective disease. Clin. Endocrinol. (Oxf.) 46, 289–293 10.1046/j.1365-2265.1997.1260938.x 9156037

[B100] Fisher M. (2003). The course and outcome of eating disorders in adults and in adolescents: a review. Adolesc. Med. 14, 149–158 12529198

[B101] Forbes S. Li X. F. Kinsey-Jones J. O’Byrne K. (2009). Effects of ghrelin on Kisspeptin mRNA expression in the hypothalamic medial preoptic area and pulsatile luteinising hormone secretion in the female rat. Neurosci. Lett. 460, 143–147 10.1016/j.neulet.2009.05.060 19477231

[B102] Fujimoto S. Inui A. Kiyota N. Seki W. Koide K. Takamiya S. (1997). Increased cholecystokinin and pancreatic polypeptide responses to a fat-rich meal in patients with restrictive but not bulimic anorexia nervosa. Biol. Psychiatry 41, 1068–1070 10.1016/S0006-3223(97)00044-9 9129788

[B103] Fujitsuka N. Asakawa A. Amitani H. Fujimiya M. Inui A. (2012). Ghrelin and gastrointestinal movement. Meth. Enzymol. 514, 289–301 22975060 10.1016/B978-0-12-381272-8.00018-0

[B104] Fukushima N. Hanada R. Teranishi H. Fukue Y. Tachibana T. Ishikawa H. (2005). Ghrelin directly regulates bone formation. J. Bone Miner. Res. 20, 790–798 10.1359/JBMR.041237 15824852

[B105] Galusca B. Bossu C. Germain N. Kadem M. Frere D. Lafage-Proust M. H. (2006). Age-related differences in hormonal and nutritional impact on lean anorexia nervosa bone turnover uncoupling. Osteoporos. Int. 17, 888–896 10.1007/s00198-005-0063-0 16541206

[B106] Galusca B. Jeandel L. Germain N. Alexandre D. Leprince J. Anouar Y. (2012). Orexigenic neuropeptide 26RFa: new evidence for an adaptive profile of appetite regulation in anorexia nervosa. J. Clin. Endocrinol. Metab. 97, 2012–2018 10.1210/jc.2011-3396 22466335

[B107] Gao X. B. (2012). Plasticity in neurons synthesizing wake/arousal promoting hormone hypocretin/orexin. Vitam. Horm. 89, 35–59 10.1016/B978-0-12-394623-2.00003-2 22640607

[B108] Garcia F. D. Délavenne H. Déchelotte P. (2011). Atypical eating disorders a review. Nutr. Diet. Suppl. 3, 67–75

[B109] Gauna C. Delhanty P. J. Hofland L. J. Janssen J. A. Broglio F. Ross R. J. (2005). Ghrelin stimulates, whereas des-octanoyl ghrelin inhibits, glucose output by primary hepatocytes. J. Clin. Endocrinol. Metab. 90, 1055–1060 10.1210/jc.2004-1069 15536157

[B110] Gelegen C. Collier D. A. Campbell I. C. Oppelaar H. van den Heuvel J. Adan R. A. (2007). Difference in susceptibility to activity-based anorexia in two inbred strains of mice. Eur. Neuropsychopharmacol. 17, 199–205 10.1016/S0924-977X(07)70234-1 16735105

[B111] Gendall K. A. Kaye W. H. Altemus M. McConaha C. W. La Via M. C. (1999). Leptin, neuropeptide Y, and peptide YY in long-term recovered eating disorder patients. Biol. Psychiatry 46, 292–299 10.1016/S0006-3223(98)00292-3 10418705

[B112] Geracioti T. D. Jr. Liddle R. A. Altemus M. Demitrack M. A. Gold P. W. (1992). Regulation of appetite and cholecystokinin secretion in anorexia nervosa. Am. J. Psychiatry 149, 958–961 1609878 10.1176/ajp.149.7.958

[B113] Germain N. Galusca B. Grouselle D. Frere D. Billard S. Epelbaum J. (2010). Ghrelin and obestatin circadian levels differentiate bingeing–purging from restrictive anorexia nervosa. J. Clin. Endocrinol. Metab. 95, 3057–3062 10.1210/jc.2009-2196 20339027

[B114] Germain N. Galusca B. Grouselle D. Frere D. Tolle V. Zizzari P. (2009). Ghrelin/obestatin ratio in two populations with low bodyweight: constitutional thinness and anorexia nervosa. Psychoneuroendocrinology 34, 413–419 10.1016/j.psyneuen.2008.10.001 18995969

[B115] Germain N. Galusca B. Le Roux C. W. Bossu C. Ghatei M. A. Lang F. (2007). Constitutional thinness and lean anorexia nervosa display opposite concentrations of peptide YY, glucagon-like peptide 1, ghrelin, and leptin. Am. J. Clin. Nutr. 85, 967–971 17413094 10.1093/ajcn/85.4.967

[B116] Gerner R. H. Gwirtsman H. E. (1981). Abnormalities of dexamethasone suppression test and urinary MHPG in anorexia nervosa. Am. J. Psychiatry 138, 650–653 7235062 10.1176/ajp.138.5.650

[B117] Gerner R. H. Sharp B. (1982a). CSF beta-endorphin-immunoreactivity in normal, schizophrenic, depressed, manic and anorexic subjects. Brain Res. 237, 244–247 10.1016/0006-8993(82)90574-1 6280804

[B118] Gerner R. H. Yamada T. (1982b). Altered neuropeptide concentrations in cerebrospinal fluid of psychiatric patients. Brain Res. 238, 298–302 10.1016/0006-8993(82)90801-0 6123376

[B119] Gianotti L. Pincelli A. I. Scacchi M. Rolla M. Bellitti D. Arvat E. (2000). Effects of recombinant human insulin-like growth factor I administration on spontaneous and growth hormone (GH)-releasing hormone-stimulated GH secretion in anorexia nervosa. J. Clin. Endocrinol. Metab. 85, 2805–2809 10.1210/jc.85.10.3604 10946886

[B120] Gnanapavan S. Kola B. Bustin S. A. Morris D. G. McGee P. Fairclough P. (2002). The tissue distribution of the mRNA of ghrelin and subtypes of its receptor, GHS-R, in humans. J. Clin. Endocrinol. Metab. 87, 2988 10.1210/jc.87.6.2988 12050285

[B121] Gniuli D. Liverani E. Capristo E. Greco A. V. Mingrone G. (2001). Blunted glucose metabolism in anorexia nervosa. Metab. Clin. Exp. 50, 876–881 10.1053/meta.2001.24915 11474473

[B122] González C. R. Vázquez M. J. López M. Diéguez C. (2008). Influence of chronic undernutrition and leptin on GOAT mRNA levels in rat stomach mucosa. J. Mol. Endocrinol. 41, 415–421 10.1677/JME-08-0102 18835978

[B123] Goodman A. (1990). Addiction: definition and implications. Br. J. Addict. 85, 1403–1408 10.1111/j.1360-0443.1990.tb01620.x 2285834

[B124] Goodyear S. Arasaradnam R. P. Quraishi N. Mottershead M. Nwokolo C. U. (2010). Acylated and des acyl ghrelin in human portal and systemic circulations. Mol. Biol. Rep. 37, 3697–3701 10.1007/s11033-010-0022-5 20195769

[B125] Gordon C. M. Goodman E. Emans S. J. Grace E. Becker K. A. Rosen C. J. (2002). Physiologic regulators of bone turnover in young women with anorexia nervosa. J. Pediatr. 141, 64–70 10.1067/mpd.2002.125003 12091853

[B126] Gourcerol G. St-Pierre D. H. Tache Y. (2007). Lack of obestatin effects on food intake: should obestatin be renamed ghrelin-associated peptide (GAP)? Regul. Pept. 141, 1–7 10.1016/j.regpep.2006.12.023 17321609

[B127] Grinspoon S. Miller K. Coyle C. Krempin J. Armstrong C. Pitts S. (1999). Severity of osteopenia in estrogen-deficient women with anorexia nervosa and hypothalamic amenorrhea. J. Clin. Endocrinol. Metab. 84, 2049–2055 10.1210/jc.84.6.2049 10372709

[B128] Grinspoon S. Thomas E. Pitts S. Gross E. Mickley D. Miller K. (2000). Prevalence and predictive factors for regional osteopenia in women with anorexia nervosa. Ann. Intern. Med. 133, 790–794 11085841 10.7326/0003-4819-133-10-200011210-00011 PMC3206091

[B129] Grove K. L. Cowley M. A. (2005). Is ghrelin a signal for the development of metabolic systems? J. Clin. Invest. 115, 3393–3397 10.1172/JCI27211 16322785 PMC1297272

[B130] Guan X. M. Yu H. Palyha O. C. McKee K. K. Feighner S. D. Sirinathsinghji D. J. (1997). Distribution of mRNA encoding the growth hormone secretagogue receptor in brain and peripheral tissues. Brain Res. Mol. Brain Res. 48, 23–29 10.1016/S0169-328X(97)00071-5 9379845

[B131] Gutierrez J. A. Solenberg P. J. Perkins D. R. Willency J. A. Knierman M. D. Jin Z. (2008). Ghrelin octanoylation mediated by an orphan lipid transferase. Proc. Natl. Acad. Sci. U.S.A. 105, 6320–6325 10.1073/pnas.0800708105 18443287 PMC2359796

[B132] Haas V. Onur S. Paul T. Nutzinger D. O. Bosy-Westphal A. Hauer M. (2005). Leptin and body weight regulation in patients with anorexia nervosa before and during weight recovery. Am. J. Clin. Nutr. 81, 889–896 15817868 10.1093/ajcn/81.4.889

[B133] Haas V. K. Kohn M. R. Clarke S. D. Allen J. R. Madden S. Müller M. J. (2009). Body composition changes in female adolescents with anorexia nervosa. Am. J. Clin. Nutr. 89, 1005–1010 10.3945/ajcn.2008.26958 19211813

[B134] Hall J. F. Hanford P. V. (1954). Activity as a function of a restricted feeding schedule. J. Comp. Physiol. Psychol. 47, 362–373 10.1037/h0060276 13221681

[B135] Haluzíková D. Dostálová I. Kaválková P. Roubícek T. Mráz M. Papezová H. (2009). Serum concentrations of adipocyte fatty acid binding protein in patients with anorexia nervosa. Physiol. Res. 58, 577–581 18657008 10.33549/physiolres.931575

[B136] Hansson C. Haage D. Taube M. Egecioglu E. Salomé N. Dickson S. L. (2011). Central administration of ghrelin alters emotional responses in rats: behavioural, electrophysiological and molecular evidence. Neuroscience 180, 201–211 10.1016/j.neuroscience.2011.02.002 21303683

[B137] Harada T. Nakahara T. Yasuhara D. Kojima S. Sagiyama K. Amitani H. (2008). Obestatin, acyl ghrelin, and des-acyl ghrelin responses to an oral glucose tolerance test in the restricting type of anorexia nervosa. Biol. Psychiatry 63, 245–247 10.1016/j.biopsych.2007.04.005 17706613

[B138] Harrold J. A. Dovey T. Cai X. J. Halford J. C. Pinkney J. (2008). Autoradiographic analysis of ghrelin receptors in the rat hypothalamus. Brain Res. 1196, 59–64 10.1016/j.brainres.2007.12.055 18234162

[B139] Hasan T. F. Hasan H. (2011). Anorexia nervosa: a unified neurological perspective. Int. J. Med. Sci. 8, 679–703 22135615 10.7150/ijms.8.679 PMC3204438

[B377] Hassouna R. Labarthe A. Zizzari P. Videau C. Culler M. D. Epelbaum J. (2013). Actions of agonists and antagonists of the ghrelin/GHSR pathway onGHsecretion, appetite and cFos activity. Front. Endocrinol. 4:25 10.3389/fendo.2013.00025 PMC3600614 23515849

[B140] Hassouna R. Zizzari P. Tolle V. (2010). The ghrelin/obestatin balance in the physiological and pathological control of growth hormone secretion, body composition and food intake. J. Neuroendocrinol. 22, 793–804 20456603 10.1111/j.1365-2826.2010.02019.x

[B141] Hataya Y. Akamizu T. Takaya K. Kanamoto N. Ariyasu H. Saijo M. (2001). A low dose of ghrelin stimulates growth hormone (GH) release synergistically with GH-releasing hormone in humans. J. Clin. Endocrinol. Metab. 86, 4552 10.1210/jc.86.9.4552 11549707

[B142] Hebebrand J. Blum W. F. Barth N. Coners H. Englaro P. Juul A. (1997). Leptin levels in patients with anorexia nervosa are reduced in the acute stage and elevated upon short-term weight restoration. Mol. Psychiatry 2, 330–334 10.1038/sj.mp.4000282 9246674

[B143] Heer M. Mika C. Grzella I. Heussen N. Herpertz-Dahlmann B. (2004). Bone turnover during inpatient nutritional therapy and outpatient follow-up in patients with anorexia nervosa compared with that in healthy control subjects. Am. J. Clin. Nutr. 80, 774–781 15321821 10.1093/ajcn/80.3.774

[B144] Hewson A. K. Dickson S. L. (2000). Systemic administration of ghrelin induces Fos and Egr-1 proteins in the hypothalamic arcuate nucleus of fasted and fed rats. J. Neuroendocrinol. 12, 1047–1049 10.1046/j.1365-2826.2000.00584.x 11069119

[B145] Hewson A. K. Viltart O. McKenzie D. N. Dyball R. E. Dickson S. L. (1999). GHRP-6-induced changes in electrical activity of single cells in the arcuate, ventromedial and periventricular nucleus neurons [correction of nuclei] of a hypothalamic slice preparation in vitro. J. Neuroendocrinol. 11, 919–923 [Erratum in: *J. Neuroendocrinol* (2000) 12, 377]. 10.1046/j.1365-2826.1999.00408.x 10583726

[B146] Holst B. Holliday N. D. Bach A. Elling C. E. Cox H. M. Schwartz T. W. (2004). Common structural basis for constitutive activity of the ghrelin receptor family. J. Biol. Chem. 279, 53806–53817 10.1074/jbc.M407676200 15383539

[B147] Holst B. Schwartz T. W. (2006). Ghrelin receptor mutations – too little height and too much hunger. J. Clin. Invest. 116, 637–641 10.1172/JCI27999 16511600 PMC1386123

[B148] Holtkamp K. Hebebrand J. Mika C. Grzella I. Heer M. Heussen N. (2003a). The effect of therapeutically induced weight gain on plasma leptin levels in patients with anorexia nervosa. J. Psychiatr. Res. 37, 165–169 10.1016/S0022-3956(02)00100-0 12842170

[B149] Holtkamp K. Mika C. Grzella I. Heer M. Pak H. Hebebrand J. (2003b). Reproductive function during weight gain in anorexia nervosa. Leptin represents a metabolic gate to gonadotropin secretion. J. Neural Transm. 110, 427–435 10.1007/s00702-002-0800-x 12658369

[B150] Holtkamp K. Hebebrand J. Mika C. Heer M. Heussen N. Herpertz-Dahlmann B. (2004). High serum leptin levels subsequent to weight gain predict renewed weight loss in patients with anorexia nervosa. Psychoneuroendocrinology 29, 791–797 10.1016/S0306-4530(03)00143-4 15110928

[B151] Hotta M. Ohwada R. Akamizu T. Shibasaki T. Takano K. Kangawa K. (2009). Ghrelin increases hunger and food intake in patients with restricting-type anorexia nervosa: a pilot study. Endocr. J. 56, 1119–1128 10.1507/endocrj.K09E-168 19755753

[B152] Hotta M. Ohwada R. Katakami H. Shibasaki T. Hizuka N. Takano K. (2004). Plasma levels of intact and degraded ghrelin and their responses to glucose infusion in anorexia nervosa. J. Clin. Endocrinol. Metab. 89, 5707–5712 10.1210/jc.2004-0353 15531532

[B153] Hotta M. Shibasaki T. Masuda A. Imaki T. Demura H. Ling N. (1986). The responses of plasma adrenocorticotropin and cortisol to corticotropin-releasing hormone (CRH) and cerebrospinal fluid immunoreactive CRH in anorexia nervosa patients. J. Clin. Endocrinol. Metab. 62, 319–324 10.1210/jcem-62-2-319 3001125

[B154] Housova J. Anderlova K. Krizova J. Haluzikova D. Kremen J. Kumstyrova T. (2005). Serum adiponectin and resistin concentrations in patients with restrictive and binge/purge form of anorexia nervosa and bulimia nervosa. J. Clin. Endocrinol. Metab. 90, 1366–1370 10.1210/jc.2004-1364 15598689

[B155] Howard A. D. Feighner S. D. Cully D. F. Arena J. P. Liberator P. A. Rosenblum C. I. (1996). A receptor in pituitary and hypothalamus that functions in growth hormone release. Science 273, 974–977 10.1126/science.273.5277.974 8688086

[B156] Howgate D. J. Graham S. M. Leonidou A. Korres N. Tsiridis E. Tsapakis E. (2013). Bone metabolism in anorexia nervosa: molecular pathways and current treatment modalities. Osteoporos. Int. 24, 407–421 10.1007/s00198-012-2095-6 22875459

[B157] Iwahashi H. Funahashi T. Kurokawa N. Sayama K. Fukuda E. Okita K. (2003). Plasma adiponectin levels in women with anorexia nervosa. Horm. Metab. Res. 35, 537–540 10.1055/s-2003-42655 14517770

[B158] Jacquemont S. Reymond A. Zufferey F. Harewood L. Walters R. G. Kutalik Z. (2011). Mirror extreme BMI phenotypes associated with gene dosage at the chromosome 16p11.2 locus. Nature 478, 97–102 10.1038/nature10406 21881559 PMC3637175

[B159] Janas-Kozik M. Krupka-Matuszczyk I. Malinowska-Kolodziej I. Lewin-Kowalik J. (2007). Total ghrelin plasma level in patients with the restrictive type of anorexia nervosa. Regul. Pept. 140, 43–46 10.1016/j.regpep.2006.11.005 17187877

[B160] Jáuregui-Garrido B. Jáuregui-Lobera I. (2012). Sudden death in eating disorders. Vasc. Health Risk Manag. 8, 91–98 22393299 10.2147/VHRM.S28652 PMC3292410

[B161] Jeffery P. L. Duncan R. P. Yeh A. H. Jaskolski R. A. Hammond D. S. Herington A. C. (2005). Expression of the ghrelin axis in the mouse: an exon 4-deleted mouse proghrelin variant encodes a novel C terminal peptide. Endocrinology 146, 432–440 10.1210/en.2003-1466 15471962

[B162] Jerlhag E. Egecioglu E. Dickson S. L. Andersson M. Svensson L. Engel J. A. (2006). Ghrelin stimulates locomotor activity and accumbal dopamine-overflow via central cholinergic systems in mice: implications for its involvement in brain reward. Addict. Biol. 11, 45–54 10.1111/j.1369-1600.2006.00002.x 16759336

[B163] Jerlhag E. Egecioglu E. Dickson S. L. Douhan A. Svensson L. Engel J. A. (2007). Ghrelin administration into tegmental areas stimulates locomotor activity and increases extracellular concentration of dopamine in the nucleus accumbens. Addict. Biol. 12, 6–16 10.1111/j.1369-1600.2006.00041.x 17407492

[B164] Jerlhag E. Egecioglu E. Dickson S. L. Engel J. A. (2010). Ghrelin receptor antagonism attenuates cocaine- and amphetamine-induced locomotor stimulation, accumbal dopamine release, and conditioned place preference. Psychopharmacology (Berl.) 211, 415–422 10.1007/s00213-010-1907-7 20559820 PMC2908453

[B165] Jerlhag E. Egecioglu E. Landgren S. Salomé N. Heilig M. Moechars D. (2009). Requirement of central ghrelin signaling for alcohol reward. Proc. Natl. Acad. Sci. U.S.A. 106, 11318–11323 10.1073/pnas.0812809106 19564604 PMC2703665

[B166] Jiang H. Betancourt L. Smith R. G. (2006). Ghrelin amplifies dopamine signaling by cross talk involving formation of growth hormone secretagogue receptor/dopamine receptor subtype 1 heterodimers. Mol. Endocrinol. 20, 1772–1785 10.1210/me.2005-0084 16601073

[B167] Kamegai J. Tamura H. Shimizu T. Ishii S. Sugihara H. Wakabayashi I. (2000). Central effect of ghrelin, an endogenous growth hormone secretagogue, on hypothalamic peptide gene expression. Endocrinology 141, 4797–4780 10.1210/en.141.12.4797 11108296

[B168] Kamegai J. Tamura H. Shimizu T. Ishii S. Sugihara H. Wakabayashi I. (2001). Chronic central infusion of ghrelin increases hypothalamic neuropeptide Y and agouti-related protein mRNA levels and body weight in rats. Diabetes 50, 2438–2443 10.2337/diabetes.50.11.2438 11679419

[B169] Kang K. Schmahl J. Lee J. M. Garcia K. Patil K. Chen A. (2012). Mouse ghrelin-O-acyltransferase (GOAT) plays a critical role in bile acid reabsorption. FASEB J. 26, 259–271 10.1096/fj.11-202853 21965605

[B170] Karczewska-Kupczewska M. Kowalska I. Nikolajuk A. Adamska A. Otziomek E. Gorska M. (2012). Hyperinsulinemia acutely increases serum macrophage inhibitory cytokine-1 concentration in anorexia nervosa and obesity. Clin. Endocrinol. (Oxf.) 76, 46–50 10.1111/j.1365-2265.2011.04139.x 21645023

[B171] Karczewska-Kupczewska M. Straczkowski M. Adamska A. Nikolajuk A. Otziomek E. Górska M. (2010). Insulin sensitivity, metabolic flexibility, and serum adiponectin concentration in women with anorexia nervosa. Metab. Clin. Exp. 59, 473–477 10.1016/j.metabol.2009.07.036 19846178

[B172] Kawahara Y. Kawahara H. Kaneko F. Yamada M. Nishi Y. Tanaka E. (2009). Peripherally administered ghrelin induces bimodal effects on the mesolimbic dopamine system depending on food-consumptive states. Neuroscience 161, 855–864 10.1016/j.neuroscience.2009.03.086 19362120

[B173] Kaye W. H. (1996). Neuropeptide abnormalities in anorexia nervosa. Psychiatry Res. 62, 65–74 10.1016/0165-1781(96)02985-X 8739116

[B174] Kaye W. H. Berrettini W. Gwirtsman H. George D. T. (1990). Altered cerebrospinal fluid neuropeptide Y and peptide YY immunoreactivity in anorexia and bulimia nervosa. Arch. Gen. Psychiatry 47, 548–556 10.1001/archpsyc.1990.01810180048008 2350207

[B175] Kaye W. H. Fudge J. L. Paulus M. (2009). New insights into symptoms and neuro-circuit function of anorexia nervosa. Nat. Rev. Neurosci. 10, 573–584 10.1038/nrn2682 19603056 PMC13038070

[B176] Kaye W. H. Gwirtsman H. E. George D. T. Ebert M. H. Jimerson D. C. Tomai T. P. (1987). Elevated cerebrospinal fluid levels of immunoreactive corticotrophin-releasing hormone in anorexia nervosa: relation to state of nutrition, adrenal function and intensity of depression. J. Clin. Endocrinol. Metab. 64, 203–208 10.1210/jcem-64-2-203 3491830

[B177] Kaye W. H. Rubinow D. Gwirtsman H. E. George D. T. Jimerson D. C. Gold P. W. (1988). CSF somatostatin in anorexia nervosa and bulimia: relationship to the hypothalamic pituitary-adrenal cortical axis. Psychoneuroendocrinology 13, 265–272 10.1016/0306-4530(88)90024-8 2900533

[B178] Kern A. Albarran-Zeckler R. Walsh H. E. Smith R. G. (2012). Apo-ghrelin receptor forms heteromers with DRD2 in hypothalamic neurons and is essential for anorexigenic effects of DRD2 agonism. Neuron 73, 317–332 10.1016/j.neuron.2011.10.038 22284186 PMC3269786

[B179] Kim S. F. (2012). Animal models of eating disorders. Neuroscience 211, 2–12 10.1016/j.neuroscience.2012.03.024 22465439 PMC3351502

[B180] Kinzig K. P. Coughlin J. W. Redgrave G. W. Moran T. H. Guarda A. S. (2007). Insulin, glucose, and pancreatic polypeptide responses to a test meal in restricting type anorexia nervosa before and after weight restoration. Am. J. Physiol. Endocrinol. Metab. 292, E1441–E1446 10.1152/ajpendo.00347.2006 17264227

[B181] Kleinz M. J. Maguire J. J. Skepper J. N. Davenport A. P. (2006). Functional and immunocytochemical evidence for a role of ghrelin and des-octanoyl ghrelin in the regulation of vascular tone in man. Cardiovasc. Res. 69, 227–235 10.1016/j.cardiores.2005.09.001 16226234

[B182] Klenotich S. J. Dulawa S. C. (2012). The activity-based anorexia mouse model. Methods Mol. Biol. 829, 377–393 10.1007/978-1-61779-458-2_25 22231828

[B183] Kluge M. Schüssler P. Schmidt D. Uhr M. Steiger A. (2012). Ghrelin suppresses secretion of luteinizing hormone (LH) and follicle-stimulating hormone (FSH) in women. J. Clin. Endocrinol. Metab. 97, E448–E451 10.1210/jc.2011-2607 22259063

[B184] Kohl M. Foulon C. Guelfi J. D. (2004). Hyperactivity and anorexia nervosa: behavioural and biological perspective. Encephale 30, 492–499 10.1016/S0013-7006(04)95463-2 15627053

[B185] Kojima M. Hosoda H. Date Y. Nakazato M. Matsuo H. Kangawa K. (1999). Ghrelin is a growth-hormone-releasing acylated peptide from stomach. Nature 402, 656–660 10.1038/45230 10604470

[B186] Kojima M. Kangawa K. (2005). Ghrelin: structure and function. Physiol. Rev. 85, 495–522 10.1152/physrev.00012.2004 15788704

[B187] Krizova J. Papezova H. Haluzikova D. Parizkova J. Jiskra J. Kotrlikova E. (2002). Soluble leptin receptor levels in patients with anorexia nervosa. Endocr. Res. 28, 199–205 10.1081/ERC-120015058 12489569

[B188] Krsek M. Rosicka M. Papezova H. Krizova J. Kotrikova E. Haluzk M. (2003). Plasma ghrelin levels and malnutrition: a comparison of two etiologies. Eat. Weight Disord. 8, 207–211 14649784 10.1007/BF03325015

[B189] Kukkonen J. P. (2013). Physiology of the orexinergic/hypocretinergic system: a revisit in 2012. Am. J. Physiol. Cell Physiol. 304, C2–C32 10.1152/ajpcell.00227.2012 23034387

[B190] Lamont E. W. Patterson Z. Rodrigues T. Vallejos O. Blum I. D. Abizaid A. (2012). Ghrelin-deficient mice have fewer orexin cells and reduced cFOS expression in the mesolimbic dopamine pathway under a restricted feeding paradigm. Neuroscience 218, 12–19 10.1016/j.neuroscience.2012.05.046 22641088

[B191] Lauer C. J. Krieg J. C. (2004). Sleep in eating disorders. Sleep Med. Rev. 8, 109–118 10.1016/S1087-0792(02)00122-3 15033150

[B192] Lawrence C. B. Snape A. C. Baudoin F. M. Luckman S. M. (2002). Acute central ghrelin and GH secretagogues induce feeding and activate brain appetite centers. Endocrinology 143, 155–162 10.1210/en.143.2.360 11751604

[B193] Lawson E. A. Donoho D. Miller K. K. Misra M. Meenaghan E. Lydecker J. (2009). Hypercortisolemia is associated with severity of bone loss and depression in hypothalamic amenorrhea and anorexia nervosa. J. Clin. Endocrinol. Metab. 94, 4710–4716 10.1210/jc.2009-0153 19837921 PMC2795653

[B194] Lawson E. A. Donoho D. A. Blum J. I. Meenaghan E. M. Misra M. Herzog D. B. (2011a). Decreased nocturnal oxytocin levels in anorexia nervosa are associated with low bone mineral density and fat mass. J. Clin. Psychiatry 72, 1546–1551 10.4088/JCP.10m06617 21903023 PMC3731046

[B195] Lawson E. A. Eddy K. T. Donoho D. Misra M. Miller K. K. Meenaghan E. (2011b). Appetite-regulating hormones cortisol and peptide YY are associated with disordered eating psychopathology, independent of body mass index. Eur. J. Endocrinol. 164, 253–261 10.1530/EJE-10-0523 21098684 PMC3677777

[B196] Lawson E. A. Holsen L. M. Santin M. Meenaghan E. Eddy K. T. Becker A. E. (2012). Oxytocin secretion is associated with severity of disordered eating psychopathology and insular cortex hypoactivation in anorexia nervosa. J. Clin. Endocrinol. Metab. 97, E1898–E1908 10.1210/jc.2012-1702 22872688 PMC3674290

[B197] Lawson E. A. Klibanski A. (2008). Endocrine abnormalities in anorexia nervosa. Nat. Clin. Pract. Endocrinol. Metab. 4, 407–414 10.1038/ncpendmet0872 18542109

[B198] Legroux-Gerot I. Vignau J. Collier F. Cortet B. (2005). Bone loss associated with anorexia nervosa. Joint Bone Spine 72, 489–495 10.1016/j.jbspin.2004.07.011 16242373

[B199] Legroux-Gerot I. Vignau J. Collier F. Cortet B. (2008). Factors influencing changes in bone mineral density in patients with anorexia nervosa-related osteoporosis: the effect of hormone replacement therapy. Calcif. Tissue Int. 83, 315–323 10.1007/s00223-008-9173-y 18836675

[B200] Legroux-Gérot I. Vignau J. D’Herbomez M. Collier F. Marchandise X. Duquesnoy B. (2007). Evaluation of bone loss and its mechanisms in anorexia nervosa. Calcif. Tissue Int. 81, 174–182 10.1007/s00223-007-9038-9 17668143

[B201] Lesem M. D. Berrettini W. H. Kaye W. H. Jimerson D. C. (1991). Measurement of CSF dynorphin A 1-8 immunoreactivity in anorexia nervosa and normal-weight bulimia. Biol. Psychiatry 29, 244–252 10.1016/0006-3223(91)91286-Z 1673064

[B202] Lesem M. D. Kaye W. H. Bissette G. Jimerson D. C. Nemeroff C. B. (1994). Cerebrospinal fluid TRH immunoreactivity in anorexia nervosa. Biol. Psychiatry 35, 48–53 10.1016/0006-3223(94)91167-3 8167204

[B203] López M. Lage R. Saha A. K. Pérez-Tilve D. Vázquez M. J. Varela L. (2008). Hypothalamic fatty acid metabolism mediates the orexigenic action of ghrelin. Cell Metab. 7, 389–399 10.1016/j.cmet.2008.03.006 18460330

[B204] Löwe B. Zipfel S. Buchholz C. Dupont Y. Reas D. L. Herzog W. (2001). Long-term outcome of anorexia nervosa in a prospective 21-year follow-up study. Psychol. Med. 31, 881–890 10.1017/S003329170100407X 11459385

[B205] Lucas A. R. Beard C. M. O’Fallon W. M. Kurland L. T. (1991). 50-Year trends in the incidence of anorexia nervosa in Rochester, Minn.: a population-based study. Am. J. Psychiatry 148, 917–922 2053633 10.1176/ajp.148.7.917

[B206] Lucas A. R. Melton L. J. III Crowson C. S. O’Fallon W. M. (1999). Long-term frac-ture risk among women with anorexia nervosa: a population-based co-hort study. Mayo Clin. Proc. 74, 972–977 10.4065/74.10.972 10918862

[B207] Luckman S. M. Rosenzweig I. Dickson S. L. (1999). Activation of arcuate nucleus neurons by systemic administration of leptin and growth hormone-releasing peptide-6 in normal and fasted rats. Neuroendocrinology 70, 93–100 10.1159/000054463 10461023

[B208] Luquet S. Perez F. A. Hnasko T. S. Palmiter R. D. (2005). NPY/AgRP neurons are essential for feeding in adult mice but can be ablated in neonates. Science 310, 683–685 10.1126/science.1115524 16254186

[B209] Luquet S. Phillips C. T. Palmiter R. D. (2007). NPY/AgRP neurons are not essential for feeding responses to glucoprivation. Peptides 28, 214–225 10.1016/j.peptides.2006.08.036 17194499

[B210] Lutter M. Sakata I. Osborne-Lawrence S. Rovinsky S. A. Anderson J. G. Jung S. (2008). The orexigenic hormone ghrelin defends against depressive symptoms of chronic stress. Nat. Neurosci. 11, 752–763 10.1038/nn.2139 18552842 PMC2765052

[B211] Malik S. McGlone F. Bedrossian D. Dagher A. (2008). Ghrelin modulates brain activity in areas that control appetitive behavior. Cell Metab. 7, 400–509 10.1016/j.cmet.2008.03.007 18460331

[B212] Maltais L. J. Lane P. W. Beamer W. G. (1984). Anorexia, a recessive mutation causing starvation in preweanling mice. J. Hered. 75, 468–472 6595305 10.1093/oxfordjournals.jhered.a109987

[B213] Mano-Otagiri A. Ohata H. Iwasaki-Sekino A. Nemoto T. Shibasaki T. (2009). Ghrelin suppresses noradrenaline release in the brown adipose tissue of rats. J. Endocrinol. 201, 341–349 10.1677/JOE-08-0374 19351665

[B214] Mantzoros C. Flier J. S. Lesem M. D. Brewerton T. D. Jimerson D. C. (1997). Cerebrospinal fluid leptin in anorexia nervosa: correlation with nutritional status and potential role in resistance to weight gain. J. Clin. Endocrinol. Metab. 82, 1845–1851 10.1210/jc.82.6.1845 9177394

[B215] Martini A. C. Fernández-Fernández R. Tovar S. Navarro V. M. Vigo E. Vazquez M. J. (2006). Comparative analysis of the effects of ghrelin and unacylated ghrelin on luteinizing hormone secretion in male rats. Endocrinology 147, 2374–2382 10.1210/en.2005-1422 16455774

[B216] McKee K. K. Tan C. P. Palyha O. C. Liu J. Feighner S. D. Hreniuk D. L. (1997). Cloning and characterization of two human G protein-coupled receptor genes (GPR38 and GPR39) related to the growth hormone secretagogue and neurotensin receptors. Genomics 46, 426–434 10.1006/geno.1997.5069 9441746

[B217] Meczekalski B. Podfigurna-Stopa A. Warenik-Szymankiewicz A. Genazzani A. R. (2008). Functional hypothalamic amenorrhea: current view on neuroendocrine aberrations. Gynecol. Endocrinol. 24, 4–11 10.1080/09513590701807381 18224538

[B218] Mercader J. M. Ribasés M. Gratacòs M. González J. R. Bayés M. de Cid R. (2007). Altered brain-derived neurotrophic factor blood levels and gene variability are associated with anorexia and bulimia. Genes Brain Behav. 6, 706–716 10.1111/j.1601-183X.2007.00301.x 17376155

[B219] Merkestein M. Brans M. A. Luijendijk M. C. de Jong J. W. Egecioglu E. Dickson S. L. (2012). Ghrelin mediates anticipation to a palatable meal in rats. Obesity (Silver Spring) 20, 963–971 10.1038/oby.2011.389 22282050

[B220] Merle J. V. Haas V. Burghardt R. Döhler N. Schneider N. Lehmkuhl U. (2011). Agouti-related protein in patients with acute and weight-restored anorexia nervosa. Psychol. Med. 41, 2183–2192 10.1017/S0033291711000365 21426605

[B221] Mika C. Holtkamp K. Heer M. Günther R. W. Herpertz-Dahlmann B. (2007). A 2-year prospective study of bone metabolism and bone mineral density in adolescents with anorexia nervosa. J. Neural Transm. 114, 1611–1618 10.1007/s00702-007-0787-4 17676429

[B222] Miljic D. Pekic S. Djurovic M. Doknic M. Milic N. Casanueva F. F. (2006). Ghrelin has partial or no effect on appetite, growth hormone, prolactin, and cortisol release in patients with anorexia nervosa. J. Clin. Endocrinol. Metab. 91, 1491–1495 10.1210/jc.2005-2304 16449333

[B223] Miller K. K. (2011). Endocrine dysregulation in anorexia nervosa update. J. Clin. Endocrinol. Metab. 96, 2939–2949 10.1210/jc.2011-0380 21976742 PMC3200238

[B224] Misra M. Klibanski A. (2011). The neuroendocrine basis of anorexia nervosa and its impact on bone metabolism. Neuroendocrinology 93, 65–73 10.1159/000323771 21228564 PMC3214929

[B225] Misra M. Miller K. K. Almazan C. Ramaswamy K. Aggarwal A. Herzog D. B. (2004a). Hormonal and body composition predictors of soluble leptin receptor, leptin, and free leptin index in adolescent girls with anorexia nervosa and controls and relation to insulin sensitivity. J. Clin. Endocrinol. Metab. 89, 3486–3495 10.1210/jc.2004-0723 15240636

[B226] Misra M. Miller K. K. Almazan C. Ramaswamy K. Lapcharoensap W. Worley M. (2004b). Alterations in cortisol secretory dynamics in adolescent girls with anorexia nervosa and effects on bone metabolism. J. Clin. Endocrinol. Metab. 89, 4972–4980 10.1210/jc.2004-0723 15472193

[B227] Misra M. Miller K. Herzog D. Ramaswamy K. Aggarwal A. Almzan C. (2004c). Growth hormone and ghrelin responses to an oral glucose load in adolescent girls with anorexia nervosa and controls. J. Clin. Endocrinol. Metab. 89, 1605–1612 10.1210/jc.2004-0723 15070919

[B228] Misra M. Miller K. K. Bjornson J. Hackman A. Aggarwal A. Chung J. (2003). Alterations in growth hormone secretory dynamics in adolescent girls with anorexia nervosa and effects on bone metabolism. J. Clin. Endocrinol. Metab. 88, 5615–5623 10.1210/jc.2002-020889 14671143

[B229] Misra M. Miller K. K. Cord J. Prabhakaran R. Herzog D. B. Goldstein M. (2007). Relationships between serum adipokines, insulin levels, and bone density in girls with anorexia nervosa. J. Clin. Endocrinol. Metab. 92, 2046–2052 10.1210/jc.2006-2855 17356044

[B230] Misra M. Miller K. K. Kuo K. Griffin K. Stewart V. Hunter E. (2005a). Secretory dynamics of leptin in adolescent girls with anorexia nervosa and healthy adolescents. Am. J. Physiol. Endocrinol. Metab. 289, E373–E381 10.1152/ajpendo.00041.2005 15811876

[B231] Misra M. Miller K. K. Stewart V. Hunter E. Kuo K. Herzog D. B. (2005b). Ghrelin and bone metabolism in adolescent girls with anorexia nervosa and healthy adolescents. J. Clin. Endocrinol. Metab. 90, 5082–5087 10.1210/jc.2004-2041 15998770

[B232] Misra M. Miller K. K. Kuo K. Griffin K. Stewart V. Hunter E. (2005c). Secretory dynamics of ghrelin in adolescent girls with anorexia nervosa and healthy adolescents. Am. J. Physiol. Endocrinol. Metab. 289, E347–E356 10.1152/ajpendo.00041.2005 15755766

[B233] Misra M. Miller K. K. Tsai P. Gallagher K. Lin A. Lee N. (2006). Elevated peptide YY levels in adolescent girls with anorexia nervosa. J. Clin. Endocrinol. Metab. 91, 1027–1033 10.1210/jc.2005-1878 16278259

[B234] Misra M. Prabhakaran R. Miller K. K. Goldstein M. A. Mickley D. Clauss L. (2008). Prognostic indicators of changes in bone density measures in adolescent girls with anorexia nervosa-II. J. Clin. Endocrinol. Metab. 93, 1292–1297 10.1210/jc.2007-2419 18089697 PMC2291487

[B235] Modan-Moses D. Stein D. Pariente C. Yaroslavsky A. Ram A. Faigin M. (2007). Modulation of adiponectin and leptin during refeeding of female anorexia nervosa patients. J. Clin. Endocrinol. Metab. 92, 1843–1847 10.1210/jc.2006-1683 17327386

[B236] Modirrousta M. Mainville L. Jones B. E. (2005). Orexin and MCH neurons express c-Fos differently after sleep deprivation vs. recovery and bear different adrenergic receptors. Eur. J. Neurosci. 21, 2807–2816 10.1111/j.1460-9568.2005.04104.x 15926928

[B237] Mokrosinski J. Holst B. (2010). Modulation of the constitutive activity of the ghrelin receptor by use of pharmacological tools and mutagenesis. Meth. Enzymol. 484, 53–73 10.1016/B978-0-12-381298-8.00003-4 21036226

[B238] Monteleone P. (2011). New frontiers in endocrinology of eating disorders. Curr. Top. Behav. Neurosci. 6, 189–208 10.1007/7854_2010_87 21243477

[B239] Monteleone P. Di Lieto A. Tortorella A. Longobardi N. Maj M. (2000). Circulating leptin in patients with anorexia nervosa, bulimia nervosa or binge-eating disorder: relationship to body weight, eating patterns, psychopathology and endocrine changes. Psychiatry Res. 94, 121–129 10.1016/S0165-1781(00)00144-X 10808037

[B240] Monteleone P. Fabrazzo M. Martiadis V. Serritella C. Pannuto M. Maj M. (2005). Circulating brain-derived neurotrophic factor is decreased in women with anorexia and bulimia nervosa but not in women with binge-eating disorder: relationships to co-morbid depression, psychopathology and hormonal variables. Psychol. Med. 35, 897–905 10.1017/S0033291704003368 15997610

[B241] Monteleone P. Fabrazzo M. Tortorella A. Fuschino A. Maj M. (2002a). Opposite modifications in circulating leptin and soluble leptin receptor across the eating disorder spectrum. Mol. Psychiatry 7, 641–646 10.1038/sj.mp.4001043 12140788

[B242] Monteleone P. Martiadis V. Colurcio B. Maj M. (2002b). Leptin secretion is related to chronicity and severity of the illness in bulimia nervosa. Psychosom. Med. 64, 874–879 10.1097/01.PSY.0000024239.11538.A5 12461192

[B243] Monteleone P. Luisi M. Colurcio B. Casarosa E. Monteleone P. Ioime R. (2001). Plasma levels of neuroactive steroids are increased in untreated women with anorexia nervosa or bulimia nervosa. Psychosom. Med. 63, 62–68 11211066 10.1097/00006842-200101000-00008

[B244] Monteleone P. Tortorella A. Martiadis V. Serritella C. Fuschino A. Maj M. (2004). Opposite changes in the serum brain-derived neurotrophic factor in anorexia nervosa and obesity. Psychosom. Med. 66, 744–748 10.1097/01.psy.0000138119.12956.99 15385700

[B245] Morita S. Miyata S. (2012). Different vascular permeability between the sensory and secretory circumventricular organs of adult mouse brain. Cell Tissue Res. 349, 589–603 10.1007/s00441-012-1421-9 22584508

[B246] Moriya J. Takimoto Y. Yoshiuchi K. Shimosawa T. Akabayashi A. (2006). Plasma agouti-related protein levels in women with anorexia nervosa. Psychoneuroendocrinology 31, 1057–1061 10.1016/j.psyneuen.2006.06.006 16904835

[B247] Muccioli G. Lorenzi T. Lorenzi M. Ghè C. Arnoletti E. Raso G. M. (2011). Beyond the metabolic role of ghrelin: a new player in the regulation of reproductive function. Peptides 32, 2514–2521 10.1016/j.peptides.2011.10.020 22074955

[B248] Mullier A. Bouret S. G. Prevot V. Dehouck B. (2010). Differential distribution of tight junction proteins suggests a role for tanycytes in blood-hypothalamus barrier regulation in the adult mouse brain. J. Comp. Neurol. 518, 943–962 10.1002/cne.22273 20127760 PMC2892518

[B249] Muñoz-Calvo M. T. Barrios V. García de Alvaro M. T. Lefort M. Méndez-Dávila C. Argente J. (2007). Maintained malnutrition produces a progressive decrease in (OPG)/RANKL ratio and leptin levels in patients with anorexia nervosa. Scand. J. Clin. Lab. Invest. 67, 387–393 10.1080/00365510601110130 17558893

[B250] Nakahara T. Harada T. Yasuhara D. Shimada N. Amitani H. Sakoguchi T. (2008). Plasma obestatin concentrations are negatively correlated with body mass index, insulin resistance index, and plasma leptin concentrations in obesity and anorexia nervosa. Biol. Psychiatry 64, 252–255 10.1016/j.biopsych.2007.08.005 17919459

[B251] Nakahara T. Kojima S. Tanaka M. Yasuhara D. Harada T. Sagiyama K. (2007). Incomplete restoration of the secretion of ghrelin and PYY compared to insulin after food ingestion following weight gain in anorexia nervosa. J. Psychiatr. Res. 41, 814–820 10.1016/j.jpsychires.2006.07.021 17054989

[B252] Nakai Y. Hosoda H. Nin K. Ooya C. Hayashi H. Akamizu T. (2003). Plasma levels of active form of ghrelin during oral glucose tolerance test in patients with anorexia nervosa. Eur. J. Endocrinol. 149, R1–R3 10.1530/eje.0.1490001 12824869

[B253] Nakazato M. Hashimoto K. Shimizu E. Kumakiri C. Koizumi H. Okamura N. (2003). Decreased levels of serum brain-derived neurotrophic factor in female patients with eating disorders. Biol. Psychiatry 54, 485–490 10.1016/S0006-3223(02)01746-8 12915293

[B254] Nakazato M. Hashimoto K. Yoshimura K. Hashimoto T. Shimizu E. Iyo M. (2006). No change between the serum brain-derived neurotrophic factor in female patients with anorexia nervosa before and after partial weight recovery. Prog. Neuropsychopharmacol. Biol. Psychiatry 30, 1117–1121 10.1016/j.pnpbp.2006.04.017 16753247

[B255] Nakazato M. Murakami N. Date Y. Kojima M. Matsuo H. Kangawa K. (2001). A role for ghrelin in the central regulation of feeding. Nature 409, 194–198 10.1038/35051587 11196643

[B256] Nakazato M. Tchanturia K. Schmidt U. Campbell I. C. Treasure J. Collier D. A. (2009). Brain-derived neurotrophic factor (BDNF) and set-shifting in currently ill and recovered anorexia nervosa (AN) patients. Psychol. Med. 39, 1029–1035 10.1017/S0033291708004108 18752728

[B257] Naleid A. M. Grace M. K. Cummings D. E. Levine A. S. (2005). Ghrelin induces feeding in the mesolimbic reward pathway between the ventral tegmental area and the nucleus accumbens. Peptides 26, 2274–2279 10.1016/j.peptides.2005.04.025 16137788

[B258] Neary N. M. Small C. J. Wren A. M. Lee J. L. Druce M. R. Palmieri C. (2004). Ghrelin increases energy intake in cancer patients with impaired appetite: acute, randomized, placebo controlled trial. J. Clin. Endocrinol. Metab. 89, 2832–2836 10.1210/jc.2003-031768 15181065

[B259] Nedvidkova J. Krykorkova I. Bartak V. Papezova H. Gold P. W. Alesci S. (2003). Loss of meal-induced decrease in plasma ghrelin levels in patients with anorexia nervosa. J. Clin. Endocrinol. Metab. 88, 1678–1682 10.1210/jc.2002-021669 12679456

[B260] Nedvidkova J. Papezova H. Haluzik M. Schreiber V. (2000). Interaction between serum leptin levels and hypothalamo-hypophyseal-thyroid axis in patients with anorexia nervosa. Endocr. Res. 26, 219–230 10.3109/07435800009066163 10921449

[B261] Nemeroff C. B. Bissette G. Widerlov E. Beckmann H. Gerner R. Manberg P. J. (1989). Neurotensin-like immunoreactivity in cerebrospinal fluid of patients with schizophrenia, depression, anorexia nervosa-bulimia, and premenstrual syndrome. J. Neuropsychiatry Clin. Neurosci. 1, 16–20 2577718 10.1176/jnp.1.1.16

[B262] Nestler E. J. (1996). Under siege: the brain on opiates. Neuron 16, 897–900 10.1016/S0896-6273(00)80110-5 8630245

[B263] Nikolopoulos D. Theocharis S. Kouraklis G. (2010). Ghrelin, another factor affecting bone metabolism. Med. Sci. Monit. 16, RA147–RA162 20581789

[B264] Nilsson I. A. Thams S. Lindfors C. Bergstrand A. Cullheim S. Hökfelt T. (2011). Evidence of hypothalamic degeneration in the anorectic anx/anx mouse. Glia 59, 45–57 10.1002/glia.21075 20967882

[B265] Nobili L. Baglietto M. G. Beelke M. De Carli F. Di Comite R. Fiocchi I. (2004). Impairment of the production of delta sleep in anorectic adolescents. Sleep 27, 1553–1559 15683147 10.1093/sleep/27.8.1553

[B266] Nogal P. Pniewska-Siark B. Lewinski A. (2008). Evaluation of selected clinical and diagnostic parameters in girls with anorexia nervosa (I). Neuro Endocrinol. Lett. 29, 421–427 18766154

[B267] Nogueira J. P. Maraninchi M. Lorec A. M. Corroller A. B. Nicolay A. Gaudart J. (2010). Specific adipocytokines profiles in patients with hyperactive and/or binge/purge form of anorexia nervosa. Eur. J. Clin. Nutr. 64, 840–844 10.1038/ejcn.2010.66 20485307

[B268] Nova E. Marcos A. (2006). Immunocompetence to assess nutritional status in eating disorders. Expert Rev. Clin. Immunol. 2, 433–444 10.1586/1744666X.2.3.433 20476914

[B269] Ogiso K. Asakawa A. Amitani H. Inui A. (2011). Ghrelin and anorexia nervosa: a psychosomatic perspective. Nutrition 27, 988–993 10.1016/j.nut.2011.05.005 21868197

[B270] Ohwada R. Hotta M. Oikawa S. Takano K. (2006). Etiology of hypercholesterolemia in patients with anorexia nervosa. Int. J. Eat. Disord. 39, 598–601 10.1002/eat.20298 16791856

[B271] Ohwada R. Hotta M. Sato K. Shibasaki T. Takano K. (2007). The relationship between serum levels of estradiol and osteoprotegerin in patients with anorexia nervosa. Endocr. J. 54, 953–959 10.1507/endocrj.K07-034 17998762

[B272] Olatunji B. O. Broman-Fulks J. J. Ciesielski B. G. Zawilinski L. L. Shewmaker S. Wall D. (2012). A taxometric investigation of the latent structure of eating disorders. Psychiatry Res. 197, 97–102 10.1016/j.psychres.2011.12.016 22406392

[B273] Olszewski P. K. Li D. Grace M. K. Billington C. J. Kotz C. M. Levine A. S. (2003). Neural basis of orexigenic effects of ghrelin acting within lateral hypothalamus. Peptides 24, 597–602 10.1016/S0196-9781(03)00159-1 12860204

[B274] Onur S. Haas V. Bosy-Westphal A. Hauer M. Paul T. Nutzinger D. (2005). L-tri-iodothyronine is a major determinant of resting energy expenditure in underweight patients with anorexia nervosa and during weight gain. Eur. J. Endocrinol. 152, 179–184 10.1530/eje.1.01850 15745923

[B275] Osterstock G. Escobar P. Mitutsova V. Gouty-Colomer L. A. Fontanaud P. Molino F. (2010). Ghrelin stimulation of growth hormone-releasing hormone neurons is direct in the arcuate nucleus. PLoS ONE 5:e9159 10.1371/journal.pone.0009159 20161791 PMC2820089

[B276] Ostrowska Z. Ziora K. Kos-Kudla B. Swietochowska E. Oswiecimska J. Dyduch A. (2010). Melatonin, the RANKL/RANK/OPG system, and bone metabolism in girls with anorexia nervosa. Endokrynol. Pol. 61, 117–123 20205114

[B277] Ostrowska Z. Ziora K. Oswiecimska J. Swietochowska E. Wolkowska-Pokrywa K. (2012a). Dehydroepiandrosterone sulfate, osteoprotegerin and its soluble ligand sRANKL and bone metabolism in girls with anorexia nervosa. Postepy Hig. Med. Dosw. (Online) 66, 655–662 10.5604/17322693.1009971 23001207

[B278] Ostrowska Z. Ziora K. Oswiecimska J. Swietochowska E. Szapska B. Wolkowska-Pokrywa K. (2012b). RANKL/RANK/OPG system and bone status in females with anorexia nervosa. Bone 50, 156–160 10.1016/j.bone.2012.02.488 22001124

[B279] Oswiecimska J. Ziora K. Adamczyk P. Roczniak W. Pikiewicz-Koch A. Stojewska M. (2007). Effects of neuroendocrine changes on results of ambulatory blood pressure monitoring (ABPM) in adolescent girls with anorexia nervosa. Neuro Endocrinol. Lett. 28, 410–416 17693988

[B280] Otto B. Cuntz U. Fruehauf E. Wawarta R. Folwaczny C. Riepl R. L. (2001). Weight gain decreases elevated plasma ghrelin concentrations of patients with anorexia nervosa. Eur. J. Endocrinol. 145, 669–673 10.1530/eje.0.1450669 11720888

[B281] Otto B. Cuntz U. Otto C. Heldwein W. Riepl R. L. Tschop M. H. (2007). Peptide YY release in anorectic patients after liquid meal. Appetite 48, 301–304 10.1016/j.appet.2006.06.008 17157959

[B282] Otto B. Tschop M. Frühauf E. Heldwein W. Fichter M. Otto C. (2005). Postprandial ghrelin release in anorectic patients before and after weight gain. Psychoneuroendocrinology 30, 577–581 10.1016/j.psyneuen.2005.01.009 15808927

[B283] Palyha O. C. Feighner S. D. Tan C. P. McKee K. K. Hreniuk D. L. Gao Y. D. (2000). Ligand activation domain of human orphan growth hormone (GH) secretagogue receptor (GHS-R) conserved from Pufferfish to humans. Mol. Endocrinol. 14, 160–169 10.1210/me.14.1.160 10628755

[B284] Pannacciulli N. Vettor R. Milan G. Granzotto M. Catucci A. Federspil G. (2003). Anorexia nervosa is characterized by increased adiponectin plasma levels and reduced nonoxidative glucose metabolism. J. Clin. Endocrinol. Metab. 88, 1748–1752 10.1210/jc.2002-021215 12679468

[B285] Papotti M. Ghè C. Cassoni P. Catapano F. Deghenghi R. Ghigo E. (2000). Growth hormone secretagogue binding sites in peripheral human tissues. Clin. Endocrinol. Metab. 85, 3803–3807 10.1210/jc.85.10.3803 11061542

[B286] Pardo M. Roca-Rivada A. Al-Massadi O. Seoane L. M. Camiña J. P. Casanueva F. F. (2010). Peripheral leptin and ghrelin receptors are regulated in a tissue-specific manner in activity-based anorexia. Peptides 31, 1912–1919 10.1016/j.peptides.2010.06.022 20600421

[B287] Peeters T. L. (2003). Central and peripheral mechanisms by which ghrelin regulates gut motility. J. Physiol. Pharmacol. 54(Suppl. 4), 95–103 15075452

[B288] Peino R. Baldelli R. Rodriguez-Garcia J. Rodriguez-Segade S. Kojima M. Kangawa K. (2000). Ghrelin – induced growth hormone secretion in humans. Eur. J. Endocrinol. 143, R11–R14 10.1530/eje.0.143R011 11124868

[B289] Pemberton C. J. Richards A. M. (2007). Biochemistry of ghrelin precursor peptides. Vitam. Horm. 77, 13–30 10.1016/S0083-6729(06)77002-9 17983851

[B290] Perello M. Sakata I. Birnbaum S. Chuang J. C. Osborne-Lawrence S. Rovinsky S. A. (2010). Ghrelin increases the rewarding value of high-fat diet in an orexin-dependent manner. Biol. Psychiatry 67, 880–886 10.1016/j.biopsych.2009.10.030 20034618 PMC2854245

[B291] Perelló M. Zigman J. M. (2012). The role of ghrelin in reward-based eating. Biol. Psychiatry 72, 347–353 10.1016/j.biopsych.2012.02.016 22458951 PMC3388148

[B292] Petersen P. S. Woldbye D. P. Madsen A. N. Egerod K. L. Jin C. Lang M. (2009). In vivo characterization of high Basal signaling from the ghrelin receptor. Endocrinology 150, 4920–4930 10.1210/en.2008-1638 19819980

[B293] Phillipp E. Pirke K. M. Kellner M. B. Krieg J. C. (1991). Disturbed cholecystokinin secretion in patients with eating disorders. Life Sci. 48, 2443–2450 10.1016/0024-3205(91)90379-P 2046469

[B294] Pirke K. M. Friess E. Kellner M. B. Krieg J. C. Fichter M. M. (1994). Somatostatin in eating disorders. Int. J. Eat. Disord. 15, 99–102 10.1002/1098-108X(199401)15:1<63::AID-EAT2260150108>3.0.CO;2-V 7907255

[B295] Polli N. Scacchi M. Pecori Giraldi F. Sormani M. Zappulli D. Cavagnini F. (2008). Low insulin-like growth factor I and leukopenia in anorexia nervosa. Int. J. Eat. Disord. 41, 355–359 10.1002/eat.20506 18213689

[B296] Popovic V. Djurovic M. Cetkovic A. Vojvodic D. Pekic S. Spremovic S. (2004). Inhibin B: a potential marker of gonadal activity in patients with anorexia nervosa during weight recovery. J. Clin. Endocrinol. Metab. 89, 1838–1843 10.1210/jc.2003-031326 15070953

[B297] Putignano P. Dubini A. Toja P. Invitti C. Bonfanti S. Redaelli G. (2001). Salivary cortisol measurement in normal-weight, obese and anorexic women: comparison with plasma cortisol. Eur. J. Endocrinol. 145, 165–171 10.1530/eje.0.1450165 11454512

[B298] Qader S. S. Håkanson R. Rehfeld J. F. Lundquist I. Salehi A. (2008). Proghrelin-derived peptides influence the secretion of insulin, glucagon, pancreatic polypeptide and somatostatin: a study on isolated islets from mouse and rat pancreas. Regul. Pept. 146, 230–237 10.1016/j.regpep.2007.09.017 17942170

[B299] Repaci A. Gambineri A. Pagotto U. Pasquali R. (2011). Ghrelin and reproductive disorders. Mol. Cell. Endocrinol. 340, 70–79 10.1016/j.mce.2011.02.022 21453749

[B300] Rodríguez A. Gómez-Ambrosi J. Catalán V. Gil M. J. Becerril S. Sáinz N. (2009). Acylated and desacyl ghrelin stimulate lipid accumulation in human visceral adipocytes. Int. J. Obes. (Lond.) 33, 541–552 10.1038/ijo.2009.40 19238155

[B301] Routtenberg A. Kuznesof A. W. (1967). Self-starvation of rats living in activity wheels on a restricted feeding schedule. J. Comp. Physiol. Psychol. 64, 414–421 10.1037/h0025205 6082873

[B302] Saito S. Watanabe K. Hashimoto E. Saito T. (2009). Low serum BDNF and food intake regulation: a possible new explanation of the pathophysiology of eating disorders. Prog. Neuropsychopharmacol. Biol. Psychiatry 33, 312–316 10.1016/j.pnpbp.2008.12.009 19152823

[B303] Samec S. Seydoux J. Russell A. P. Montani J. P. Dulloo A. G. (2002). Skeletal muscle heterogeneity in fasting-induced upregulation of genes encoding UCP2, UCP3, PPARgamma and key enzymes of lipid oxidation. Pflugers Arch. 445, 80–86 10.1007/s00424-002-0879-9 12397391

[B304] Sanger G. J. (2008). Motilin, ghrelin and related neuropeptides as targets for the treatment of GI diseases. Drug Discov. Today 13, 234–239 10.1016/j.drudis.2007.10.024 18342799

[B305] Sangiao-Alvarellos S. Cordido F. (2010). Effect of ghrelin on glucose-insulin homeostasis: therapeutic implications. Int. J. Pept. 2010, pii: 234709. 10.1155/2010/234709 PMC2911604 20700401

[B306] Schellekens H. Dinan T. G. Cryan J. F. (2010). Lean mean fat reducing “ghrelin” machine: hypothalamic ghrelin and ghrelin receptors as therapeutic targets in obesity. Neuropharmacology 58, 2–16 10.1016/j.neuropharm.2009.06.024 19573543

[B307] Sedlackova D. Kopeckova J. Papezova H. Hainer V. Kvasnickova H. Hill M. (2012). Comparison of a high-carbohydrate and highprotein breakfast effect on plasma ghrelin, obestatin, NPY and PYY levels in women with anorexia and bulimia nervosa. Nutr. Metab. (Lond.) 9, 52 10.1186/1743-7075-9-52 22681985 PMC3533897

[B308] Shimizu N. Oomura Y. Kai Y. (1989). Stress-induced anorexia in rats mediated by serotonergic mechanisms in the hypothalamus. Physiol. Behav. 46, 835–841 10.1016/0031-9384(89)90045-0 2628995

[B309] Shrestha Y. B. Wickwire K. Giraudo S. (2009). Effect of reducing hypothalamic ghrelin receptor gene expression on energy balance. Peptides 30, 1336–1341 10.1016/j.peptides.2009.02.001 19540432 PMC2700781

[B310] Siegfried Z. Berry E. M. Hao S. Avraham Y. (2003). Animal models in the investigation of anorexia. Physiol. Behav. 79, 39–45 10.1016/S0031-9384(03)00103-3 12818708

[B311] Skibicka K. P. Dickson S. L. (2011). Ghrelin and food reward: the story of potential underlying substrates. Peptides 32, 2265–2273 10.1016/j.peptides.2011.05.016 21621573

[B312] Skibicka K. P. Hansson C. Egecioglu E. Dickson S. L. (2012). Role of ghrelin in food reward: impact of ghrelin on sucrose self-administration and mesolimbic dopamine and acetylcholine receptor gene expression. Addict. Biol. 17, 95–107 10.1111/j.1369-1600.2010.00294.x 21309956 PMC3298643

[B313] Smith G. P. (1989). Animal models of human eating disorders. Ann. N. Y. Acad. Sci. 575, 63–72 10.1111/j.1749-6632.1989.tb53233.x 2699206

[B314] Soriano-Guillen L. Barrios V. Campos-Barros A. Argente J. (2004). Ghrelin levels inobesityand anorexia nervosa: effect of weight reduction or recuperation. J. Pediatr. 144, 36–42 10.1016/j.jpeds.2003.10.036 14722516

[B315] Speranza M. Revah-Levy A. Giquel L. Loas G. Venisse J. L. Jeammet P. (2012). An investigation of Goodman’s addictive disorder criteria in eating disorders. Eur. Eat. Disord. Rev. 20, 182–189 10.1002/erv.1140 21834026

[B316] Stengel A. Keire D. Goebel M. Evilevitch L. Wiggins B. Taché Y. (2009). The RAPID method for blood processing yields new insight in plasma concentrations and molecular forms of circulating gut peptides. Endocrinology 150, 5113–5118 10.1210/en.2009-0578 19819958 PMC2775981

[B317] Stock S. Leichner P. Wong A. C. Ghatei M. A. Kieffer T. J. Bloom S. R. (2005). Ghrelin, peptide YY, glucose-dependent insulinotropic polypeptide, and hunger responses to a mixed meal in anorexic, obese, and control female adolescents. J. Clin. Endocrinol. Metab. 90, 2161–2168 10.1210/jc.2004-1251 15657373

[B318] Støving R. K. Chen J. W. Glintborg D. Brixen K. Flyvbjerg A. Hørder K. (2007). Bioactive insulin-like growth factor (IGF) I and IGF-binding protein-1 in anorexia nervosa. J. Clin. Endocrinol. Metab. 92, 2323–2329 10.1210/jc.2006-1926 17389700

[B319] Støving R. K. Hangaard J. Hagen C. Flyvbjerg A. (2003). Low levels of the 150-kD insulin-like growth factor binding protein 3 ternary complex in patients with anorexia nervosa: effect of partial weight recovery. Horm. Res. 60, 43–48 10.1159/000070826 12792153

[B320] Støving R. K. Veldhuis J. D. Flyvbjerg A. Vinten J. Hangaard J. Koldkjaer O. G. (1999). Jointly amplified basal and pulsatile growth hormone (GH) secretion and increased process irregularity in women with anorexia nervosa: indirect evidence for disruption of feedback regulation within the GH-insulin-like growth factor I axis. J. Clin. Endocrinol. Metab. 84, 2056–2063 10.1210/jc.84.6.2056 10372710

[B321] Sun Y. Ahmed S. Smith R. G. (2003). Deletion of ghrelin impairs neither growth nor appetite. Mol. Cell. Biol. 23, 7973–7981 10.1128/MCB.23.22.7973-7981.2003 14585959 PMC262394

[B322] Sun Y. Butte N. F. Garcia J. M. Smith R. G. (2008). Characterization of adult ghrelin and ghrelin receptor knockout mice under positive and negative energy balance. Endocrinology 149, 843–850 10.1210/en.2007-0271 18006636 PMC2219310

[B323] Sun Y. Wang P. Zheng H. Smith R. G. (2004). Ghrelin stimulation of growth hormone release and appetite is mediated through the growth hormone secretagogue receptor. Proc. Natl. Acad. Sci. U.S.A. 101, 4679–4684 10.1073/pnas.0407576101 15070777 PMC384806

[B324] Svensson J. Lall S. Dickson S. L. Bengtsson B. A. Rømer J. Ahnfelt-Rønne I. (2000). The GH secretagogues ipamorelin and GH-releasing peptide-6 increase bone mineral content in adult female rats. J. Endocrinol. 165, 569–577 10.1677/joe.0.1650569 10828840

[B325] Szentirmai E. Kapás L. Sun Y. Smith R. G. Krueger J. M. (2009). The preproghrelin gene is required for the normal integration of thermoregulation and sleep in mice. Proc. Natl. Acad. Sci. U.S.A. 106, 14069–14074 10.1073/pnas.0903090106 19666521 PMC2729021

[B326] Tagami T. Satoh N. Usui T. Yamada K. Shimatsu A. Kuzuya H. (2004). Adiponectin in anorexia nervosa and bulimia nervosa. J. Clin. Endocrinol. Metab. 89, 1833–1837 10.1210/jc.2003-031260 15070952

[B327] Tamai H. Takemura J. Kobayashi N. Matsubayashi S. Matsukura S. Nakagawa T. (1993). Changes in plasma cholecystokinin concentrations after oral glucose tolerance test in anorexia nervosa before and after therapy. Metab. Clin. Exp. 42, 581–584 10.1016/0026-0495(93)90216-B 8492713

[B328] Tanaka M. Nakahara T. Kojima S. Nakano T. Muranaga T. Nagai N. (2004). Effect of nutritional rehabilitation on circulating ghrelin and growth hormone levels in patients with anorexia nervosa. Regul. Pept. 122, 163–168 10.1016/j.regpep.2004.06.015 15491786

[B329] Tanaka M. Tatebe Y. Nakahara T. Yasuhara D. Sagiyama K. Muranaga T. (2003a). Eating pattern and the effect of oral glucose on ghrelin and insulin secretion in patients with anorexia nervosa. Clin. Endocrinol. (Oxf.) 59, 574–549 10.1046/j.1365-2265.2003.01886.x 14616897

[B330] Tanaka M. Naruo T. Yasuhara D. Tatebe Y. Nagai N. Shiiya T. (2003b). Fasting plasma ghrelin levels in subtypes of anorexia nervosa. Psychoneuroendocrinology 28, 829–835 10.1016/S0306-4530(02)00066-5 12892652

[B331] Tanaka M. Narau T. Nagai N. Kuroki N. Shiiya T. Nakazato M. (2003c). Habitual binge/purge behavior influences circulating ghrelin levels in eating disorders. J. Psychiatr. Res. 37, 17–22 10.1016/S0022-3956(02)00067-5 12482466

[B332] Taub D. D. (2008). Novel connections between the neuroendocrine and immune systems: the ghrelin immunoregulatory network. Vitam. Horm. 77, 325–346 10.1016/S0083-6729(06)77014-5 17983863

[B333] Tena-Sempere M. (2008). Ghrelin as a pleotrophic modulator of gonadal function and reproduction. Nat. Clin. Pract. Endocrinol. Metab. 4, 666–674 10.1038/ncpendmet1003 18981992

[B334] Tepper R. Weizman A. Apter A. Tyano S. Beyth Y. (1992). Elevated plasma immunoreactive b-endorphin in anorexia nervosa. Clin. Neuropharmacol. 15, 387–391 10.1097/00002826-199202001-00752 1423337

[B335] Tesauro M. Schinzari F. Caramanti M. Lauro R. Cardillo C. (2010). Metabolic and cardiovascular effects of ghrelin. Int. J. Pept. 2010, 1–7 10.1155/2010/493614 PMC2925368 20798901

[B336] Thompson N. M. Gill D. A. Davies R. Loveridge N. Houston P. A. Robinson I. C. (2004). Ghrelin and des-octanoyl ghrelin promote adipogenesis directly in vivo by a mechanism independent of the type 1a growth hormone secretagogue receptor. Endocrinology 145, 234–242 10.1210/en.2003-0899 14551228

[B337] Tolle V. Bassant M. H. Zizzari P. Poindessous-Jazat F. Tomasetto C. Epelbaum J. (2002). Ultradian rhythmicity of ghrelin secretion in relation with GH, feeding behavior, and sleep-wake patterns in rats. Endocrinology 143, 1353–1361 10.1210/en.143.4.1353 11897692

[B338] Tolle V. Kadem M. Bluet-Pajot M. T. Frere D. Foulon C. Bossu C. (2003). Balance in ghrelin and leptin plasma levels in anorexia nervosa patients and constitutionally thin women. J. Clin. Endocrinol. Metab. 88, 109–116 10.1210/jc.2002-020645 12519838

[B339] Tomasik P. J. Sztefko K. Malek A. (2002). GLP-1 as a satiety factor in children with eating disorders. Horm. Metab. Res. 34, 77–80 10.1055/s-2002-20519 11972291

[B340] Tomasik P. J. Sztefko K. Starzyk J. (2004). Cholecystokinin, glucose dependent insulinotropic peptide and glucagon-like peptide 1 secretion in childrenwith anorexia nervosa and simple obesity. J. Pediatr. Endocrinol. Metab. 17, 1623–1631 10.1515/JPEM.2004.17.12.1623 15645696

[B341] Tomasik P. J. Sztefko K. Starzyk J. Rogatko I. Szafran Z. (2005). Entero-insular axis in children with anorexia nervosa. Psychoneuroendocrinology 30, 364–372 10.1016/j.psyneuen.2004.10.003 15694116

[B342] Tomova A. Makker K. Kirilov G. Agarwal A. Kumanov P. (2007). Disturbances in gonadal axis in women with anorexia nervosa. Eat. Weight Disord. 12, e92–e97 18227633 10.1007/BF03327602

[B343] Toshinai K. Date Y. Murakami N. Shimada M. Mondal M. S. Shimbara T. (2003). Ghrelin-induced food intake is mediated via the orexin pathway. Endocrinology 144, 1506–1512 10.1210/en.2002-220788 12639935

[B344] Toshinai K. Yamaguchi H. Sun Y. Smith R. G. Yamanaka A. Sakurai T. (2006). Des-acyl ghrelin induces food intake by a mechanism independent of the growth hormone secretagogue receptor. Endocrinology 147, 2306–2314 10.1210/en.2005-1357 16484324

[B345] Troisi A. Di Lorenzo G. Lega I. Tesauro M. Bertoli A. Leo R. (2005). Plasma ghrelin in anorexia, bulimia, and binge-eating disorder: relations with eating patterns and circulating concentrations of cortisol and thyroid hormones. Neuroendocrinology 81, 259–266 10.1159/000087923 16131812

[B346] Tschöp M. Smiley D. L. Heiman M. L. (2000). Ghrelin induces adiposity in rodents. Nature 407, 908–910 10.1038/35038090 11057670

[B347] Tschöp M. Wawarta R. Riepl R. L. Friedrich S. Bidlingmaier M. Landgraf R. (2001). Post-prandial decrease of circulating human ghrelin levels. J. Endocrinol. Invest. 24, RC19–RC21 11434675 10.1007/BF03351037

[B348] Tsubota Y. Owada-Makabe K. Yukawa K. Maeda M. (2005). Hypotensive effect of des-acyl ghrelin at nucleus tractus solitarii of rat. Neuroreport 16, 163–166 10.1097/00001756-200502080-00019 15671869

[B349] Uehara T. Omori I. Nakamura K. Suda M. Hosoda Y. Minegishi T. (2005). Plasma des-acyl and acyl ghrelin in patients with eating disorders. Eat. Weight Disord. 10, 264–266 16755171 10.1007/BF03327494

[B350] Uhe A. M. Szmukler G. I. Collier G. R. Hansky J. O’Dea K. Young G. P. (1992). Potential regulators of feeding behavior in anorexia nervosa. Am. J. Clin. Nutr. 55, 28–32 1728817 10.1093/ajcn/55.1.28

[B351] Valevski A. Fridberg G. Gil-Ad I. Laron Z. Weizman A. (2000). Neuroendocrinological response to standardized mixed meal in female anorectic patients during active and refeeding phases. J. Pediatr. Endocrinol. Metab. 13, 195–199 10.1515/JPEM.2000.13.2.195 10711666

[B352] Van Vugt D. A. (2010). Brain imaging studies of appetite in the context of obesity and the menstrual cycle. Hum. Reprod. Update 16, 276–292 10.1093/humupd/dmp051 19926661

[B353] Veldhuis J. D. Bowers C. Y. (2010). Integrating GHS into the ghrelin system. Int. J. Pept. 2010, 1–40 10.1155/2010/493614 PMC2925380 20798846

[B354] Verhagen L. A. Egecioglu E. Luijendijk M. C. Hillebrand J. J. Adan R. A. Dickson S. L. (2011). Acute and chronic suppression of the central ghrelin signaling system reveals a role in food anticipatory activity. Eur. Neuropsychopharmacol. 21, 384–392 10.1016/S0924-977X(11)70617-4 20620030

[B355] Viapiana O. Gatti D. Dalle Grave R. Todesco T. Rossini M. Braga V. (2007). Marked increases in bone mineral density and biochemical markers of bone turnover in patients with anorexia nervosa gaining weight. Bone 40, 1073–1077 10.1016/j.bone.2006.11.015 17240212

[B356] Volkov N. D. Wang G. J. Baler R. D. (2011). Reward, dopamine and the control of food intake: implications for obesity. Trends Cogn. Sci. (Regul. Ed.) 15, 37–46 10.1016/j.tics.2010.11.001 21109477 PMC3124340

[B357] Wang L. Saint-Pierre D. H. Tache Y. (2002). Peripheral ghrelin selectively increases Fos expression in neuropeptide Y-synthesizing neurons in mouse hypothalamic arcuate nucleus. Neurosci. Lett. 325, 47–51 10.1016/S0304-3940(02)00241-0 12023064

[B358] Wang T. Hung C. C. Randall D. J. (2006). The comparative physiology of food deprivation: from feast to famine. Annu. Rev. Physiol. 68, 223–251 10.1146/annurev.physiol.68.040104.105739 16460272

[B359] Weinbrenner T. Zittermann A. Gouni-Berthold I. Stehle P. Berthold H. K. (2003). Body mass index and disease duration are predictors of disturbed bone turnover in anorexia nervosa. A case-control study. Eur. J. Clin. Nutr. 57, 1262–1267 10.1038/sj.ejcn.1601683 14506487

[B360] Wellman P. J. Davis K. W. Nation J. R. (2005). Augmentation of cocaine hyperactivity in rats by systemic ghrelin. Regul. Pept. 125, 151–154 10.1016/j.regpep.2004.08.013 15582726

[B361] Willner P. (1984). The validity of animal models of depression. Psychopharmacology (Berl.) 83, 1–16 10.1007/BF00427414 6429692

[B362] Wong M. L. Licinio J. Gold P. W. Glowa J. (1993). Activity-induced anorexia in rats does not affect hypothalamic neuropeptide gene expression chronically. Int. J. Eat. Disord. 13, 399–405 10.1002/1098-108X(199305)13:4<399::AID-EAT2260130408>3.0.CO;2-J 8387855

[B363] Wortley K. E. Anderson K. D. Garcia K. Murray J. D. Malinova L. Liu R. (2004). Genetic deletion of ghrelin does not decrease food intake but influences metabolic fuel preference. Proc. Natl. Acad. Sci. U.S.A. 101, 8227–8232 10.1073/pnas.0402763101 15148384 PMC419585

[B364] Wortley K. E. del Rincon J. P. Murray J. D. Garcia K. Iida K. Thorner M. O. (2005). Absence of ghrelin protects against early-onset obesity. J. Clin. Invest. 115, 3573–3578 10.1172/JCI26003 16322795 PMC1297252

[B365] Wren A. M. Seal L. J. Cohen M. A. Brynes A. E. Frost G. S. Murphy K. G. (2001a). Ghrelin enhances appetite and increases food intake in humans. J. Clin. Endocrinol. Metab. 86, 5992 10.1210/jc.86.12.5992 11739476

[B366] Wren A. M. Small C. J. Abbott C. R. Dhillo W. S. Seal L. J. Cohen M. A. (2001b). Ghrelin causes hyperphagia and obesity in rats. Diabetes 50, 2540–2547 10.2337/diabetes.50.11.2540 11679432

[B367] Wren A. M. Small C. J. Ward H. L. Murphy K. G. Dakin C. L. Taheri S. (2000). The novel hypothalamic peptide ghrelin stimulates food intake and growth hormone secretion. Endocrinology 141, 4325–4328 10.1210/en.141.11.4325 11089570

[B368] Yang J. Brown M. S. Liang G. Grishin N. V. Goldstein J. L. (2008). Identification of the acyltransferase that octanoylates ghrelin, an appetite-stimulating peptide hormone. Cell 132, 387–396 10.1016/j.cell.2008.01.017 18267071

[B369] Yi C. X. Heppner K. Tschöp M. H. (2011). Ghrelin in eating disorders. Mol. Cell. Endocrinol. 340, 29–34 10.1016/j.mce.2011.03.001 21453750

[B370] Zhao C. P. Tang M. Peeters T. L. Depoortere I. (2003). In the rat ghrelin changes the excitability of neurons in the paraventricular nucleus of the hypothalamus. Neurogastroent. Mot. 15, 571–590 10.1046/j.1365-2982.2003.00440.x

[B371] Zhao T. J. Liang G. Li R. L. Xie X. Sleeman M. W. Murphy A. J. (2010). Ghrelin O-acyltransferase (GOAT) is essential for growth hormone-mediated survival of calorie-restricted mice. Proc. Natl. Acad. Sci. U.S.A. 107, 7467–7472 10.1073/pnas.1011116107 20231469 PMC2867684

[B372] Zhu X. Cao Y. Voogd K. Steiner D. F. (2006). On the processing of proghrelin to ghrelin. J. Biol. Chem. 281, 38867–38870 10.1074/jbc.M607191200 17050541

[B373] Zigman J. M. Jones J. E. Lee C. E. Saper C. B. Elmquist J. K. (2006). Expression of ghrelin receptor mRNA in the rat and the mouse brain. J. Comp. Neurol. 494, 528–548 10.1002/cne.20823 16320257 PMC4524499

[B374] Ziora K. T. Oswiecimska J. M. Swietochowska E. Ostrowska Z. Stojewska M. Gorczyca P. (2011). Assessment of serum levels resistin in girls with anorexia nervosa. Part II. Relationships between serum levels of resistin and thyroid, adrenal and gonadal hormones. Neuro Endocrinol. Lett. 32, 697–703 22167129

[B375] Zizzari P. Halem H. Taylor J. Dong J. Z. Datta R. Culler M. D. (2005). Endogenous ghrelin regulates episodic growth hormone (GH) secretion by amplifying GH Pulse amplitude: evidence from antagonism of the GH secretagogue-R1a receptor. Endocrinology 146, 3836–3842 10.1210/en.2005-0212 15919752

[B376] Zizzari P. Hassouna R. Longchamps R. Epelbaum J. Tolle V. (2011). Meal anticipatory rise in acylated ghrelin at dark onset is blunted after long-term fasting in rats. J. Neuroendocrinol. 23, 804–814 10.1111/j.1365-2826.2011.02183.x 21722214

